# Molybdooxaziridine–Molybdodioxirane Tandem Catalysis: Synthesis of α‑Nitrosulfones from Styrenes

**DOI:** 10.1021/acs.joc.6c00262

**Published:** 2026-04-21

**Authors:** Jenna M. Doran, Alyssa N. Singer, Steven J. Finneran, Gustavo Moura-Letts

**Affiliations:** Department of Chemistry and Biochemistry, 3536 Rowan University, 201 Mullica Hill Rd., Glassboro, New Jersey 08028, United States

## Abstract

α-Nitrosulfones are valuable molecular scaffolds. This effort reports the MoO_2_Dipic promoted α-nitrosulfonation of styrenes using *N*-Ts-hydroxylamine and H_2_O_2_. This process starts with the formation of metallooxaziridine **1a** that promotes the formation of oxime sulfone **5**, which then undergoes molybdodioxirane-promoted oxidation to α-nitrosulfone **3**. The reaction works with high yields and stereoselectivites for styrenes with a wide variety of substitution patterns. An atom transfer–radical addition mechanism involving the formation of a Ts radical, difunctionalization, nitroso-oxime isomerization, and final oxidation has been proposed. Initial kinetic and mechanistic data indicates a tandem catalytic process and provides evidence for the proposed mechanism.

## Graphical abstract

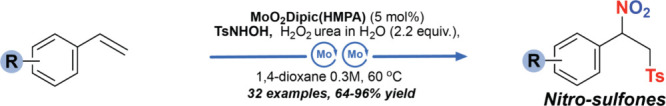

## Introduction

Metallooxaziridines are heterocyclic complexes that can be obtained from reacting ligand-bound metal-oxides and *N*-transfer reagents.^1^ These metalloheterocycles are known to achieve the functionalization of π-systems with high selectivities and through different reaction pathways.^2^ Recently, our research group discovered that metallooxaziridines can achieve different transformations across alkenes of different substitution patterns.^3^ This work led to the discovery that *N*-Ts-molybdooxaziridines at high temperatures promote the synthesis of vinyl sulfones from styrenes,^4^ and *N*-Ph-molybdooxaziridines promote the nitroso-Diels–Alder reaction of dienes. It was on this last effort that we discovered the competing formation of molybdodioxiranes and their role in these processes.^5^ Thus, the potential for discovering novel chemical pathways across metallooxaziridine/metallodioxirane tandem catalysis is wide open and of great significance to the field of organic chemistry.

Atom transfer radical additions (ATRA) have erupted as highly efficient and selective chemical processes for the functionalization of π-systems.^6^ Many reports focus on the activation of alkenes through metal-promoted halosulfonylations.^7^ Most of these process start with an initial Ts radical addition at high temperatures or through photocatalysis, followed by a second addition that is often poorly selective and leads to mixtures or less synthetically useful functionalities.^8^ ATRA reactions, as a pathway to selectively produce highly substituted scaffolds from simple alkenes, continue to represent a challenge among difunctionalization reactions and often require harsh reagents and complex substrates.

Nitroalkanes are valuable and versatile synthetic building blocks in organic chemistry, as they can be transformed into different functional groups with numerous available methods for such transformations.^9^ However, methods for their synthesis are scarce. Nitroalkanes can be obtained from the nitration of tertiary and secondary alkanes with nitronium hexafluorophosphate, or by reacting alkyl halides with silver nitrite in aqueous media.^10^ Moreover, Ishii and Yamaguchi have developed catalytic systems where nitric acid delivers nitro groups across cycloalkanes with good efficiencies.^11^ Substituted nitroalkanes can also undergo radical additions across alkenes to form complex functionalized molecules.^12^ Aryl nitroalkanes have received a lot of attention, and these can be made by reacting diaryliodonium salts with nitronate salts or nitroalkanes under strong basic conditions.^13^ There are also several studies on their synthesis via the transition-metal-promoted (Pd, Cu) cross-coupling of arylhalides and nitroalkanes.^14^ β-Nitrostyrenes have been reported to undergo sulfa-Michael additions with arylsulfinates or sulfonylhydrazides to provide β-nitrosulfones.^15^ However, the difunctionalization of styrenes for the synthesis of α-nitrosulfones has not been reported to date.

Tandem catalysis refers to two mechanistically discrete transformations performed in a given reaction with two (orthogonal tandem catalysis) catalysts.^16^ A key example of orthogonal tandem catalysis was developed by Brookhart and Goldman for alkane metathesis.^17^ In that system, iridium and ruthenium complexes promote the isomerization/metathesis of low-molecular-weight *n*-hexane to *n*-decane with high molecular selectivity for linear alkanes. Other efforts have coupled oxidations, isomerizations, and rearrangements with hydrogenation processes to effect unique tandem transformations.^18^ Thus, tandem catalysis allows for unique transformations to enable the synthesis of complex molecular architectures.

The Moura-Letts laboratory is focused on developing novel methods for the synthesis of complex heteroatom-containing molecules.^19^ It has been established that N–Ar and N–Ts molybdooxaziridines allow the formation of nitroso reagents.^20^ Thus, we envisioned creating a well-defined catalytic system that reacts across alkenes to achieve difunctionalization and under oxidative conditions to produce α-nitrosulfone **3**. To the best of our knowledge, this is the first example of this type of catalytic system and the first reaction to report the formation of α-nitrosulfones.

## Results and Discussion

Based on our previous studies, we knew that at high temperatures and concentrations vinyl sulfone **7** was the predominant reaction outcome (entry 1, Table 1);^4^ thus, we envisioned that at lower reaction temperatures and concentrations the formation of **7** will halt in favor of α-nitrosulfone **3**. However, the crude reaction showed a mixture of α-nitrosulfone **3**, hydroxysulfone **4**, and oxime sulfone **5** (CH_3_CN at 80 °C for 24h, entry 2). Previous efforts have discovered that MoO_2_Dipic­(HMPA) and tosylhydroxylamine undergoes fast insertion to produce molybdooxaziridine **1a**, while the H_2_O_2_ oxidation of MoO_2_Dipic­(HMPA) to molybdodioxirane **1b** can also be competitive. The formation of oxime **5** as a major product (entry 3) clearly indicated that ATRA was working, but nitrososulfone **6** isomerization to **5** was very fast. Careful analysis of crude reactions displayed a trace amount of **6**, but isolation efforts were not successful. Efforts to enhance the formation of α-nitrosulfone **3** by testing different solvents led to the discovery that 1,4-dioxane provides **3** as the major product (entries 2–7). The formation of **4** as a major side product in toluene (entry 5) was quite surprising, and it highlights the possibility that ATRA occurs via a molybdooxaziridine radical rather than initial formation of a tosyl nitroso intermediate.^4^ While the oxidation of **6** to **3** happens at high temperatures, the isomerization of **6** to **5** is known to be a fast process; thus, a second molybdoheterocycle complex is promoting the oxidation of **5** to **3**. A potential tandem catalytic process led us to address the conversion of oxime **5** to α-nitrosulfone **3**. To address the role of molybdodioxirane **1b**, we tested the effect of water in the reaction and discovered that H_2_O_2_·urea with additional H_2_O provided a 10:1 ratio in favor of α-nitrosulfone **3** (78% yield, entry 11). The crude reactions were still complex, and further optimization led to the discovery that at 60 °C and for 72 h, we could obtain **3** in 93% yield with only traces of **5** (entry 14). We then attempted to see if lower catalysts loading would further improve the reaction, but as expected due to the tandem nature of this process, selectivities dropped (entries 14 and 16). Other oxidants or combinations of catalysts/precatalysts failed to provide improved outcomes. Based on these results, we had an initial optimized reaction for the synthesis of α-nitrosulfones via a tandem catalytic process.

**Table 1:**
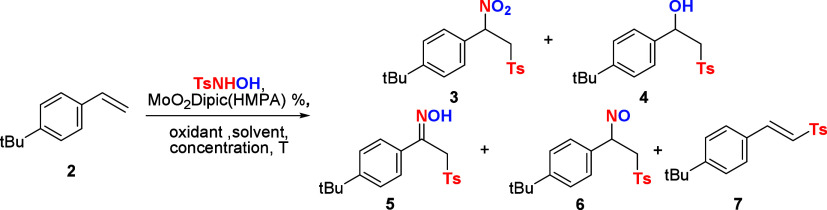
Reaction Optimization

Entry	Catalyst	Oxidant	Solvent	T/time	Ratio 3:4:5:6:7^h^	Yield (%)^a^ ^,^ ^b^
1	5 mol %	H_2_O_2_ ^c^	1,4-dioxane^g^	100 °C/16 h	1:0:0:0:20	88
2	5 mol %	H_2_O_2_ ^c^	CH_3_CN	80 °C/24 h	1:0.1:1:0.05	40
3	5 mol %	H_2_O_2_·urea^d^	CH_3_CN	80 °C/24 h	0:0:1:0	48
4	5 mol %	H_2_O_2_	CH_3_CN/H_2_O	80 °C/24 h	0.5:0.1:1:0	53
5	5 mol %	H_2_O_2_	Toluene	80 °C/24 h	0.5:0.5:1:0	44
6	5 mol %	H_2_O_2_	THF	80 °C/24 h	0.8:0.1:1:0.1	64
7	5 mol %	H_2_O_2_	DMA	80 °C/24 h	1.2:0:1:0.1	35
8	5 mol %	H_2_O_2_·urea	1,4-dioxane	80 °C/24 h	2:0.05:1:0	71
9	5 mol %	H_2_O_2_·urea:H_2_O^e^	1,4-dioxane	80 °C/24 h	2:0.1:0.5:0	68
10	5 mol %	H_2_O_2_·urea:H_2_O^f^	1,4-dioxane	80 °C/24 h	3:0.1:0.5:0	75
11	5 mol %	H_2_O_2_·urea:H_2_O^f^	1,4-dioxane	80 °C/24 h	5:0.1:0.5:0	78
12	5 mol %	H_2_O_2_·urea:H_2_O^f^	1,4-dioxane	90 °C/24 h	4:0.1:1:0	64
13	5 mol %	H_2_O_2_·urea:H_2_O^f^	1,4-dioxane	60 °C/24 h	10:0:0.1:0	82
14	5 mol %	H_2_O_2_·urea:H_2_O^f^	1,4-dioxane	60 °C/72 h	10:0:0.1:0	93
15	2 mol %	H_2_O_2_·urea:H_2_O^f^	1,4-dioxane	60 °C/72 h	8:0:0.1:0	84
16	1 mol %	H_2_O_2_·urea:H_2_O^f^	1,4-dioxane	60 °C/72 h	6:0:0.1:0	64

a Isolated yields.

b Under an argon atmosphere, TsNHOH (1 equiv) in 1,4-dioxane (0.3M) were mixed with H_2_O_2_·urea (1.1 equiv), H_2_O_2_·H_2_O 30% (1.1 equiv), and MoO_2_Dipic­(HMPA) (5 mol %). The mixture was stirred at rt for 5 min, and then styrene **2** (1 equiv) is added and the reaction mixture was stirred at 60 °C for 72 h. Crude was filtered through a 1:1 Celite/silica gel pad and then purified by silica gel flash chromatography to provide nitrosulfone **3**.

c H_2_O_2_ 30% in H_2_O.

d H_2_O_2_ 35% in urea.

e 2:1 mixture of H_2_O_2_·urea and H_2_O_2_·H_2_O for a total of 2.2 equiv.

f 1:1 mixture of H_2_O_2_·urea and H_2_O_2_·H_2_O for a total of 2.2 equiv.

g 1 M.

h Vinyl sulfone **7** was only found on entry 1 conditions, then not reported for other entries.

Based on the optimized results for the nitrosulfonation of 4-*tert*-butylstyrene, we embarked on addressing the generality of the process across substituted styrenes with electron-donating-groups (EDGs) and electron-withdrawing-groups (EWGs) as means to activate or deactivate the reaction pathway (Table 2). 4-Methylstyrene worked in a similar yield and at a comparable observable rate (91%, 64h, entry 2). Moreover, 3-methyl and 2-methyl provided α-nitrosulfone **3** in similar yields (entries 3 and 4). Regular styrene worked very well, as well as 4-MeO, 3-MeO-, and 4-t-BuO-styrenes (entries 5–8). 4-Alkoxystyrenes arrived to completion at significantly faster rates (∼32 h), while 2-MeO-styrene completely failed to provide **3**. Further complexity showed that 2,4-dimethyl-, 2,5-dimethyl-, and 4-Ph-styrenes had good yields without significant rate acceleration (entries 9–11). 4-Halostyrenes also reacted in good yields with slightly slower visible rates (∼80h), while 4-I provided **3** in considerably lower yields and as a much more complex mixture (entries 12–19).

**Table 2:**
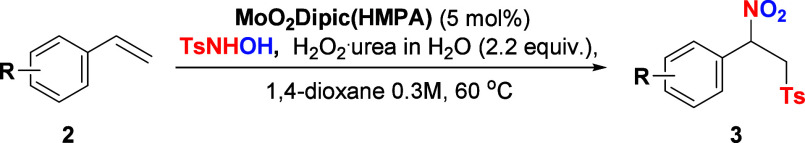

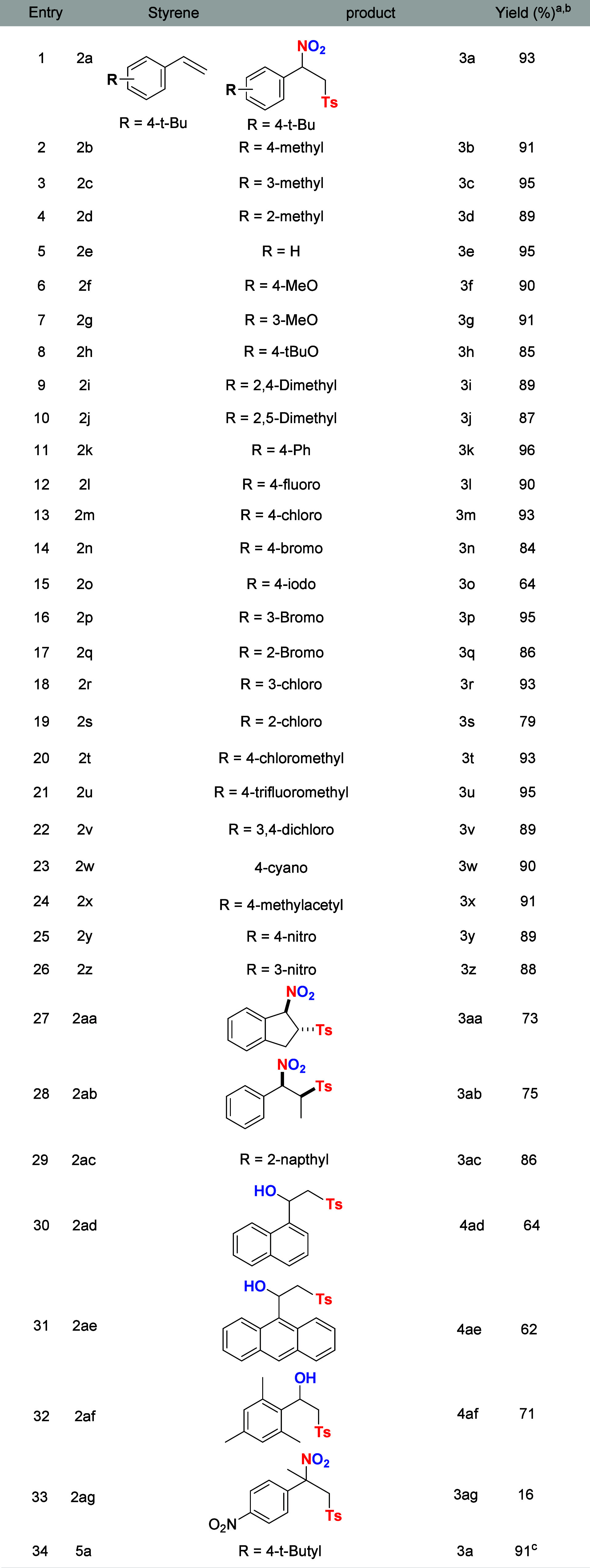
Reaction Scope

a Isolated yields.

b Under an argon atmosphere, TsNHOH (1 equiv) in 1,4-dioxane (0.3 M) were mixed with H_2_O_2_·urea (1.1 equiv), H_2_O_2_·H_2_O 30% (1.1 equiv), and MoO_2_Dipic­(HMPA) (5 mol %). The mixture was stirred at rt for 5 min, and then styrene **2** (1 equiv) was added and the reaction mixture was stirred at 60 °C for 72 h. Crude was filtered through a 1:1 Celite/silica gel pad and then purified by silica gel flash chromatography to provide nitrosulfone **3**.

c In 1,4-dioxane (0.3M), H_2_O_2_·H_2_O 30% (1.1 equiv), MoO_2_Dipic­(HMPA) (5 mol %), and oxime **5a** (1 equiv) were mixed while the reaction mixture stirred at 80 °C.

Moreover, 4-chloromethyl, 4-CF_3_, and 3,4-dichloro all reacted in good yields and comparable rates (entries 20–22). Interestingly, 2,5-dichlorostyrene reacted with full conversion but provided a complex mixture of difunctionalized products. We then studied 4-EWG-styrenes and found that 4-CN and 4-MeAc reacted in good yields and comparable observable rates, while 4-NO_2_ and 3-NO_2_ also provided α-nitrosulfone **3** in good yields with surprisingly comparable rates (entries 23–26). We wanted to address the diastereoselectivity of the reaction, and we found that indene and β-methyl-styrene both reacted in lower yields and provided **3** as mixtures of diastereomers (**3aa** in 12:1 d.r., and **3ab** in 6:1 d.r., entries 27 and 28). These results are in agreement with initial oxime formation, followed by oxidation to α-nitrosulfone. 2-Vinyl- and 1-vinyl-naphthalene provided interesting clues for this reaction; while 2-vinyl provided **3** in good yields, 1-vinyl provided hydroxysulfone **4** as the single product in 64% yield (entries 29 and 30). This result reinforces the ortho-substituent steric effect observed with other substrates and, more importantly, provides validation that ATRA forms a molybdooxaziridine radical that struggles to add on sterically demanding positions. This trend was further verified with vinyl-anthracene and 2,4,6-trimethylstyrene, where we isolated hydroxysulfones **4** as the only products (entries 31 and 32). While α-alkylstyrenes failed to provide **3**, we were able to react 4-nitro-α-methylstyrene and obtain **3ag** in 16% yield. Acrylates, conjugated carbonyls, and dienes were also tested, and complex mixtures were obtained under the reaction conditions. Product **3** was not obtained for any of these substrates.

The foundational knowledge discovered in metallooxaziridine catalysis for the difunctionalization of alkenes has helped design mechanistic studies for this new transformation. As a tandem catalysis process, we addressed the mechanism as two different cycles (Figure 1).

**Figure 1:**
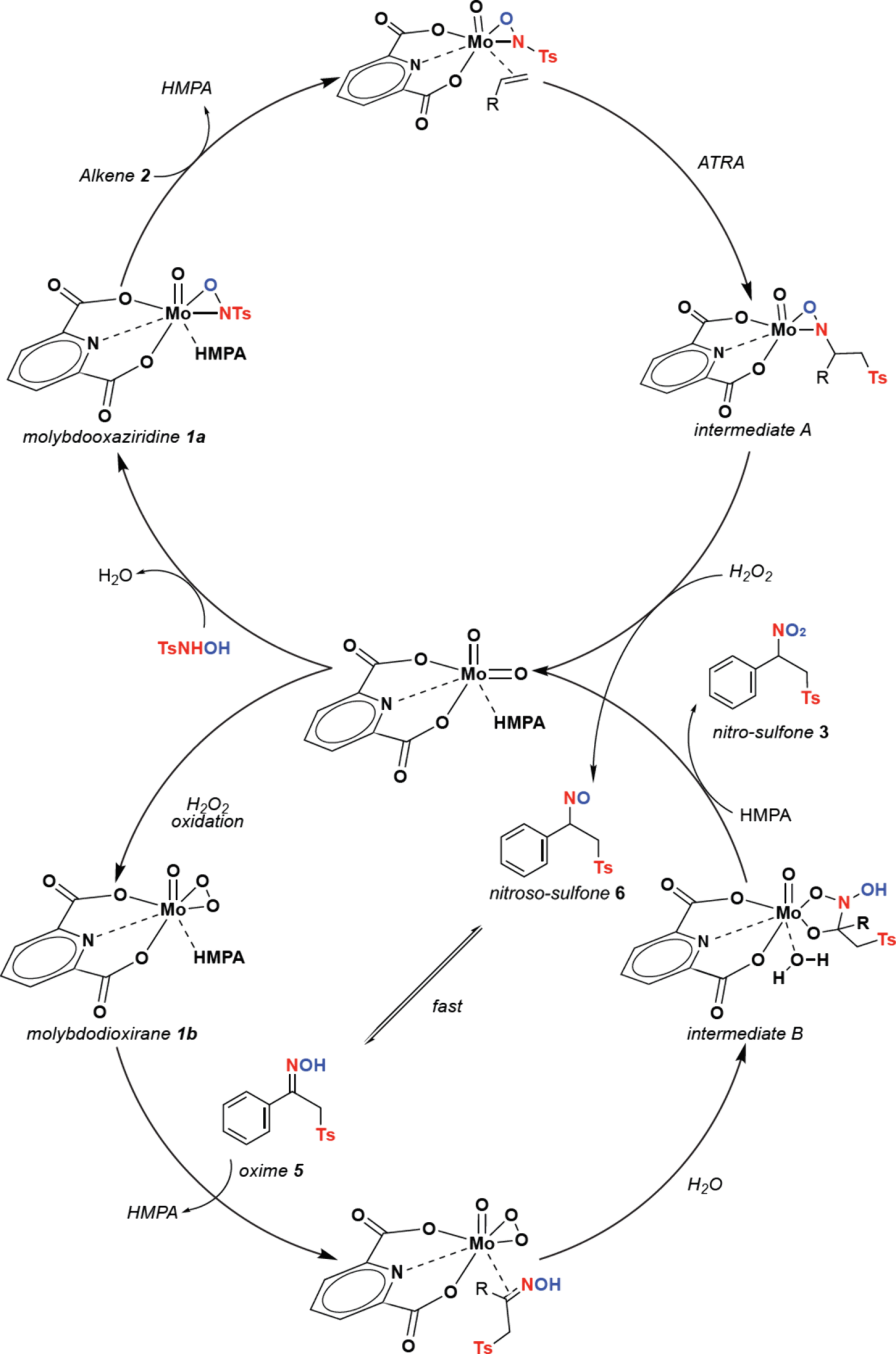
Proposed tandem catalytic cycles.

Based on the observed chemo- and regioselectivity, upon formation of **1a** and fast ligand exchange, ATRA provides intermediate **A** after fast homolytic cleavage of the N–Ts bond. Then, rate-determining-step H_2_O_2_-mediated extrusion leads to nitrososulfone **6** and MoO_2_Dipic­(HMPA), followed by fast isomerization to oxime **5** and H_2_O_2_-mediated oxidation to **1b**. Fast ligand exchange and addition of molybdodioxirane across oxime provide intermediate **B**, followed by rate-determining-step hydrolytic extrusion to provide α-nitrosulfone **3**. Control experiments showed that MoO_2_Dipic­(HMPA) is crucial for reaction conversion, and that oxime **5a** undergoes oxidation to **3a** under reaction conditions and with molybdodioxirane **1b** in H_2_O_2_ in H_2_O and 1,4-dioxane with good conversions (entry 34, Table 2).

Further analysis of reaction kinetics by deuterium-labeling competition studies revealed that ATRA is irreversible with a secondary kinetic isotope effect (KIE, **2** *e* **/2e1-** *d*_**2**_ *k*_H_ */k*_D_ = 1.03, details in SI) for cycle 1. The lack of a secondary KIE indicates a fast ATRA and supports extrusion as the rate-determining step for cycle 1. Moreover, a secondary kinetic solvent isotope effect obtained through pseudo-first-order kinetics for cycle 2 (formation of **3** from **5**) was also found (KSIE, *K*_H2O_/*K*_D2O_ = 0.84, details in SI). Similarly, this provides verification that hydrolytic extrusion is the rate-determining step for the formation of **3**. The proposed tandem catalytic cycles are in further agreement with a Hammett correlation study employing 4-substituted-substrates for both cycles. Those results show a ρ-value of – 1.28 for the formation of **5** and a ρ-value of 0.42 for the formation of **3** (Figure 2). This demonstrates enhanced reactivity for styrenes with EDGs in the formation of **5** but a lesser effect for EWGs for the formation of **3** (details on SI). These results are also in agreement with the proposed rate-determining steps for each cycle.

**Figure 2:**
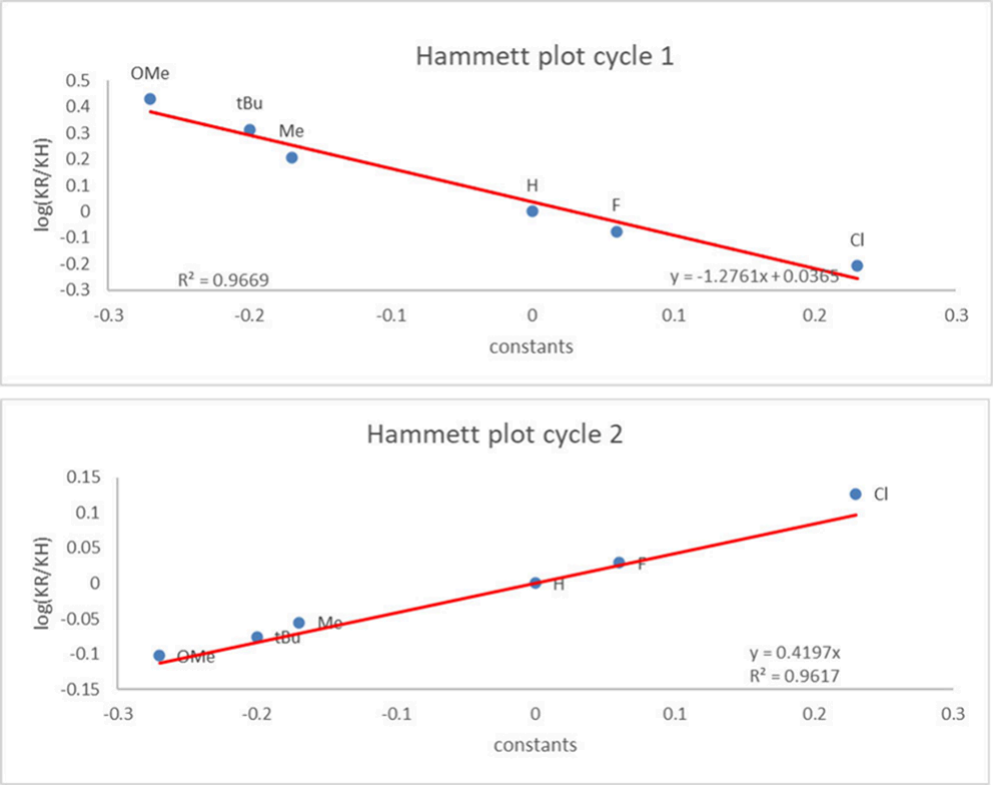
Hammett plots for both cycles.

## Conclusion

Our efforts have led to the discovery of a molybdooxaziridine/molybdodioxirane-mediated tandem catalytic α-nitrosulfonation of styrenes. The reaction works with high efficiency and stereoselectivity for alkenes with diverse substitution patterns and for styrenes with a variety of functional groups. The proposed catalytic cycle involves the formation of active catalyst **1a** that then forms intermediate **A**, followed by rate-determining-step extrusion to provide nitrososulfone **6**. The second cycle starts with fast isomerization to **5**, followed by the formation of **1b**, the formation of intermediate **B**, and rate-determining-step hydrolytic extrusion to provide **3**. Efforts to identify chiral ligands to achieve an enantioselective method are ongoing, and a follow up manuscript is in preparation.

## Safety Statement

The GHS Category 1 corrosive hydrogen peroxide and category 1B carcinogenic hexamethyl phosphoramide (HMPA) constitute significant safety hazards and must be handled with extreme care.

## Supplementary Material



## Data Availability

The data underlying this study are available in the published article and its Supporting Information.

## Notes

Experimental protocols and spectroscopic data for each nitrosulfone (PDF)
