# Supplementary material for: Molybdooxaziridine–Molybdodioxirane Tandem Catalysis: Synthesis of α‑Nitrosulfones from Styrenes
Source: J Org Chem. 2026 Apr 21;91(17):5969–75. doi: 10.1021/acs.joc.6c00262 (PMC13140151; doi:10.1021/acs.joc.6c00262)
Supplement: Supplementary file 1 [file jo6c00262_si_001.pdf]

Molybdooxaziridine-Molybdodioxirane Tandem Catalysis: Synthesis of α-Nitrosulfones from Styrenes

Jenna M. Doran, Alyssa N. Singer, Steven J. Finneran, and Gustavo Moura-Letts*

moura-letts@rowan.edu
Department of Chemistry and Biochemistry, College of Science and Mathematics
Rowan University
201 Mullica Hill Rd, Glassboro, NJ, 08028

Supporting Information

A. Materials and Methods S1
B. Mechanistic experiments S2
C. Synthesis of α-nitrosulfones from **Table 2** S10
D. ^{1}H-NMR and ^{13}C-MR spectra S23

A. Materials and Methods

Reagents were obtained from Aldrich Chemical (www.sigma-aldrich.com), Acros Organics (www.us.vwr.com) or Alfa Aesar (www.us.vwr.com) and used without further purification. Solvents were obtained from EMD Miliphore DrySol (www.us.vwr.com) and degassed with N_2. Solution phase reactions were performed in glass vials or round bottom flasks with inert atmosphere and magnetic stirring. Cold baths were generated as follows: 0 °C, wet ice/water; –10 °C, ice/acetone; –20 °C, dry ice/isopropanol monitored with a thermometer; –44 °C, dry ice/CH_3CN; –63 °C, dry ice/chloroform; –78 °C, dry ice/acetone; –100 °C, liquid nitrogen in hexanes/Et_2O. Heated reactions were performed using IKA heating blocks. TLC was performed on 0.25 mm E. Merck silica gel 60 F254 plates and visualized under UV light and/or the following stain solutions: cerium ammonium molybdate (CAM), phosphomolybdic acid (PMA), iodine (I_2), or *p*-anisaldehyde. Silica flash chromatography was performed on E. Merck 230–400 mesh silica gel 60. Automated chromatography was performed on a ISOLERA Prime instrument with 10 g. SNAP silica gel normal phase cartridges using a flow rate of 12.0 mL/min and a gradient of 0–100% EtOAc in heptanes over 20 min with UV detection at 254 nm. NMR spectra were recorded on a Bruker Avance Neo 400 MHz Spectrometer at 24 °C in $CDCl_3$ unless otherwise indicated. Chemical shifts are expressed in ppm relative to TMS (^1H, 0 ppm) or solvent signals: $CDCl_3$ (^1H, 7.23 ppm; ^{13}C, 77.0 ppm); coupling constants are expressed in Hz. Low and high resolution mass spectroscopy was performed on a Agilent 6230 Accurate-Mass Time-of-Flight 1290 Infinity UHPLC/MS. Elemental analysis was performed on a Perkin Elmer CHN 2400 analyzer.

General method for the synthesis of α-nitrosulfone 3:

In a 16 mL vial packed with a magnetic stirrer, MoO_2Dipic(HMPA) (0.05 mmol, 5 mol%), TsNHOH (1.0 mmol, 1.0 equiv.), H_2O_2 in urea (1.1 mmol, 1.1 equiv.), and H_2O_2 in H_2O 30% (1.1 mmol, 1.1 equiv.) were mixed in 1,4-dioxane (0.3M) under argon and stirred at rt for 5 min. The reaction turns light yellow, then alkene **2** (1 mmol, 1 equiv.) was added via syringe and the resulting mixture heated to 60 ºC (using heating blocks) and allowed to stir for 72h or until disappearance of alkene by TLC. The crude was then filtered by a 1:1 silica gel/celite pad and the resulting crude was then purified by silica gel chromatography to provide the corresponding α-nitrosulfone **3**.

Mechanism and kinetic experiments:

Given the tandem nature of this catalytic process, we will study the kinetics of cycle 1 and cycle 2 independently. To address the kinetics for cycle 1 (formation of oxime **5**), we employed reaction conditions to minimize formation of α-nitrosulfone **3** (Table 1, entry 2 in paper). Based on our previous efforts on studying the reaction kinetics of molybooxaziridine catalysis. We anticipate TsNHOH having saturation kinetics, thus MoO_2Dipic(HMPA) would undergo fast reaction with TsNHOH to form complex **1a** (yellow in solution) prior to alkene coordination. At the reaction temperature, ligand-exchange triggers MoO_2NTsDipic(alkene) atom transfer radical addition across the substrate to provide intermediate **A**. Then slow H_2O_2-promoted extrusion allows formation of nitrososulfone **6** and fast isomerization to oxime **5**. If peroxide-promoted MoO_2Dipic(HMPA) extrusion is the rate-determining-step, the rate constants for ATRA and isomerization steps must be much larger than the rate constant for the extrusion step. Thus, we anticipate pseudo-first-order kinetics for this reaction. We tested pseudo-first order kinetics for styrene and four other para-substituted styrenes (Figure S1).

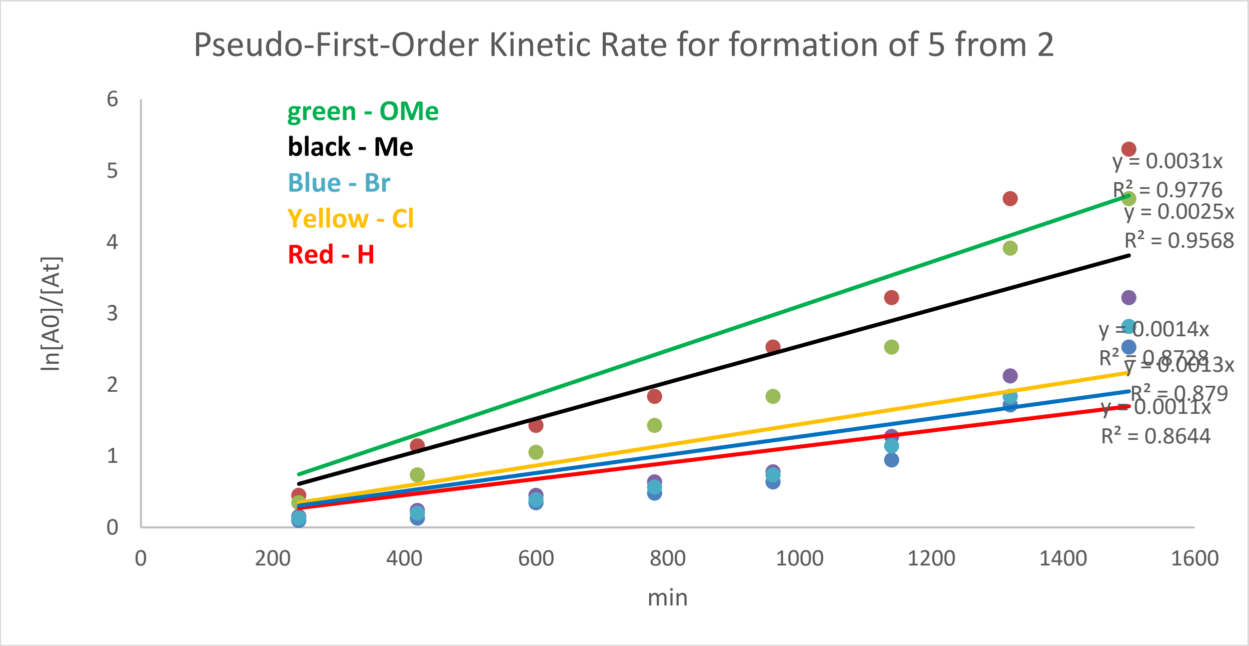

Figure S1. Pseudo-first order kinetic rate plots for cycle 1.

Reaction rates in kinetic experiments were monitored every 4 hours up to full conversion to oximes 5b, 5e, 5f, 5m, and 5n. The observed pseudo-first order kinetic plots for para-substituted styrenes agree with fast alkene/HMPA exchange, slow rate-determining-step H_2O_2-promoted extrusion, and fast ATRA and isomerization steps. It is important to mention that under catalytic conditions and high heat, nitrosoalkane **6** can undergo direct oxidation to α-nitrosulfone **3**, however isomerization to oxime **5** is reported to be considerably faster.

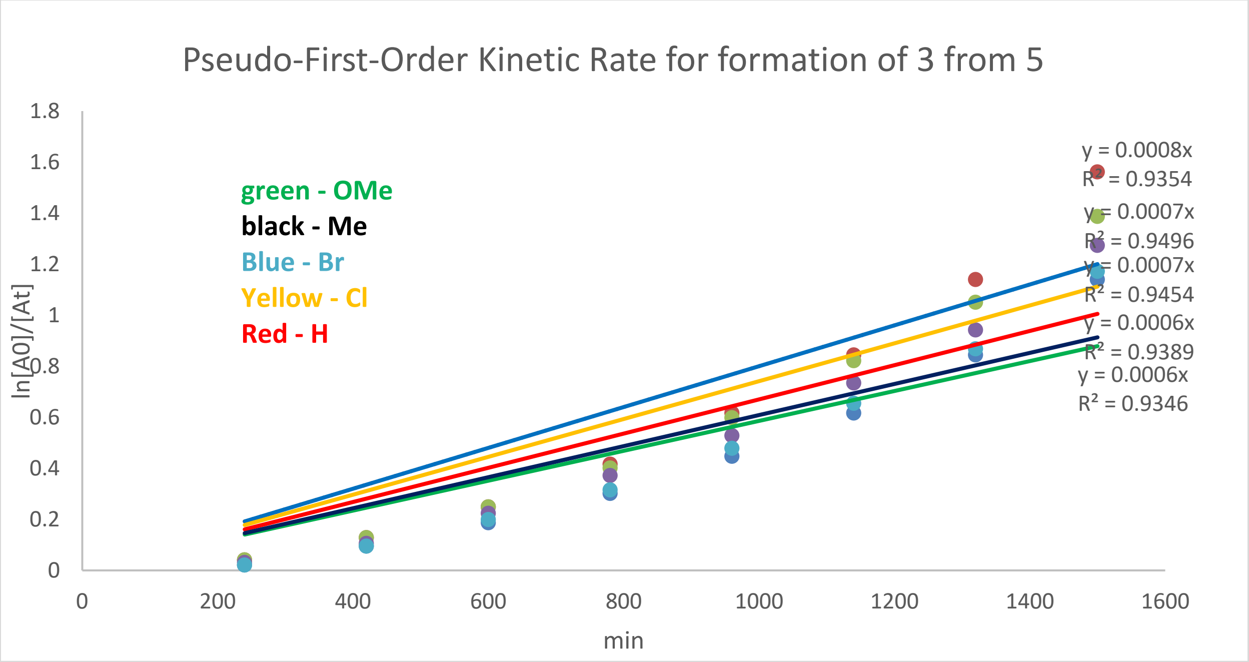

Figure S2. Pseudo-first order kinetic rate plots for cycle 2.

To address the kinetics for cycle 2 (formation of **3** from **5**), we employed the optimized reaction conditions (Table 1, entry 13 in paper). Based on our previous efforts on studying the reaction

kinetics of molybooxaziridine catalysis, MoO2Dipic(HMPA) can undergo competitive oxidation to form molybdodioxirane **1b**. At the reaction temperature, ligand-exchange with oxime **5** is followed by molybdodioxirane addition across oxime to form intermediate **B**, then followed by slow hydrolytic extrusion to form α-nitrosulfone **3**. If the hydrolytic step is the rate-determining-step, the rate constants for molybdodioxirane addition to form intermediate **B** must be much larger than the rate constant for the hydrolytic step. Thus, we anticipate pseudo-first-order kinetics for this reaction. We tested pseudo-first order kinetics for oxime **5e** and four other para-substituted phenyl oximes (**Figure S2**). Reaction rates in kinetic experiments were monitored every four hours up to full conversion to α-nitrosulfones 3b, 3e, 3f, 3m, and 3n. The observed pseudo-first order kinetic plots for para-substituted phenyloximes agree with fast addition and slow, rate-determining-step, hydrolytic extrusion.

General procedure for pseudo-first-order kinetic plots (cycle 1): In a 16 mL vial packed with a magnetic stirrer, MoO2Dipic(HMPA) (0.05 mmol, 5 mol%), TsNHOH (1.0 mmol, 1.0 equiv.), and H_2O_2 in urea 30% (2.2 mmol, 2.2 equiv.) were mixed in CH_3CN (0.3M) under argon and stirred at rt for 5 min. The reaction turns light yellow, then alkene **2e** (3.3 mmol, 3 equiv.) was added via syringe and the resulting mixture heated to 80 ºC. Aliquots (200 μL) were removed periodically over a period of 48 hours, filtered by a 1:1 silica gel/celite pad, washed with EtOAc and the resulting mixture was then analyzed by ^{1}H-NMR to quantify the formation of oxime **5e**.

General procedure for pseudo-first-order kinetic plots (cycle 2): In a 16 mL vial packed with a magnetic stirrer, MoO2Dipic(HMPA) (0.05 mmol, 5 mol%), TsNHOH (1.0 mmol, 1.0 equiv.), H_2O_2 in urea (1.1 mmol, 1.1 equiv.), and H_2O_2 in H_2O 30% (1.1 mmol, 1.1 equiv.) were mixed in 1,4-dioxane (0.3M) under argon and stirred at rt for 5 min. The reaction turns light yellow, then oxime **5e** (3.3 mmol, 3 equiv.) was added via syringe and the resulting mixture heated to 60 ºC. Aliquots (200 μL) were removed periodically over a period of 48 hours, filtered by a 1:1 silica gel/celite pad, washed with EtOAc and the resulting mixture was then analyzed by ^{1}H-NMR to quantify the formation of α-nitrosulfone **3e**.

Competition experiments: These experiments were designed using deuterated β-*d* styrene 2e1-d_2 to address the nature of the proposed catalytic cycles. A competition experiment between 2e versus 2e1-d_2 would provide secondary kinetic isotope effect data. Thus, a mixture (1:1, 4 equiv. each) of 2e/2e1-d_2 was reacted under general method to provide the respective oxime *5e2-d2*. After correcting for 2e1-d_2 deuterium purity (96%-*d*), we obtained k_H/k_D of 1.03. The lack of secondary kinetic isotope effect confirms that ATRA with molybdooxaziridine across the alkene addition is rather fast. This is also in agreement with a highly concerted intermediate **A** formation step, thus supporting the extrusion step as the rate-determining-step.

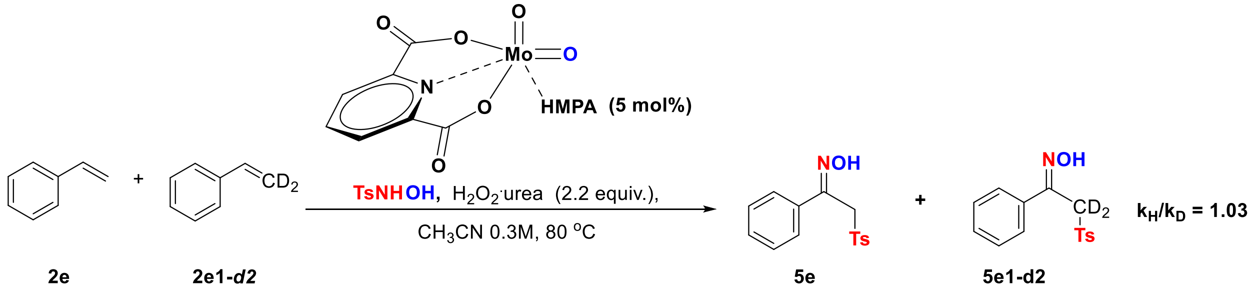

General Procedure for competition experiments with deuterated-alkenes: In a 16 mL vial packed with a magnetic stirrer, MoO2Dipic(HMPA) (0.05 mmol, 5 mol%), TsNHOH (1.0 mmol, 1.0 equiv.), H_2O_2 in urea (2.2 mmol, 2.2 equiv.) were mixed in CH_3CN (0.3M) under argon and stirred at rt for 5 min. The reaction turns light yellow, then and alkene **2e** (0.25 mmol, 0.5 equiv.), and alkene **2e1-d₂** (0.25 mmol, 0.5 equiv.) were added via syringe and the resulting mixture heated to 80 ºC. The reaction was then quickly flashed with argon and allowed to stir at rt for 72 hours. The crude was then filtered by a 1:1 silica gel/celite pad and the resulting mixture was then purified

by silica gel chromatography and the ratio of oximes **5e** and **5e1-d$_2$** was calculated from the resulting ^{1}H-NMR spectra.

Kinetic solvent isotope effect (KSIE) experiments were also performed using **5e** in 1,4-dioxane/ H_2O_2·urea/H_2O and 1,4-dioxane/ H_2O_2·urea/D_2O. Pseudo-first order kinetic plots were determined for catalytic cycle 2 and the rate constants measured. The results indicate a clear rate acceleration from H_2O to D_2O, with a K_{H2O}/K_{D2O} = 0.84 for cycle 2 The inverse KSIEs corroborates that the hydrolytic step in cycle 2 is the rate-determining-steps.

General procedure for KSIE under pseudo-first-order rate constants fo cycle 2: In a 16 mL vial packed with a magnetic stirrer, MoO_2Dipic(HMPA) (0.05 mmol, 5 mol%), TsNHOH (1.0 mmol, 1.0 equiv.), H_2O_2 in urea (2.2 mmol, 2.2 equiv.), were mixed in 1,4-dioxane and D_2O 3:1 (0.3M) under argon and stirred at rt for 5 min. The reaction turns light yellow, then oxime **5e** (3.3 mmol, 3 equiv.) was added via syringe and the resulting mixture heated to 60 °C. Aliquots (200 µL) were removed periodically over a period of 48 hours, filtered by a 1:1 silica gel/celite pad, washed with EtOAc and the resulting mixture was then analyzed by ^{1}H-NMR to quantify the formation of α-nitrosulfone **3e**.

Hammett experiments:

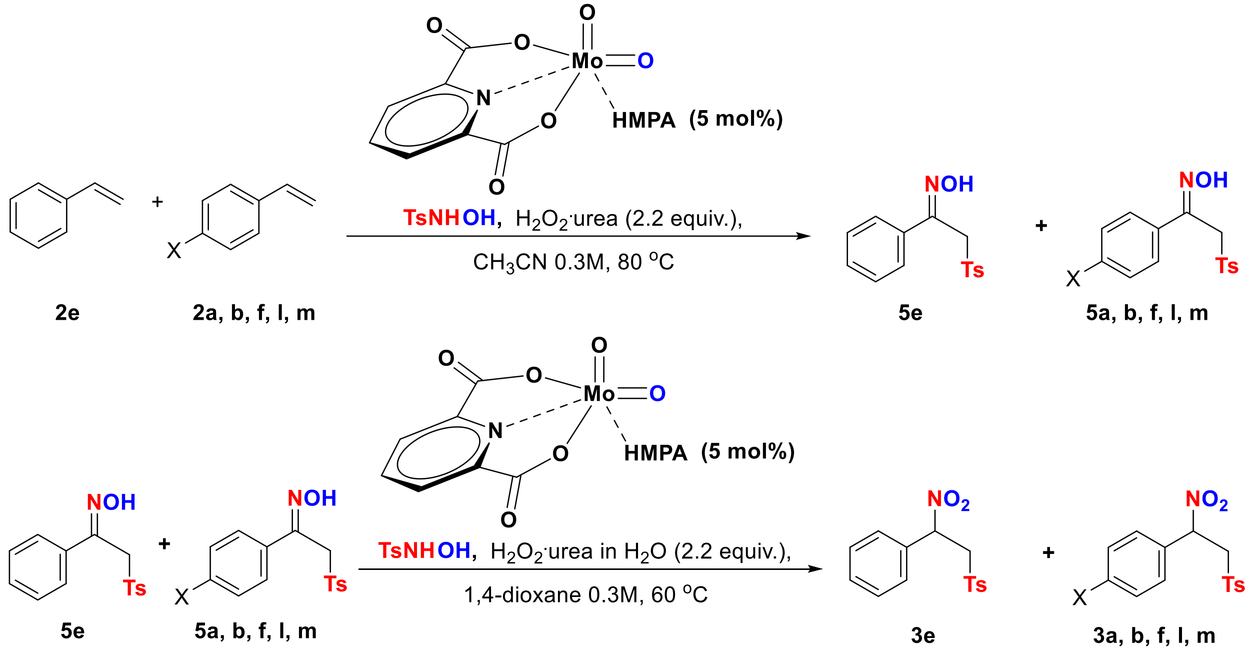

General Procedure for competition experiments for Hammett plot in cycle 1: In a 16 mL vial packed with a magnetic stirrer, MoO_2Dipic(HMPA) (0.05 mmol, 5 mol%), TsNHOH (1.0 mmol, 1.0 equiv.), and H_2O_2 in urea 30% (2.2 mmol, 2.2 equiv.) were mixed in CH_3CN (0.3M) under argon and stirred at rt for 5 min. The reaction turns light yellow, alkene **2e** (1 mmol, 2 equiv.), and alkene **2a** (1 mmol, 2 equiv.) were added via syringe and the resulting mixture heated to 80 °C and allowed to stir for 72h. The crude was then filtered by a 1:1 silica gel/celite pad and the resulting mixture was then purified by silica gel chromatography and the ratio of oximes **5e** and **5a** was calculated from the resulting ^{1}H-NMR spectra. The same procedure was performed separately for alkenes **2b**, **2f**, **2l**, and **2m**.

Entry	X	C_1 K_R/K_H	$Log(K_R/K_H)$	constant	C_2 K_R/K_H	$Log(K_R/K_H)$
1	H	1	0	0	1	0
2	Me	1.61	0.206826	-0.17	0.88	-0.05552
3	t-Bu	2.06	0.313867	-0.20	0.84	-0.07572
4	MeO	2.68	0.428135	-0.27	0.79	-0.10237
5	Cl	0.84	-0.20761	0.23	1.34	0.12711
6	F	0.62	-0.07572	0.06	1.07	0.02938

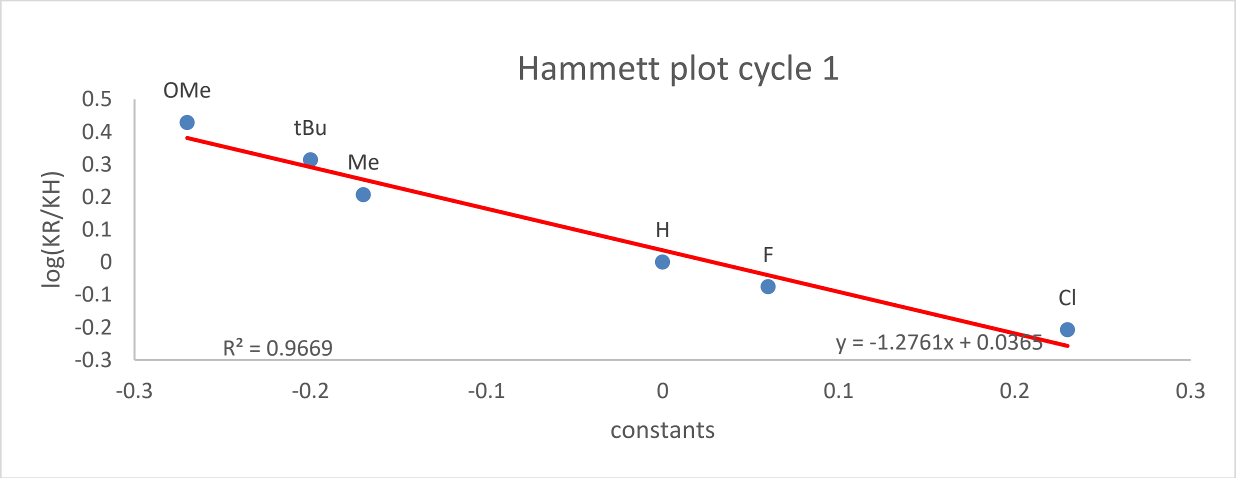

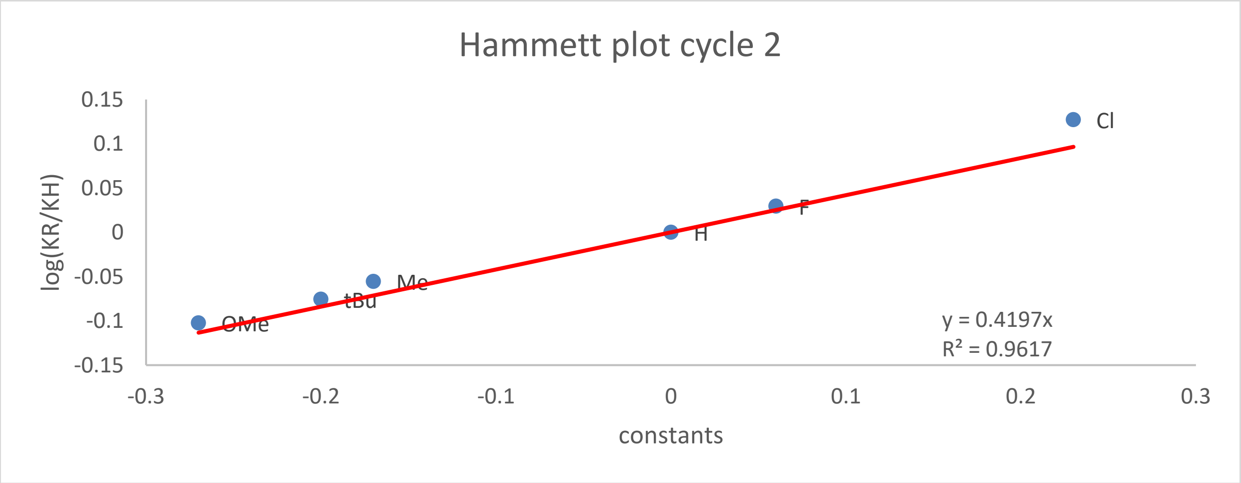

Figure S3. Hammet plots for both cycles.

General Procedure for competition experiments for Hammett plot in cycle 2: In a 16 mL vial packed with a magnetic stirrer, MoO_2Dipic(HMPA) (0.05 mmol, 5 mol%), TsNHOH (1.0 mmol, 1.0 equiv.), H_2O_2 in urea (1.1 mmol, 1.1 equiv.), and H_2O_2 in H_2O 30% (1.1 mmol, 1.1 equiv.) were mixed in 1,4-dioxane (0.3M) under argon and stirred at rt for 5 min. The reaction turns light yellow, oxime **5e** (1 mmol, 2 equiv.), and oxime **5a** (1 mmol, 2 equiv.) were added via syringe and the resulting mixture heated to 60 °C and allowed to stir for 72h. The crude was then filtered by a 1:1 silica gel/celite pad and the resulting mixture was then purified by silica gel chromatography and the ratio of α-nitrosulfones **3e** and **3a** was calculated from the resulting ^{1}H-NMR spectra. The same procedure was performed separately for oximes **5b**, **5f**, **5l**, and **5m**.

The Hammett experiments provided a ρ = -1.28 for cycle 1 and 0.42 for cycle 2, indicating that EDGs enhance the reaction rate in cycle 1 but EWGs have a lesser effect on the reaction rate in reaction 2. These results agree with a fast and highly concerted ATRA step for the formation of intermediate **A**, and a fast molybdodioxirane addition to form intermediate **B**. The observed acceleration with EDGs in the extrusion rate-determining-step for cycle 1 can be rationalized due to the development of a positive charge on the *N* in **TS-A**. On the other hand, the observed positive but small ρ with EWGs in the hydrolytic rate-determining-step for cycle 2 can be rationalized as a small negative charge develops on the *O* in **TS-B**. In **TS-B** the H_2O low-field ligand does not engage in proton transfer, thus allowing for another H_2O molecule to trigger the proton transfer (**Figure S4**).

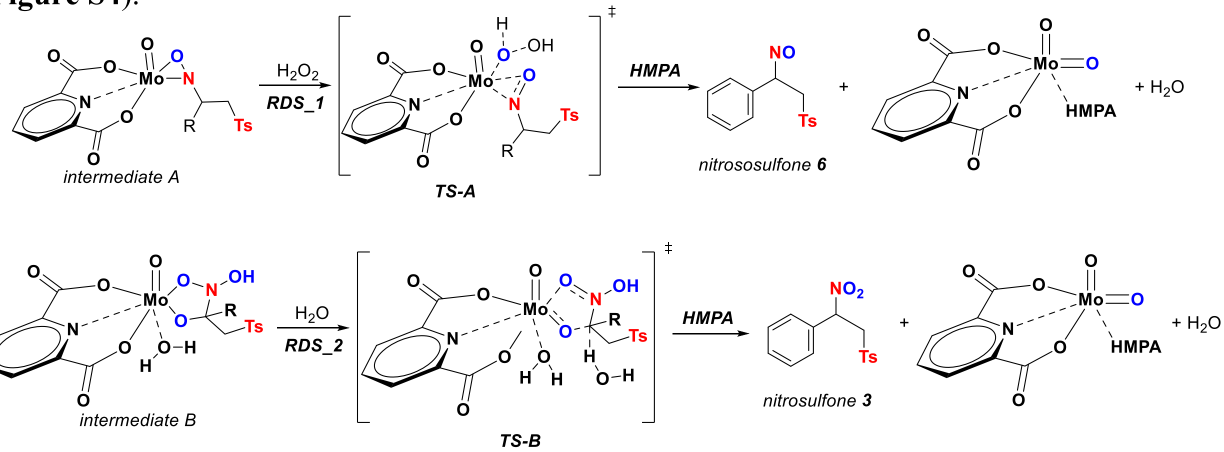

Figure S4. Proposed transition states for each catalytic cycle rate determining steps.

Control experiments: Experiments in the absence of MoO_2Dipic(HMPA) did not provide any product **4**, **5**, **6** or **3**. The full reaction in the presence of TEMPO and BHT did not provide any observable product **4**, **5**, **6** or **3**.

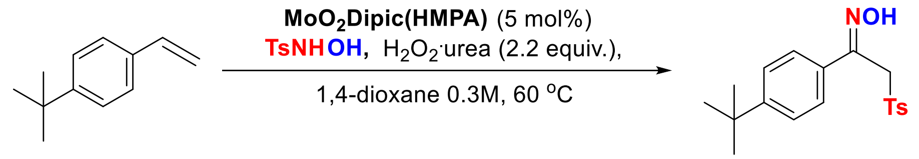

oxime **5a**, 48 % yield

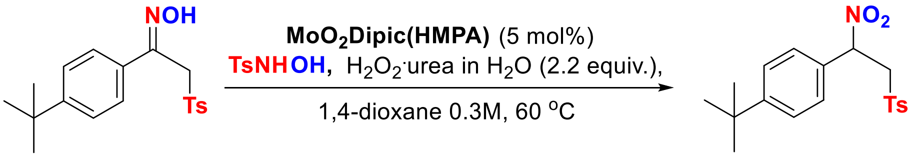

nitrosulfone **3a**, 71 % yield

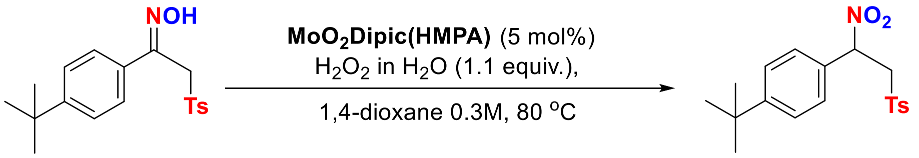

nitrosulfone **3a**, 91 % yield

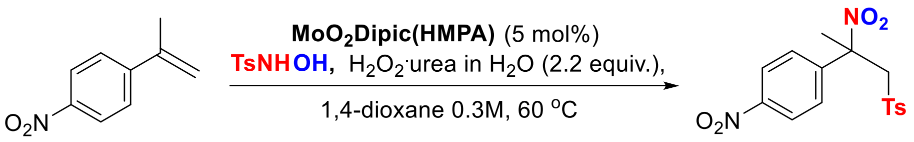

nitrosulfone, 16 % yield

We studied the formation of oxime **5** and the reaction of oxime **5** to α-nitrosulfone **3** separately, and without further optimization the respective products were obtained in moderate yields. Additionally, we tested 4-nitro-α-methylstyrene and we were able to isolate the respective α-nitrosulfone. This result is quite significant because oxime **5** cannot be formed during this process, thus nitrososulfone **6** undergoes direct oxidation to the observed α-nitrosulfone. Moreover, the results highlight the intrinsic electronic effect of the *p*-NO_2 on the ATRA step, thus allowing the formation of intermediate A despite the otherwise dominant steric effect.

Substrates that failed to react in comparable yields: Alkyl alkenes, conjugated alkenes, and dienes did not proceed to produce any of the observed products. The reaction pathway either led to highly complex mixtures or to completely different reaction pathways.

B. SYNTHESIS OF MOLYBDOOXAZIRIDINE 1:

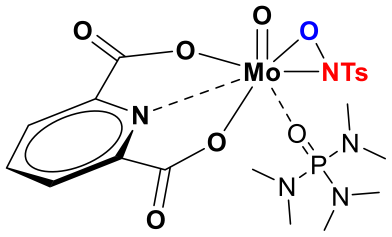

N-tosyl-Dipic(HMPA)Molybdooxaziridine (1): $MoO_2(acac)_2$ (10 mmol, 1 equiv.) is mixed in CH_2Cl_2 (0.1M, 100 mL) with Dipic (10 mmol, 1 equiv.) and HMPA (10 mmol, 1 equiv.) and the resulting heterogeneous mixture allowed to react at rt for 2 hours. The filtrate was then washed with CH_2Cl_2 (100 mL) and diethyl ether (100 mL) and then dried under vacuum to obtain a crystalline residue. N-Tosylhydroxylamine (11 mmol, 1.1 equiv.) and $MgSO_4$ (12 mmol, 1.2 equiv) were dissolved in CH_2Cl_2 (0.1M, 100 mL) at rt for 30 min. The resulting crude is then dried under vacuum and then triturated with diethyl ether (100 mL) and the resulting crystalline solid if filtered and dried under vacuum to provide pure molybdooxaziridine **1** as light orange crystals (5153 g, 8 mmol, 80% yield). **MP**: 260-262°C. **^{1}H-NMR** (400 MHz, $CDCl_3$): δ 8.54 (t, *J* = 9.1 Hz), 8.36 (d, *J* = 9.1 Hz, 2H), 7.94 (d, *J* = 8.2 Hz, 2H), 7.24 (d, *J* = 8.2, 2H), 2.36 (s, 3H), 2.24 (s, 9H), 2.22 (s, 9H). **13C{1H} NMR** (100 MHz, CD_3Cl_3): δ 166.6, 146.9, 144.6, 141.7, 130.3, 127.4, 127.0, 36.5, 21.5 ppm. **ESI-MS** *m/z* (rel int): (pos) 644.1 ($[M+H]^+$, 100). **HRMS** (ESI): Calculated for: $C_{20}H_{29}MoN_5O_9PS^+$: 644.0472, found: 644.0484. Absolute difference (ppm): 1.86.

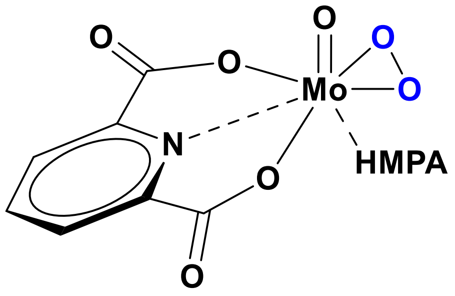

Dipic(HMPA)Molybdodioxirane (1b): $MoO_2(acac)_2$ (10 mmol, 1 equiv.) is mixed in CH_2Cl_2 (0.1M, 100 mL) with Dipic (10 mmol, 1 equiv.) and HMPA (10 mmol, 1 equiv.) and the resulting heterogeneous mixture allowed to react at rt for 24 hours. Hydrogen Peroxide 35 % in water (12 mmol, 1.2 equiv) was then added and the resulting solution stirred for 2 more hours. The solution is then filtered and dried under vacuum. The resulting crude is washed with cold diethyl ether (2 x 100 mL) to provide **1b** as light purple crystals (4.813 g, 98% yield). **MP**: 206-204°C. **^{1}H-NMR** (400 MHz, $CDCl_3$): δ 8.52 (t, *J* = 9.1 Hz), 8.37 (d, *J* = 9.1 Hz, 2H), 2.33 (s, 9H), 2.28 (s, 9H). **13C{1H} NMR** (100 MHz, CD_3Cl_3): δ 167.5, 147.9, 145.2, 128.5, 36.3 ppm. **ESI-MS** *m/z* (rel int): (pos) 491.1 ($[M+H]^+$, 100). **HRMS** (ESI): Calculated for: $C_{13}H_{22}MoN_4O_8P^+$: 491.0224, found: 491.0228. Absolute difference (ppm): 0.82.

C. SYNTHESIS OF AMINOALCOHOLS FROM TABLE 2:

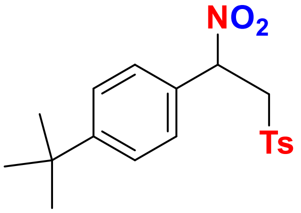

1-(tert-butyl)-4-(1-nitro-2-tosylethyl)benzene (3a): Alkene **2a** (0.1 mmol) reacted under the general method to produce α-nitrosulfone **3a** as a pale yellow oil (33.6 mg, 0.093 mmol, 93%). Purified using silica gel flash chromatography with heptanes and ethyl acetate. **TLC**: R_f : 0.48 (2:1 heptanes/EtOAc). **IR** (thin film): υ 3003, 2993, 1617, 1571, 1459, 1381, 1322, 1145, 971, 817, 785, 572 cm^{-1}. **^{1}H-NMR** (400 MHz, $CDCl_3$): δ 7.65 (d, J = 7.5 Hz, 2H), 7.28 (d, J = 7.9 Hz, 2H), 7.24 (d, J = 7.5 Hz, 2H), 7.18 (d, J = 7.9 Hz, 2H), 5.83 (dd, J = 8.8, 4.0 Hz, 1H), 4.43 (dd, J = 15.1, 8.8 Hz, 1H), 3.64 (dd, J = 15.1, 4.0 Hz, 1H), 2.37 (s, 3H), 1.21 (s, 9H). **13C{1H} NMR** (100 MHz, $CDCl_3$): δ 154.0, 145.6, 135.3, 130.2, 130.1, 128.2, 127.2, 126.3, 84.3, 58.4, 34.8, 31.1, 21.7 ppm. **ESI-MS** *m/z* (rel int): (pos) 362.1 ($[M+H]^+$, 100); (neg) 360.1 ($[M-H]^-$, 100). **HRMS** (ESI): $[M+H]^+$ Calculated for: $C_{19}H_{24}NO_4S^+$: 362.1421, found: 362.1420. Absolute difference (ppm): 0.28.

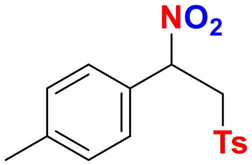

1-methyl-4-((2-nitro-2-(p-tolyl)ethyl)sulfonyl)benzene (3b): Alkene **2b** (0.1 mmol) reacted under the general method to produce α-nitrosulfone **3b** as a pale yellow oil (29.1 mg, 0.091 mmol, 91%). Purified using silica gel flash chromatography with heptanes and ethyl acetate. **TLC**: R_f : 0.38 (2:1 heptanes/EtOAc). **IR** (thin film): υ 3012, 2991, 1615, 1571, 1453, 1388, 1302, 1144, 808, 781, 572 cm^{-1}. **^{1}H-NMR** (400 MHz, $CDCl_3$): δ 7.76 (d, J = 7.5 Hz, 2H), 7.36 (d, J = 7.9 Hz, 2H), 7.28 (d, J = 7.5 Hz, 2H), 7.18 (d, J = 7.9 Hz, 2H), 5.91 (dd, J = 8.8, 4.2 Hz, 1H), 4.51 (dd, J = 15.2, 8.8 Hz, 1H), 3.69 (dd, J = 15.2, 4.2 Hz, 1H), 2.48 (s, 3H), 2.36 (s, 3H). **13C{1H} NMR** (100 MHz, $CDCl_3$): δ 145.7, 140.9, 135.3, 130.2, 130.0, 129.7, 128.2, 127.4, 84.3, 58.5, 21.7, 21.3 ppm. **ESI-MS** *m/z* (rel int): (pos) 320.1 ($[M+H]^+$, 100); (neg) 318.1 ($[M-H]^-$, 100). **HRMS** (ESI): $[M+H]^+$ Calculated for: $C_{16}H_{18}NO_4S^+$: 320.0951, found: 320.0953. Absolute difference (ppm): 0.62.

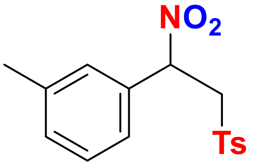

1-methyl-3-(1-nitro-2-tosylethyl)benzene (3c): Alkene **2c** (0.1 mmol) reacted under the general method to produce aminoalcohol **3c** as a light yellow oil (30.3 mg, 0.095 mmol, 95%). Purified using silica gel flash chromatography with heptanes and ethyl acetate. **TLC**: R_f: 0.38 (2:1 heptanes/EtOAc). **IR** (thin film): υ 3050, 2996, 1551, 1348, 1160, 701, 648 cm^{-1}. **^{1}H-NMR** (400 MHz, $CDCl_3$): δ 7.65 (d, J = 7.5 Hz, 2H), 7.25 (d, J = 7.9 Hz, 2H), 7.20-7.05 (m, 4H), 6.19 (dd, J = 8.7, 3.9 Hz, 1H), 4.45 (dd, J = 15.1, 8.7 Hz, 1H), 3.56 (dd, J = 15.1, 8.7 Hz, 1H), 2.40 (s,

3H), 2.37 (s, 3H). **13C{1H} NMR** (100 MHz, $CDCl_3$): δ 145.7, 136.9, 131.4, 130.3, 130.2, 130.1, 128.1, 128.0, 127.1, 125.8, 80.3, 58.6, 21.7, 19.4 ppm. **ESI-MS** *m/z* (rel int): (pos) 320.1 ($[M+H]^+$, 100); (neg) 318.1 ($[M–H]^-$, 100). **HRMS** (ESI): $[M+H]^+$ Calculated for: $C_{16}H_{18}NO_4S^+$: 320.0951, found: 320.0954. Absolute difference (ppm): 0.94.

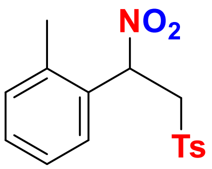

1-methyl-2-(1-nitro-2-tosylethyl)benzene (3d): Alkene **2d** (0.1 mmol) reacted under the general method to produce α-nitrosulfone **3d** as a pale yellow oil (28.4 mg, 0.089 mmol, 89%). Purified using silica gel flash chromatography with heptanes and ethyl acetate. **TLC**: R_f: 0.40 (2:1 heptanes/EtOAc). **IR** (thin film): υ 3005, 2994, 1618, 1569, 1454, 1388, 1304, 1142, 972, 811, 574 cm^{-1}. **^{1}H-NMR** (400 MHz, $CDCl_3$): δ 7.67 (d, *J* = 7.5 Hz, 2H), 7.27 (d, *J* = 7.8 Hz, 2H), 7.19-7.04 (m, 4H), 6.20 (dd, *J* = 8.7, 3.9 Hz, 1H), 4.46 (dd, *J* = 15.1, 8.7 Hz, 1H), 3.56 (dd, *J* = 15.1, 3.9 Hz, 1H), 2.40 (s, 3H), 2.38 (s, 3H). **13C{1H} NMR** (100 MHz, $CDCl_3$): δ 145.7, 136.9, 135.3, 131.4, 131.3, 130.3, 130.2, 128.1, 127.1, 125.7, 80.3, 58.6, 21.7, 19.4 ppm. **ESI-MS** *m/z* (rel int): (pos) 320.1 ($[M+H]^+$, 100); (neg) 318.1 ($[M–H]^-$, 100). **HRMS** (ESI): $[M+H]^+$ Calculated for: $C_{16}H_{18}NO_4S^+$: 320.0951, found: 320.0946. Absolute difference (ppm): 1.56.

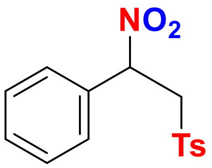

1-methyl-4-((2-nitro-2-phenylethyl)sulfonyl)benzene (3e): Alkene **2e** (0.1 mmol) reacted under the general method to produce α-nitrosulfone **3e** as a clear oil (28.9 mg, 0.095 mmol, 95%). Purified using silica gel flash chromatography with heptanes and ethyl acetate. **TLC**: R_f : 0.37 (2:1 heptanes/EtOAc). **IR** (thin film): υ 3023, 2985, 1632, 1571, 1463, 1377, 1302, 1142, 808, 763, 574 cm^{-1}. **^{1}H-NMR** (400 MHz, $CDCl_3$): δ 7.75 (d, *J* = 7.5 Hz, 2H), 7.41-7.33 (m, 7H), 5.94 (dd, *J* = 9.1, 3.8 Hz, 1H), 4.52 (dd, *J* = 15.1, 9.1 Hz, 1H), 3.69 (dd, *J* = 15.1, 3.8 Hz, 1H), 2.45 (s, 3H). **13C{1H} NMR** (100 MHz, $CDCl_3$): δ 145.8, 135.3, 132.5, 130.9, 130.2, 129.4, 128.1, 127.6, 84.5, 58.4, 21.7 ppm. **ESI-MS** *m/z* (rel int): (pos) 306.1 ($[M+H]^+$, 100); (neg) 304.1 ($[M–H]^-$, 100). **HRMS** (ESI): $[M+H]^+$ Calculated for: $C_{15}H_{16}NO_4S^+$: 306.0795, found: 306.0791. Absolute difference (ppm): 1.31.

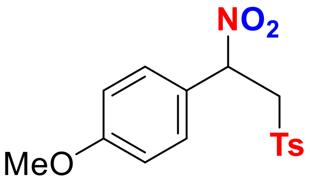

1-methoxy-4-(1-nitro-2-tosylethyl)benzene (3f): Alkene **2f** (0.1 mmol) reacted under the general method to produce α-nitrosulfone **3f** as a light yellow oil (30.2 mg, 0.090 mmol, 90%). Purified using silica gel flash chromatography with heptanes and ethyl acetate. **TLC**: R_f: 0.27 (2:1 heptanes/EtOAc). **IR** (thin film): υ 3018, 2985, 1618, 1579, 1434, 1384, 1250, 1106, 802, 563 cm^{-1}. **^{1}H-NMR** (400 MHz, $CDCl_3$): δ 7.63 (d, *J* = 7.5 Hz, 2H), 7.18 (d, *J* = 8.0 Hz, 2H), 6.75 (d, *J* = 8.7 Hz, 2H), 5.77 (dd, *J* = 8.9, 4.1 Hz, 1H), 4.38 (dd, *J* = 15.0, 8.9 Hz, 1H), 3.69 (s, 3H), 3.60 (dd, *J* = 15.0, 4.1 Hz, 1H), 2.35 (s, 3H). **13C{1H} NMR** (100 MHz, $CDCl_3$): δ 161.3, 145.7, 135.3, 130.1, 129.0, 128.2, 124.5, 114.6, 80.1, 58.4, 55.4, 21.7 ppm. **ESI-MS** *m/z* (rel int): (pos) 336.1 ($[M+H]^+$, 100); (neg) 334.1 ($[M-H]^-$, 100). **HRMS** (ESI): $[M+H]^+$ Calculated for: $C_{16}H_{18}NO_5S^+$: 336.0900, found: 336.0904. Absolute difference (ppm): 1.19.

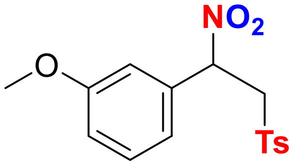

1-methoxy-3-(1-nitro-2-tosylethyl)benzene (3g): Alkene **2g** (0.1 mmol) reacted under the general method to produce α-nitrosulfone **3g** as a pale yellow oil (30.5 mg, 0.091 mmol, 91%). Purified using silica gel flash chromatography with heptanes and ethyl acetate. **TLC**: R_f: 0.28 (2:1 heptanes/EtOAc). **IR** (thin film): υ 3035, 2979, 1625, 1575, 1438, 1388, 1258, 1087, 817, 561 cm^{-1}. **^{1}H-NMR** (400 MHz, $CDCl_3$): δ 7.65 (d, *J* = 8.4 Hz, 2H), 7.25 (d, *J* = 8.0 Hz, 2H), 7.17 (d, *J* = 3.4 Hz, 1H), 6.86 – 6.79 (m, 2H), 6.74 (s, 1H), 5.80 (dd, *J* = 9.1, 3.7 Hz, 1H), 4.39 (dd, *J* = 15.1, 9.1 Hz, 1H), 3.69 (s, 3H), 3.58 (dd, *J* = 15.1, 3.7 Hz, 1H), 2.36 (s, 3H). **13C{1H} NMR** (100 MHz, $CDCl_3$): δ 160.1, 145.8, 135.3, 133.7, 130.5, 130.2, 128.2, 119.6, 116.1, 113.0, 84.5, 58.5, 55.4, 21.8 ppm. **ESI-MS** *m/z* (rel int): (pos) 336.1 ($[M+H]^+$, 100); (neg) 334.1 ($[M-H]^-$, 100). **HRMS** (ESI): $[M+H]^+$ Calculated for: $C_{16}H_{18}NO_5S^+$: 336.0900, found: 336.0903. Absolute difference (ppm): 0.89.

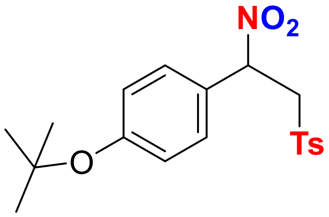

1-(tert-butoxy)-4-(1-nitro-2-tosylethyl)benzene (3h): Alkene **2h** (0.1 mmol) reacted under the general method to produce α-nitrosulfone **3h** as a clear oil (32.0 mg, 0.085 mmol, 85%). Purified using silica gel flash chromatography with heptanes and ethyl acetate. **TLC**: R_f: 0.36 (2:1 heptanes/EtOAc). **IR** (thin film): υ 3027, 2991, 1618, 1571, 1438, 1373, 1205, 1081, 811, 503 cm^{-1}. **^{1}H-NMR** (400 MHz, $CDCl_3$): δ 7.74 (d, *J* = 8.3 Hz, 1H), 7.34 (d, *J* = 8.1 Hz, 1H), 7.25 (d, *J* =

8.4 Hz, 1H), 6.94 (d, *J* = 8.6 Hz, 1H), 5.89 (dd, *J* = 9.2, 3.7 Hz, 1H), 4.50 (dd, *J* = 15.1, 9.2 Hz, 1H), 3.68 (dd, *J* = 15.1, 3.8 Hz, 1H), 2.45 (s, 3H), 1.34 (s, 9H). **13C{1H} NMR** (100 MHz, $CDCl_3$): δ 157.7, 145.7, 135.3, 130.2, 128.3, 128.2, 126.7, 124.1, 84.1, 79.4, 58.5, 28.8, 21.7 ppm. **ESI-MS** *m/z* (rel int): (pos) 378.1 ($[M+H]^+$, 100); (neg) 376.1 ($[M–H]^-$, 100). **HRMS** (ESI): $[M+H]^+$ Calculated for: $C_{19}H_{24}NO_5S^+$: 378.1370, found: 378.1374. Absolute difference (ppm): 1.06.

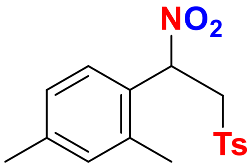

2,4-dimethyl-1-(1-nitro-2-tosylethyl)benzene (3i): Alkene **2i** (0.1 mmol) reacted under the general method to produce α-nitrosulfone **3i** as a pale yellow oil (29.6 mg, 0.089 mmol, 89%). Purified using silica gel flash chromatography with heptanes and ethyl acetate. **TLC**: R_f: 0.39 (2:1 heptanes/EtOAc). **IR** (thin film): υ 3032, 2990, 1618, 1573, 1441, 1381, 1302, 1142, 976, 817, 765, 538 cm^{-1}. **^{1}H-NMR** (400 MHz, $CDCl_3$): δ 7.73 (d, *J* = 7.5 Hz, 2H), 7.32 (d, *J* = 7.9 Hz, 2H), 7.03 (d, *J* = 7.5 Hz, 2H), 6.93 (s, 1H), 6.21 (dd, *J* = 8.6, 4.1 Hz, 1H), 4.50 (dd, *J* = 15.1, 8.6 Hz, 1H), 3.63 (dd, *J* = 15.1, 4.1 Hz, 1H), 2.44 (s, 3H), 2.43 (s, 3H), 2.28 (s, 3H). **13C{1H} NMR** (100 MHz, $CDCl_3$): δ 145.6, 140.5, 136.7, 135.4, 132.0, 130.1, 128.5, 128.1, 127.7, 125.8, 80.2, 58.6, 21.7, 21.1, 19.3 ppm. **ESI-MS** *m/z* (rel int): (pos) 334.1 ($[M+H]^+$, 100); (neg) 332.1 ($[M–H]^-$, 100). **HRMS** (ESI): $[M+H]^+$ Calculated for: $C_{17}H_{20}NO_4S^+$: 334.1108, found: 334.1106. Absolute difference (ppm): 0.60.

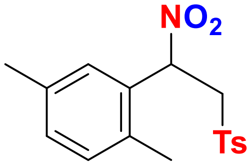

1,4-dimethyl-2-(1-nitro-2-tosylethyl)benzene (3j): Alkene **2j** (0.1 mmol) reacted under the general method to produce α-nitrosulfone **3j** as a clear oil (29.4 mg, 0.087 mmol, 87%). Purified using silica gel flash chromatography with heptanes and ethyl acetate. **TLC**: R_f: 0.39 (2:1 heptanes/EtOAc). **IR** (thin film): υ 3023, 2995, 1612, 1577, 1447, 1385, 1304, 1124, 952, 862, 701, 556 cm^{-1}. **^{1}H-NMR** (400 MHz, $CDCl_3$): δ 7.70 (d, *J* = 8.0 Hz, 2H), 7.32 (d, *J* = 7.9 Hz, 2H), 7.05 (d, *J* = 7.9 Hz, 2H), 6.91 (d, 1H), 6.23 (dd, *J* = 8.5, 4.2 Hz, 1H), 4.50 (dd, *J* = 15.1, 8.5 Hz, 1H), 3.65 (dd, *J* = 15.1, 4.3 Hz, 1H), 2.44 (s, 3H), 2.42 (s, 3H), 2.19 (s, 3H). **13C{1H} NMR** (100 MHz, $CDCl_3$): δ 145.6, 136.8, 135.4, 133.7, 131.2, 131.0, 130.9, 130.1, 128.1, 126.3, 80.3, 58.5, 21.7, 20.8, 18.8 ppm. **ESI-MS** *m/z* (rel int): (pos) 334.1 ($[M+H]^+$, 100); (neg) 332.1 ($[M–H]^-$, 100). **HRMS** (ESI): $[M+H]^+$ Calculated for: $C_{17}H_{20}NO_4S^+$: 334.1108, found: 334.1102. Absolute difference (ppm): 1.80.

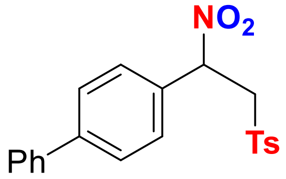

4-(1-nitro-2-tosylethyl)-1,1'-biphenyl (3k): Alkene **2k** (0.1 mmol) reacted under the general method to produce α-nitrosulfone **3k** as a pale yellow oil (36.6 mg, 0.096 mmol, 96%). Purified using silica gel flash chromatography with heptanes and ethyl acetate. **TLC**: R_f: 0.43 (2:1 heptanes/EtOAc). **IR** (thin film): υ 3023, 3005, 2994, 1609, 1571, 1407, 1377, 824, 727, 565 cm^{-1}. **^{1}H-NMR** (400 MHz, $CDCl_3$): δ 7.66 (d, *J* = 8.4 Hz, 2H), 7.49 – 7.41 (m, 4H), 7.40 – 7.29 (m, 5H), 7.25 (d, *J* = 8.0 Hz, 2H), 5.90 (dd, *J* = 8.8, 4.2 Hz, 1H), 4.45 (dd, *J* = 15.0, 8.8 Hz, 1H), 3.69 (dd, *J* = 15.0, 4.3 Hz, 1H), 2.35 (s, 3H). **13C{1H} NMR** (100 MHz, $CDCl_3$): δ 145.8, 143.7, 139.6, 135.3, 131.2, 130.2, 129.0, 128.2, 128.1, 128.0, 128.0, 127.2, 84.3, 58.5, 21.7 ppm. **ESI-MS** *m/z* (rel int): (pos) 382.1 ($[M+H]^+$, 100); (neg) 380.1 ($[M–H]^–$, 100). **HRMS** (ESI): $[M+H]^+$ Calculated for: $C_{21}H_{20}NO_4S^+$: 382.1108, found: 382.1104. Absolute difference (ppm): 1.05.

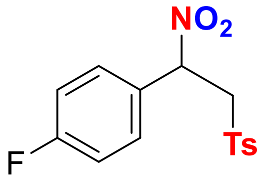

1-fluoro-4-(1-nitro-2-tosylethyl)benzene (3l): Alkene **2l** (0.1 mmol) reacted under the general method to produce α-nitrosulfone **3l** as a clear oil (29.1 mg, 0.090 mmol, 90%). Purified using silica gel flash chromatography with heptanes and ethyl acetate. **TLC**: R_f: 0.37 (2:1 heptanes/EtOAc). **IR** (thin film): υ 3005, 2985, 1618, 1568, 1435, 1364, 1308, 1124, 907, 813, 747, 581 cm^{-1}. **^{1}H-NMR** (400 MHz, $CDCl_3$): δ 7.73 (d, *J* = 8.2 Hz, 2H), 7.37 – 7.26 (m, 4H), 7.03 (t, *J* = 8.5 Hz, 2H), 5.93 (dd, *J* = 8.6, 4.4 Hz, 1H), 4.45 (dd, *J* = 15.0, 8.6 Hz, 1H), 3.69 (dd, *J* = 15.0, 4.4 Hz, 1H), 2.46 (s, 3H). **13C{1H} NMR** (100 MHz, $CDCl_3$): δ 163.9 (d, *J* = 251.6 Hz), 145.9, 135.3, 130.2, 129.8, 129.7, 128.2 (d, *J* = 2.6 Hz), 116.6 (d, *J* = 22.0 Hz), 83.8, 58.4, 21.8 ppm. **ESI-MS** *m/z* (rel int): (pos) 324.1 ($[M+H]^+$, 100); (neg) 322.1 ($[M–H]^–$, 100). **HRMS** (ESI): $[M+H]^+$ Calculated for: $C_{15}H_{15}FNO_4S^+$: 324.0700, found: 324.0705. Absolute difference (ppm): 1.54.

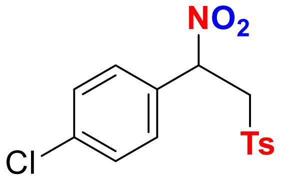

1-chloro-4-(1-nitro-2-tosylethyl)benzene (3m): Alkene **2m** (0.1 mmol) reacted under the general method to produce α-nitrosulfone **3m** as a white solid (31.5 mg, 0.093 mmol, 93%). Purified using silica gel flash chromatography with heptanes and ethyl acetate. **TLC**: R_f: 0.41 (2:1 heptanes/EtOAc). **MP**: 143-144 °C. **IR** (thin film): υ 3005, 2995, 1652, 1566, 1481, 1368, 1352, 1106, 789, 642 cm^{-1}. **^{1}H-NMR** (400 MHz, $CDCl_3$): δ 7.63 (d, *J* = 8.3 Hz, 2H), 7.28 – 7.24 (m, 4H),

7.23 – 7.17 (m, 2H), 5.83 (dd, *J* = 8.4, 4.7 Hz, 1H), 4.36 (dd, *J* = 14.9, 8.5 Hz, 1H), 3.63 (dd, *J* = 15.0, 4.7 Hz, 1H), 2.39 (s, 3H). **13C{1H} NMR** (100 MHz, $CDCl_3$): δ 145.9, 137.0, 135.2, 130.8, 130.2, 129.6, 129.0, 128.1, 83.8, 58.3, 21.8 ppm. **ESI-MS** *m/z* (rel int): (pos) 340.0 ($[M+H]^+$, 100); (neg) 338.0 ($[M-H]^-$, 100). **HRMS** (ESI): $[M+H]^+$ Calculated for: $C_{15}H_{15}ClNO_4S^+$: 340.0405, found: 340.0403. Absolute difference (ppm): 0.59.

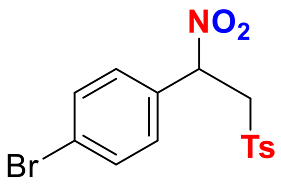

1-bromo-4-(1-nitro-2-tosylethyl)benzene (3n): Alkene **2n** (0.1 mmol) reacted under the general method to produce α-nitrosulfone **3n** as a white solid (31.5 mg, 0.084 mmol, 84%). Purified using silica gel flash chromatography with heptanes and ethyl acetate. **TLC**: R_f: 0.39 (2:1 heptanes/EtOAc). **MP**: 148-149 ºC. **IR** (thin film): υ 3014, 2997, 1618, 1571, 1438, 1348, 1291, 1142, 934, 817, 745, 620 cm^{-1}. **^{1}H-NMR** (400 MHz, $CDCl_3$): δ 7.62 (d, *J* = 8.4 Hz, 2H), 7.40 (d, *J* = 8.6 Hz, 2H), 7.24 (d, *J* = 8.2 Hz, 2H), 7.14 (d, *J* = 8.5 Hz, 2H), 5.82 (dd, *J* = 8.4, 4.8 Hz, 1H), 4.35 (dd, *J* = 15.0, 8.4 Hz, 1H), 3.64 (dd, *J* = 15.0, 4.8 Hz, 1H), 2.38 (s, 3H). **13C{1H} NMR** (100 MHz, $CDCl_3$): δ 145.9, 135.2, 132.6, 131.2, 130.2, 129.3, 128.1, 125.2, 83.9, 58.2, 21.8 ppm. **ESI-MS** *m/z* (rel int): (pos) 384.0 ($[M+H]^+$, 100); (neg) 382.0 ($[M-H]^-$, 100). **HRMS** (ESI): $[M+H]^+$ Calculated for: $C_{15}H_{15}BrNO_4S^+$: 383.9900, found: 383.9904. Absolute difference (ppm): 1.04.

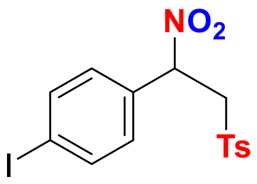

1-iodo-4-(1-nitro-2-tosylethyl)benzene (3o): Alkene **2o** (0.1 mmol) reacted under the general method to produce α-nitrosulfone **3o** as a white solid (27.6 mg, 0.064 mmol, 64%). Purified using silica gel flash chromatography with heptanes and ethyl acetate. **TLC**: R_f: 0.40 (2:1 heptanes/EtOAc). **MP**: 155-157 ºC. **IR** (thin film): υ 3054, 2995, 1610, 1553, 1438, 1340, 1219, 1106, 727, 633 cm^{-1}. **^{1}H-NMR** (400 MHz, $CDCl_3$): δ 7.70 – 7.67 (m, 4H), 7.33 (d, *J* = 8.0 Hz, 2H), 7.07 (d, *J* = 8.4 Hz, 2H), 5.86 (dd, *J* = 8.3, 4.9 Hz, 1H), 4.41 (dd, *J* = 15.0, 8.3 Hz, 1H), 3.70 (dd, *J* = 14.9, 4.9 Hz, 1H), 2.47 (s, 3H). **13C{1H} NMR** (100 MHz, $CDCl_3$): δ 145.9, 138.6, 138.1, 135.2, 131.8, 130.2, 129.3, 128.1, 84.0, 58.2, 21.8 ppm. **ESI-MS** *m/z* (rel int): (pos) 432.0 ($[M+H]^+$, 100); (neg) 430.0 ($[M-H]^-$, 100). **HRMS** (ESI): $[M+H]^+$ Calculated for: $C_{15}H_{15}INO_4S^+$: 431.9761, found: 431.9766. Absolute difference (ppm): 1.16.

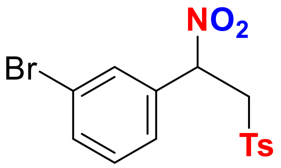

1-bromo-3-(1-nitro-2-tosylethyl)benzene (3p): Alkene **2p** (0.1 mmol) reacted under the general method to produce α-nitrosulfone **3p** as a clear oil (36.4 mg, 0.095 mmol, 95%). Purified using silica gel flash chromatography with heptanes and ethyl acetate. **TLC**: R_f: 0.39 (2:1 heptanes/EtOAc). **IR** (thin film): υ 3021, 2989, 1618, 1569, 1418, 1378, 1291, 1106, 907, 808, 664 cm^{-1}. **^{1}H-NMR** (400 MHz, $CDCl_3$): δ 7.70 (d, *J* = 8.4 Hz, 2H), 7.53 (dd, *J* = 7.8, 1.9 Hz, 1H), 7.44 (t, *J* = 1.9 Hz, 1H), 7.35 – 7.31 (m, 3H), 7.27 – 7.23 (m, 1H), 5.88 (dd, *J* = 8.5, 4.6 Hz, 1H), 4.44 (dd, *J* = 15.0, 8.4 Hz, 1H), 3.71 (dd, *J* = 15.0, 4.7 Hz, 1H), 2.45 (s, 3H). **13C{1H} NMR** (100 MHz, $CDCl_3$): δ 145.9, 135.1, 134.1, 133.8, 130.9, 130.5, 130.2, 128.1, 126.4, 123.3, 83.9, 58.2, 21.7 ppm. **ESI-MS** *m/z* (rel int): (pos) 384.0 ($[M+H]^+$, 100); (neg) 382.0 ($[M-H]^-$, 100). **HRMS** (ESI): $[M+H]^+$ Calculated for: $C_{15}H_{15}BrNO_4S^+$: 383.9900, found: 383.9903. Absolute difference (ppm): 0.78.

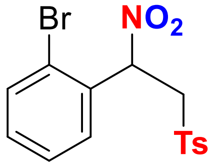

1-bromo-2-(1-nitro-2-tosylethyl)benzene (3q): Alkene **2q** (0.1 mmol) reacted under the general method to produce α-nitrosulfone **3q** as a clear oil (32.9 mg, 0.086 mmol, 86%). Purified using silica gel flash chromatography with heptanes and ethyl acetate. **TLC**: R_f: 0.33 (2:1 heptanes/EtOAc). **IR** (thin film): υ 3009, 2995, 1632, 1579, 1452, 1382, 1261, 1124, 808, 718, 684 cm^{-1}. **^{1}H-NMR** (400 MHz, $CDCl_3$): δ 7.78 (d, *J* = 8.2 Hz, 2H), 7.59 (d, *J* = 7.9 Hz, 1H), 7.35 (d, *J* = 8.0 Hz, 2H), 7.31 – 7.27 (m, 1H), 7.24 – 7.19 (m, 2H), 6.44 (dd, *J* = 8.7, 4.1 Hz, 1H), 4.46 (dd, *J* = 15.2, 8.7 Hz, 1H), 3.69 (dd, *J* = 15.2, 4.1 Hz, 1H), 2.45 (s, 3H). **13C{1H} NMR** (100 MHz, $CDCl_3$): δ 145.8, 135.1, 133.8, 132.0, 131.7, 130.2, 128.5, 128.3, 127.9, 123.9, 83.3, 58.1, 21.7 ppm. **ESI-MS** *m/z* (rel int): (pos) 384.0 ($[M+H]^+$, 100); (neg) 382.0 ($[M-H]^-$, 100). **HRMS** (ESI): $[M+H]^+$ Calculated for: $C_{15}H_{15}BrNO_4S^+$: 383.9900, found: 383.9902. Absolute difference (ppm): 0.52.

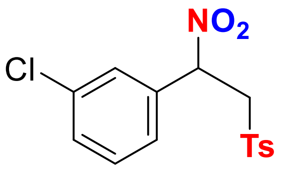

1-chloro-3-(1-nitro-2-tosylethyl)benzene (3r): Alkene **2r** (0.1 mmol) reacted under the general method to produce α-nitrosulfone **3r** as a clear oil (31.5 mg, 0.093 mmol, 93%). Purified using silica gel flash chromatography with heptanes and ethyl acetate. **TLC**: R_f: 0.41 (2:1 heptanes/EtOAc). **IR** (thin film): υ 3011, 2987, 1623, 1569, 1425, 1362, 1216, 1124, 846, 680 cm^{-1}. **^{1}H-NMR** (400 MHz, $CDCl_3$): δ 7.55 (d, *J* = 8.4 Hz, 2H), 7.22 – 7.18 (m, 1H), 7.18 – 7.15 (m, 2H), 7.14 – 7.08 (m, 3H), 5.74 (dd, *J* = 8.6, 4.6 Hz, 1H), 4.29 (dd, *J* = 15.0, 8.6 Hz, 1H), 3.56 (dd,

J = 15.0, 4.6 Hz, 1H), 2.28 (s, 3H). **13C{1H} NMR** (100 MHz, $CDCl_3$): δ 146.0, 135.3, 135.1, 133.9, 130.8, 130.7, 130.2, 128.1, 127.7, 126.0, 83.9, 58.2, 21.7 ppm. **ESI-MS** *m/z* (rel int): (pos) 340.0 ($[M+H]^+$, 100); (neg) 338.0 ($[M–H]^-$, 100). **HRMS** (ESI): $[M+H]^+$ Calculated for: $C_{15}H_{15}ClNO_4S^+$: 340.0405, found: 340.0407. Absolute difference (ppm): 0.59.

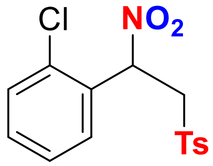

1-chloro-2-(1-nitro-2-tosylethyl)benzene (3s): Alkene **2s** (0.1 mmol) reacted under the general method to produce α-nitrosulfone **3s** as a clear oil (26.8 mg, 0.079 mmol, 79%). Purified using silica gel flash chromatography with heptanes and ethyl acetate. **TLC**: R_f: 0.38 (2:1 heptanes/EtOAc). **IR** (thin film): υ 3001, 2984, 1663, 1575, 1425, 1388, 1216, 1142, 822, 781, 648 cm^{-1}. **^{1}H-NMR** (400 MHz, $CDCl_3$): δ 7.70 (d, *J* = 8.3 Hz, 2H), 7.33 (dd, *J* = 8.0, 1.4 Hz, 1H), 7.27 (d, *J* = 8.6 Hz, 2H), 7.25 – 7.13 (m, 3H), 6.33 (dd, *J* = 8.3, 4.5 Hz, 1H), 4.41 (dd, *J* = 15.1, 8.3 Hz, 1H), 3.64 (dd, *J* = 15.2, 4.5 Hz, 1H), 2.38 (s, 3H). **13C{1H} NMR** (100 MHz, $CDCl_3$): δ 145.8, 135.2, 133.7, 131.4, 130.5, 130.4, 130.2, 130.2, 128.2, 127.9, 81.0, 58.0, 21.7 ppm. **ESI-MS** *m/z* (rel int): (pos) 340.0 ($[M+H]^+$, 100); (neg) 338.0 ($[M–H]^-$, 100). **HRMS** (ESI): $[M+H]^+$ Calculated for: $C_{15}H_{15}ClNO_4S^+$: 340.0405, found: 340.0408. Absolute difference (ppm): 0.88.

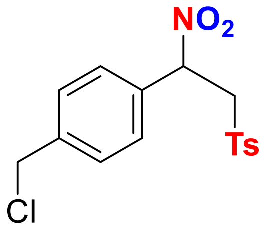

1-(chloromethyl)-4-(1-nitro-2-tosylethyl)benzene (3t): Alkene **2t** (0.1 mmol) reacted under the general method to produce α-nitrosulfone **3t** as a white solid (31.5 mg, 0.093 mmol, 93%). Purified using silica gel flash chromatography with heptanes and ethyl acetate. **TLC**: R_f: 0.30 (2:1 heptanes/EtOAc). **MP**: 135-137 ºC. **IR** (thin film): υ 3016, 2991, 1623, 1571, 1452, 1380, 1205, 1106, 802, 727, 682 cm^{-1}. **^{1}H-NMR** (400 MHz, $CDCl_3$): δ 7.63 (d, *J* = 8.2 Hz, 2H), 7.28 (d, *J* = 7.4 Hz, 3H), 7.27 – 7.23 (m, 3H), 5.86 (dd, *J* = 8.6, 4.5 Hz, 1H), 4.47 (s, 2H), 4.40 (dd, *J* = 15.0, 8.5 Hz, 1H), 3.65 (dd, *J* = 15.0, 4.5 Hz, 1H), 2.38 (s, 3H). **13C{1H} NMR** (100 MHz, $CDCl_3$): δ 145.8, 140.2, 135.2, 132.4, 130.2, 129.5, 128.1, 128.0, 84.2, 58.4, 45.1, 21.8 ppm. **ESI-MS** *m/z* (rel int): (pos) 354.1 ($[M+H]^+$, 100); (neg) 352.1 ($[M–H]^-$, 100). **HRMS** (ESI): $[M+H]^+$ Calculated for: $C_{16}H_{17}ClNO_4S^+$: 354.0561, found: 354.0564. Absolute difference (ppm): 0.85.

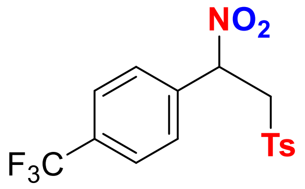

1-methyl-4-((2-nitro-2-(4-(trifluoromethyl)phenyl)ethyl)sulfonyl)benzene (3u): Alkene **2u** (0.1 mmol) reacted under the general method to produce α-nitrosulfone **3u** as a white solid (31.5 mg, 0.084 mmol, 95%). Purified using silica gel flash chromatography with heptanes and ethyl acetate. **TLC**: R_f : 0.43 (2:1 heptanes/EtOAc). **MP**: 141-143 ºC. **IR** (thin film): υ 3023, 2991, 1643, 1575, 1438, 1384, 1232, 1100, 800, 572 cm^{-1}. **^{1}H-NMR** (400 MHz, $CDCl_3$): δ 7.60 (d, *J* = 8.3 Hz, 2H), 7.53 (d, *J* = 8.3 Hz, 2H), 7.40 (d, *J* = 8.2 Hz, 2H), 7.23 (d, *J* = 8.1 Hz, 2H), 5.93 (dd, *J* = 8.0, 5.2 Hz, 1H), 4.38 (dd, *J* = 15.0, 8.0 Hz, 1H), 3.70 (dd, *J* = 14.9, 5.2 Hz, 1H), 2.36 (s, 3H). **13C{1H} NMR** (100 MHz, $CDCl_3$): δ 146.0, 135.8, 135.1, 132.9 (t, *J* = 32.7 Hz), 130.2, 128.3, 128.1, 126.4 (t, *J* = 6.6 Hz), 123.4 (q, *J* = 271.3 Hz), 83.9, 58.2, 21.7 ppm. **ESI-MS** *m/z* (rel int): (pos) 374.1 ($[M+H]^+$, 100); (neg) 372.1 ($[M–H]^-$, 100). **HRMS** (ESI): $[M+H]^+$ Calculated for: $C_{16}H_{15}F_3NO_4S^+$: 374.0668, found: 374.0662. Absolute difference (ppm): 1.60.

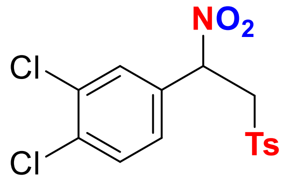

1,2-dichloro-4-(1-nitro-2-tosylethyl)benzene (3v): Alkene **2v** (0.1 mmol) reacted under the general method to produce α-nitrosulfone **3v** as a white solid (33.2 mg, 0.089 mmol, 89%). Purified using silica gel flash chromatography with heptanes and ethyl acetate. **TLC**: R_f : 0.34 (2:1 heptanes/EtOAc). **MP**: 152-155 ºC. **IR** (thin film): υ 3023, 2995, 1609, 1577, 1430, 1384, 1333, 1142, 538 cm^{-1}. **^{1}H-NMR** (400 MHz, $CDCl_3$): δ 7.67 (d, *J* = 8.1 Hz, 2H), 7.43 (d, *J* = 8.4 Hz, 1H), 7.38 (d, *J* = 2.2 Hz, 1H), 7.32 (d, *J* = 8.0 Hz, 2H), 7.21 (dd, *J* = 8.4, 2.2 Hz, 1H), 5.87 (dd, *J* = 7.8, 5.5 Hz, 1H), 4.38 (dd, *J* = 14.9, 7.8 Hz, 1H), 3.74 (dd, *J* = 14.9, 5.5 Hz, 1H), 2.46 (s, 3H). **13C{1H} NMR** (100 MHz, $CDCl_3$): δ 146.1, 135.4, 135.1, 133.8, 131.8, 131.3, 130.2, 129.7, 128.1, 127.1, 83.3, 58.0, 21.7 ppm. **ESI-MS** *m/z* (rel int): (pos) 374.0 ($[M+H]^+$, 100); (neg) 372.0 ($[M–H]^-$, 100). **HRMS** (ESI): $[M+H]^+$ Calculated for: $C_{15}H_{14}Cl_2NO_4S^+$: 374.0015, found: 374.0018. Absolute difference (ppm): 0.80.

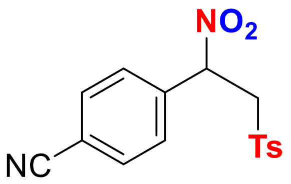

4-(1-nitro-2-tosylethyl)benzonitrile (3w): Alkene **2w** (0.1 mmol) reacted under the general method to produce α-nitrosulfone **3w** as a pale yellow solid (31.5 mg, 0.090 mmol, 90%). Purified using silica gel flash chromatography with heptanes and ethyl acetate. **TLC**: R_f : 0.25 (2:1

heptanes/EtOAc). **MP**: 145-146 ºC. **IR** (thin film): υ 3023, 2991, 2253, 1632, 1571, 1421, 1379, 1201, 824, 648 cm^{-1}. **^{1}H-NMR** (400 MHz, $CDCl_3$): δ 7.74 (d, *J* = 8.2 Hz, 2H), 7.70 (d, *J* = 8.4 Hz, 2H), 7.53 (d, *J* = 8.5 Hz, 2H), 7.37 (d, *J* = 8.0 Hz, 2H), 6.02 (dd, *J* = 8.3, 4.9 Hz, 1H), 4.46 (dd, *J* = 14.9, 8.3 Hz, 1H), 3.74 (dd, *J* = 14.9, 4.9 Hz, 1H), 2.48 (s, 3H). **13C{1H} NMR** (100 MHz, $CDCl_3$): δ 146.2, 136.7, 135.1, 133.1, 130.3, 128.6, 128.1, 117.5, 114.7, 83.8, 58.1, 21.7 ppm. **ESI-MS** *m/z* (rel int): (pos) 331.1 ($[M+H]^+$, 100); (neg) 329.1 ($[M–H]^-$, 100). **HRMS** (ESI): $[M+H]^+$ Calculated for: $C_{16}H_{15}N_2O_4S^+$: 331.0747, found: 331.0744. Absolute difference (ppm): 0.91.

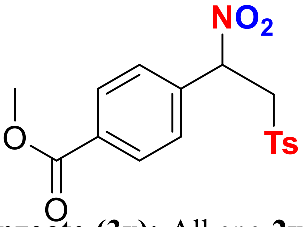

methyl 4-(1-nitro-2-tosylethyl)benzoate (3x): Alkene **2x** (0.1 mmol) reacted under the general method to produce α-nitrosulfone **3x** as a white solid (33.0 mg, 0.091 mmol, 91%). Purified using silica gel flash chromatography with heptanes and ethyl acetate. **TLC**: R_f: 0.26 (2:1 heptanes/EtOAc). **MP**: 134-135 ºC. **IR** (thin film): υ 3041, 2996, 1753, 1609, 1577, 1430, 1386, 1250, 1102, 646 cm^{-1}. **^{1}H-NMR** (400 MHz, $CDCl_3$): δ 7.94 (d, *J* = 8.3 Hz, 2H), 7.65 (d, *J* = 8.1 Hz, 2H), 7.36 (d, *J* = 8.4 Hz, 2H), 7.26 (d, *J* = 8.0 Hz, 2H), 5.92 (dd, *J* = 8.7, 4.3 Hz, 1H), 4.42 (dd, *J* = 15.0, 8.7 Hz, 1H), 3.85 (s, 3H), 3.64 (dd, *J* = 15.0, 4.3 Hz, 1H), 2.37 (s, 3H). **13C{1H} NMR** (100 MHz, $CDCl_3$): δ 165.9, 146.0, 136.6, 135.2, 132.2, 130.6, 130.3, 128.2, 127.7, 84.1, 58.3, 52.5, 21.7 ppm. **ESI-MS** *m/z* (rel int): (pos) 364.1 ($[M+H]^+$, 100); (neg) 362.1 ($[M–H]^-$, 100). **HRMS** (ESI): $[M+H]^+$ Calculated for: $C_{17}H_{18}NO_6S^+$: 364.0849, found: 364.0845. Absolute difference (ppm): 1.10.

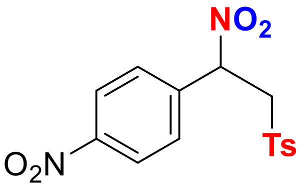

1-methyl-4-((2-nitro-2-(4-nitrophenyl)ethyl)sulfonyl)benzene (3y): Alkene **2y** (0.1 mmol) reacted under the general method to produce α-nitrosulfone **3y** as a white solid (31.2 mg, 0.089 mmol, 89%). Purified using silica gel flash chromatography with heptanes and ethyl acetate. **TLC**: R_f: 0.26 (2:1 heptanes/EtOAc). **MP**: 143-145 ºC. **IR** (thin film): υ 3023, 2993, 1601, 1577, 1553, 1403, 1388, 1344, 1142, 701, 538 cm^{-1}. **^{1}H-NMR** (400 MHz, $CDCl_3$): δ 8.15 (d, *J* = 8.7 Hz, 2H), 7.65 (d, *J* = 8.0 Hz, 2H), 7.51 (d, *J* = 8.4 Hz, 2H), 7.28 (d, *J* = 7.7 Hz, 2H), 5.99 (dd, *J* = 8.1, 5.1 Hz, 1H), 4.39 (dd, *J* = 14.9, 8.1 Hz, 1H), 3.69 (dd, *J* = 14.9, 5.1 Hz, 1H), 2.38 (s, 3H). **13C{1H} NMR** (100 MHz, $CDCl_3$): δ 149.1, 146.2, 138.4, 135.1, 130.3, 129.0, 128.1, 124.5, 83.5, 58.2, 21.7 ppm. **ESI-MS** *m/z* (rel int): (pos) 351.1 ($[M+H]^+$, 100); (neg) 349.1 ($[M–H]^-$, 100). **HRMS** (ESI): $[M+H]^+$ Calculated for: $C_{15}H_{15}N_2O_6S^+$: 351.0645, found: 351.0641. Absolute difference (ppm): 1.14.

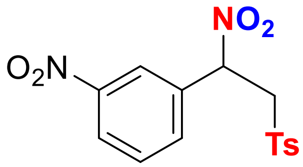

1-nitro-3-(1-nitro-2-tosylethyl)benzene (3z): Alkene **2z** (0.1 mmol) reacted under the general method to produce α-nitrosulfone **3z** as a yellow oil (30.8 mg, 0.088 mmol, 88%). Purified using silica gel flash chromatography with heptanes and ethyl acetate. **TLC**: R_f: 0.24 (2:1 heptanes/EtOAc). **IR** (thin film): υ 3014, 2991, 1605, 1575, 1535, 1401, 1382, 1304, 1124, 736, 583 cm^{-1}. **^{1}H-NMR** (400 MHz, $CDCl_3$): δ 8.21 (dd, *J* = 8.2, 2.2 Hz, 1H), 8.11 (t, *J* = 2.0 Hz, 1H), 7.70 (d, *J* = 7.9 Hz, 1H), 7.64 (d, *J* = 8.4 Hz, 2H), 7.54 (t, *J* = 8.0 Hz, 1H), 7.26 (d, *J* = 8.4 Hz, 2H), 5.98 (dd, *J* = 7.9, 5.5 Hz, 1H), 4.40 (dd, *J* = 14.9, 7.9 Hz, 1H), 3.76 (dd, *J* = 14.9, 5.5 Hz, 1H), 2.37 (s, 3H). **13C{1H} NMR** (100 MHz, $CDCl_3$): δ 148.5, 146.2, 135.1, 134.1, 133.8, 130.7, 130.3, 128.1, 125.5, 122.8, 83.5, 58.0, 21.7 ppm. **ESI-MS** *m/z* (rel int): (pos) 351.1 ([M+H]$^+$, 100); (neg) 349.1 ([M−H]$^-$, 100). **HRMS** (ESI): [M+H]$^+$ Calculated for: $C_{15}H_{15}N_2O_6S^+$: 351.0645, found: 351.0648. Absolute difference (ppm): 0.85.

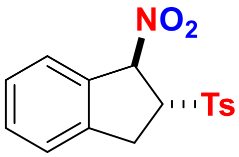

(1R,2R)-1-nitro-2-tosyl-2,3-dihydro-1H-indene (3aa): Alkene **2aa** (0.1 mmol) reacted under the general method to produce α-nitrosulfone **3aa** as a clear oil (23 mg, 0.073 mmol, 73%). Purified using silica gel flash chromatography with heptanes and ethyl acetate. **TLC**: R_f: 0.42 (2:1 heptanes/EtOAc). **IR** (thin film): υ 3041, 2998, 1645, 1535, 1463, 1373, 1322, 1142, 844 cm^{-1}. **^{1}H-NMR** (400 MHz, $CDCl_3$): δ 7.87 (d, *J* = 8.3 Hz, 2H), 7.52 (d, *J* = 7.5 Hz, 1H), 7.44 – 7.36 (m, 3H), 7.35 – 7.29 (m, 1H), 5.78 (d, *J* = 8.4 Hz, 1H), 4.46 (dd, *J* = 8.4, 2.4 Hz, 1H), 3.28 (dd, *J* = 16.0, 9.2 Hz, 1H), 3.13 (dd, *J* = 16.0, 8.8 Hz, 1H), 2.47 (s, 3H). **13C{1H} NMR** (100 MHz, $CDCl_3$): δ 142.1, 138.4, 136.6, 136.2, 129.9, 129.7, 128.5, 128.2, 128.1, 127.6, 91.6, 66.4, 33.2, 21.5 ppm. **ESI-MS** *m/z* (rel int): (pos) 318.1 ([M+H]$^+$, 100); (neg) 316.1 ([M−H]$^-$, 100). **HRMS** (ESI): [M+H]$^+$ Calculated for: $C_{16}H_{16}NO_4S^+$: 318.0795, found: 318.0791. Absolute difference (ppm): 1.26.

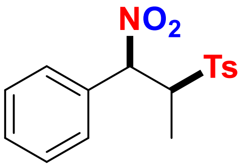

1-methyl-4-(((1R,2S)-1-nitro-1-phenylpropan-2-yl)sulfonyl)benzene (3ab): Alkene **2ab** (0.1 mmol) reacted under the general method to produce α-nitrosulfone **3ab** as a clear oil (24 mg, 0.075 mmol, 75%). Purified using silica gel flash chromatography with heptanes and ethyl acetate. **TLC**: R_f: 0.40 (2:1 heptanes/EtOAc). **IR** (thin film): υ 3032, 2991, 1623, 1571, 1436, 1375, 1322, 1140, 804 cm^{-1}. **^{1}H-NMR** (400 MHz, $CDCl_3$): δ 7.83 (d, *J* = 8.3 Hz, 2H), 7.50 – 7.34 (m, 7H), 5.77 (d, *J* = 10.1 Hz, 1H), 4.44 (dq, *J* = 10.1, 7.3 Hz, 1H), 2.49 (s, 3H), 1.02 (d, *J* = 7.3 Hz, 3H). **13C{1H}**

NMR (100 MHz, $CDCl_3$): δ 145.6, 135.1, 132.2, 130.4, 130.4, 129.2, 128.1, 127.6, 82.6, 57.4, 21.5, 10.7 ppm. **ESI-MS** *m/z* (rel int): (pos) 320.1 ($[M+H]^+$, 100); (neg) 318.1 ($[M-H]^-$, 100). **HRMS** (ESI): $[M+H]^+$ Calculated for: $C_{16}H_{18}NO_4S^+$: 320.0951, found: 320.0955. Absolute difference (ppm): 1.25.

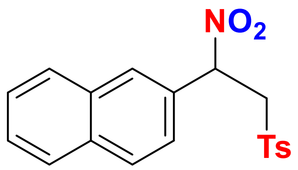

2-(1-nitro-2-tosylethyl)naphthalene (3ac): Alkene **2ac** (0.1 mmol) reacted under the general method to produce α-nitrosulfone **3ac** as a pale yellow solid (30.5 mg, 0.086 mmol, 86%). Purified using silica gel flash chromatography with heptanes and ethyl acetate. **TLC**: R_f: 0.43 (2:1 heptanes/EtOAc). **MP**: 152-154 °C. **IR** (thin film): υ 3032, 3023, 2995, 1623, 1577, 1388, 1304, 1160, 934, 844, 538 cm^{-1}. **^{1}H-NMR** (400 MHz, $CDCl_3$): δ 7.76 – 7.71 (m, 4H), 7.63 (d, *J* = 8.4 Hz, 2H), 7.49 – 7.45 (m, 2H), 7.31 (d, *J* = 8.7 Hz, 1H), 7.17 (d, *J* = 8.1 Hz, 2H), 6.02 (dd, *J* = 8.5, 4.5 Hz, 1H), 4.51 (dd, *J* = 15.0, 8.5 Hz, 1H), 3.76 (dd, *J* = 15.0, 4.5 Hz, 1H), 2.30 (s, 3H). **13C{1H} NMR** (100 MHz, $CDCl_3$): δ 145.7, 135.3, 133.9, 132.8, 130.0, 129.6, 129.5, 128.3, 128.1, 127.8, 127.7, 127.2, 123.4, 84.8, 58.5, 21.7 ppm. **ESI-MS** *m/z* (rel int): (pos) 356.1 ($[M+H]^+$, 100); (neg) 354.1 ($[M-H]^-$, 100). **HRMS** (ESI): $[M+H]^+$ Calculated for: $C_{19}H_{18}NO_4S^+$: 356.0951, found: 356.0953. Absolute difference (ppm): 0.56.

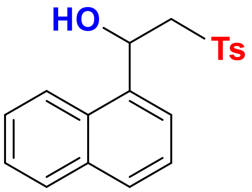

1-(naphthalen-1-yl)-2-tosylethan-1-ol (4ad): Alkene **2ad** (0.1 mmol) reacted under the general method to produce hydroxysulfone **4ad** as a yellow solid (20.9 mg, 0.064 mmol, 64%). Purified using silica gel flash chromatography with heptanes and ethyl acetate. **TLC**: R_f: 0.32 (2:1 heptanes/EtOAc). **MP**: 131-132 °C. **IR** (thin film): υ 3495, 2999, 1636, 1412, 1250, 1106, 964, 842 cm^{-1}. **^{1}H-NMR** (400 MHz, $CDCl_3$): δ 7.91 (d, *J* = 8.3 Hz, 2H), 7.84 (d, *J* = 8.0 Hz, 1H), 7.79 – 7.73 (m, 2H), 7.49 (d, *J* = 8.3 Hz, 1H), 7.48 – 7.43 (m, 2H), 7.41 – 7.37 (m, 3H), 6.06 (dd, *J* = 7.7, 3.5 Hz, 1H), 3.84 (d, *J* = 4.0 Hz, 1H), 3.51 – 3.49 (m, 2H), 2.49 (s, 3H). **13C{1H} NMR** (100 MHz, $CDCl_3$): δ 145.4, 136.1, 135.9, 133.7, 130.2, 129.2, 128.7, 128.2, 126.6, 125.7, 125.6, 123.2, 121.8, 65.2, 63.5, 21.8 ppm. **ESI-MS** *m/z* (rel int): (pos) 327.1 ($[M+H]^+$, 100); (neg) 325.1 ($[M-H]^-$, 100). **HRMS** (ESI): $[M+H]^+$ Calculated for: $C_{19}H_{19}O_3S^+$: 327.1049, found: 327.1045. Absolute difference (ppm): 1.22.

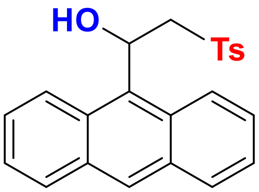

1-(anthracen-9-yl)-2-tosylethan-1-ol (4ae): Alkene **2ae** (0.1 mmol) reacted under the general method to produce hydroxysulfone **4ae** as a yellow solid (23.3 mg, 0.062 mmol, 62%). Purified using silica gel flash chromatography with heptanes and ethyl acetate. **TLC**: *Rf*: 0.34 (2:1 heptanes/EtOAc). MP: 157-158 ºC. **IR** (thin film): υ 3499, 2993, 1645, 1418, 1247, 1151, 982, 804 cm^{-1}. **^{1}H-NMR** (400 MHz, $CDCl_3$): δ 8.41 (s, 1H), 7.98 – 7.92 (m, 3H), 7.85 (d, *J* = 8.0 Hz, 2H), 7.45 (dt, *J* = 6.4, 4.0 Hz, 4H), 7.37 (d, *J* = 8.0 Hz, 2H), 6.85 (dd, *J* = 10.1, 2.0 Hz, 1H), 4.31 (dd, *J* = 14.7, 10.0 Hz, 1H), 3.68 (d, *J* = 4.0 Hz, 1H), 3.47 (dd, *J* = 14.7, 1.9 Hz, 1H), 2.48 (s, 3H). **13C{1H} NMR** (100 MHz, $CDCl_3$): δ 145.2, 136.2, 131.7, 130.2, 130.1, 130.0, 129.5, 129.4, 129.2, 128.1, 126.6, 126.3, 124.9, 65.4, 62.3, 21.7 ppm. **ESI-MS** *m/z* (rel int): (pos) 377.1 ($[M+H]^+$, 100); (neg) 375.1 ($[M–H]^-$, 100). **HRMS** (ESI): $[M+H]^+$ Calculated for: $C_{23}H_{21}O_3S^+$: 377.1206, found: 377.1203. Absolute difference (ppm): 0.80.

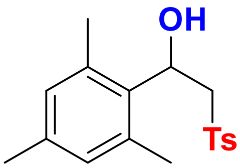

1-mesityl-2-tosylethan-1-ol (4af): Alkene **2af** (0.1 mmol) reacted under the general method to produce hydroxysulfone **4af** as a clear oil (22.6 mg, 0.071 mmol, 71%). Purified using silica gel flash chromatography with heptanes and ethyl acetate. **TLC**: *Rf*: 0.38 (2:1 heptanes/EtOAc). **IR** (thin film): υ 3499, 2998, 1643, 1414, 1232, 1133, 907, 817 cm^{-1}. **^{1}H-NMR** (400 MHz, $CDCl_3$): δ 7.86 (d, *J* = 8.1 Hz, 2H), 7.40 (d, *J* = 8.0 Hz, 2H), 6.76 (s, 2H), 5.62 (dd, *J* = 10.1, 1.6 Hz, 1H), 3.85 (dd, *J* = 14.6, 10.1 Hz, 1H), 3.22 (dd, *J* = 14.7, 1.6 Hz, 1H), 2.47 (s, 3H), 2.24 (s, 3H), 2.21 (s, 6H). **13C{1H} NMR** (100 MHz, $CDCl_3$): δ 145.2, 137.7, 136.1, 136.0, 133.0, 130.3, 130.1, 128.1, 65.5, 61.2, 21.7, 20.7, 20.4 ppm. **ESI-MS** *m/z* (rel int): (pos) 319.1 ($[M+H]^+$, 100); (neg) 317.1 ($[M–H]^-$, 100). **HRMS** (ESI): $[M+H]^+$ Calculated for: $C_{18}H_{23}O_3S^+$: 319.1362, found: 319.1365. Absolute difference (ppm): 0.94.

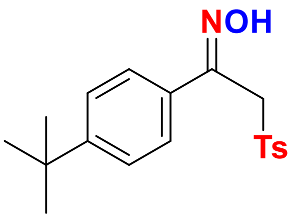

1-(4-(tert-butyl)phenyl)-2-tosylethan-1-one oxime (5a): Alkene **2a** (0.1 mmol) reacted under the general method to produce oxime **5a** as a white solid (18.3 mg, 0.053 mmol, 53%). Purified using silica gel flash chromatography with heptanes and ethyl acetate. **TLC**: *Rf*: 0.26 (2:1 heptanes/EtOAc).[1] MP: 174-175 ºC. **^{1}H-NMR** (400 MHz, $CDCl_3$): δ 7.61 (d, *J* = 8.3 Hz, 2H), 7.46

(d, *J* = 8.6 Hz, 2H), 7.26 (d, *J* = 8.6 Hz, 2H), 7.13 (d, *J* = 8.1 Hz, 2H), 4.63 (s, 1H), 2.31 (s, 2H), 1.23 (s, 9H). **13C{1H} NMR** (100 MHz, $CDCl_3$): δ 153.2, 148.0, 144.7, 136.6, 130.7, 129.4, 128.6, 126.4, 125.6, 52.5, 34.7, 31.2, 21.7 ppm. **ESI-MS** *m/z* (rel int): (pos) 346.1 ($[M+H]^+$, 100); (neg) 344.1 ($[M-H]^-$, 100). **HRMS** (ESI): $[M+H]^+$ Calculated for: $C_{19}H_{24}NO_3S^+$: 346.1471, found: 346.1476. Absolute difference (ppm): 1.44.

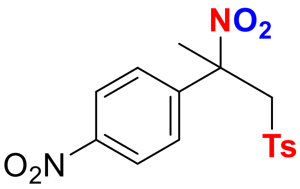

1-methyl-4-((2-nitro-2-(4-nitrophenyl)propyl)sulfonyl)benzene (3ag): Alkene **2ag** (0.1 mmol) reacted under the general method to produce nitrososulfone **3ag** as a pale yellow oil (5.82 mg, 0.016 mmol, 16%). Purified using silica gel flash chromatography with heptanes and ethyl acetate. **TLC**: R_f : 0.32 (2:1 heptanes/EtOAc). **IR** (thin film): υ 3032, 2999, 1625, 1575, 1546, 1430, 1382, 1348, 1106, 754, 589 cm^{-1}. **^{1}H-NMR** (400 MHz, $CDCl_3$): δ 8.16 (d, *J* = 9.0 Hz, 2H), 7.62 (d, *J* = 8.4 Hz, 2H), 7.49 (d, *J* = 9.0 Hz, 2H), 7.29 (d, *J* = 8.1 Hz, 2H), 4.31 (d, *J* = 14.7 Hz, 1H), 4.13 (d, *J* = 14.7 Hz, 1H), 2.46 (s, 3H), 2.44 (s, 3H). **13C{1H} NMR** (100 MHz, $CDCl_3$): δ 148.4, 145.7, 142.8, 136.9, 130.1, 127.8, 127.0, 124.1, 89.3, 63.4, 23.4, 21.7 ppm. **ESI-MS** *m/z* (rel int): (pos) 365.1 ($[M+H]^+$, 100); (neg) 363.1 ($[M-H]^-$, 100). **HRMS** (ESI): $[M+H]^+$ Calculated for: $C_{16}H_{17}N_2O_6S^+$: 365.0802, found: 365.0806. Absolute difference (ppm): 1.10.

References:

1. Wang, B.; Yan, Z.; Liu, L.; Wang, J.; Zha, Z.; Wang, Z. TBN-Mediated Regio- and Stereoselective Sulfonylation and Oximation (Oximosulfonation) of Alkynes with Sulfonyl Hydrazines in EtoH/H2O. *Green Chem.* **2019**, *21*, 205-212.

D. ^{1}H-NMR AND ^{13}C-NMR SPECTRA

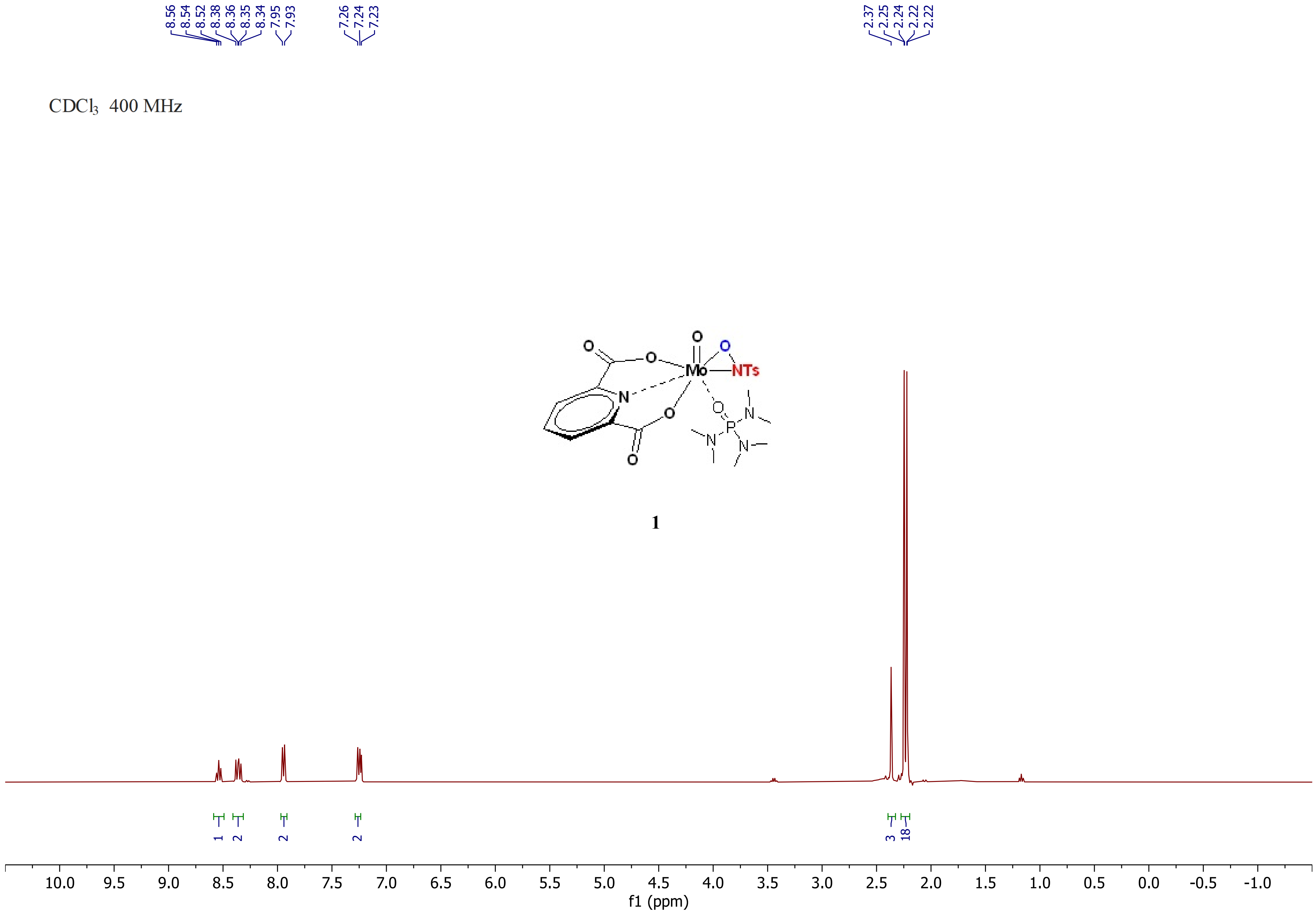

CDCl$_3$ 400 MHz
8.56
8.54
8.52
8.38
8.36
8.35
8.34
7.95
7.93
7.26
7.24
7.23
2.37
2.25
2.24
2.22
2.22
O
Mo
NTs
N
O
P
1
1
2
2
2
3
18
10.0 9.5 9.0 8.5 8.0 7.5 7.0 6.5 6.0 5.5 5.0 4.5 4.0 3.5 3.0 2.5 2.0 1.5 1.0 0.5 0.0 -0.5 -1.0
f1 (ppm)

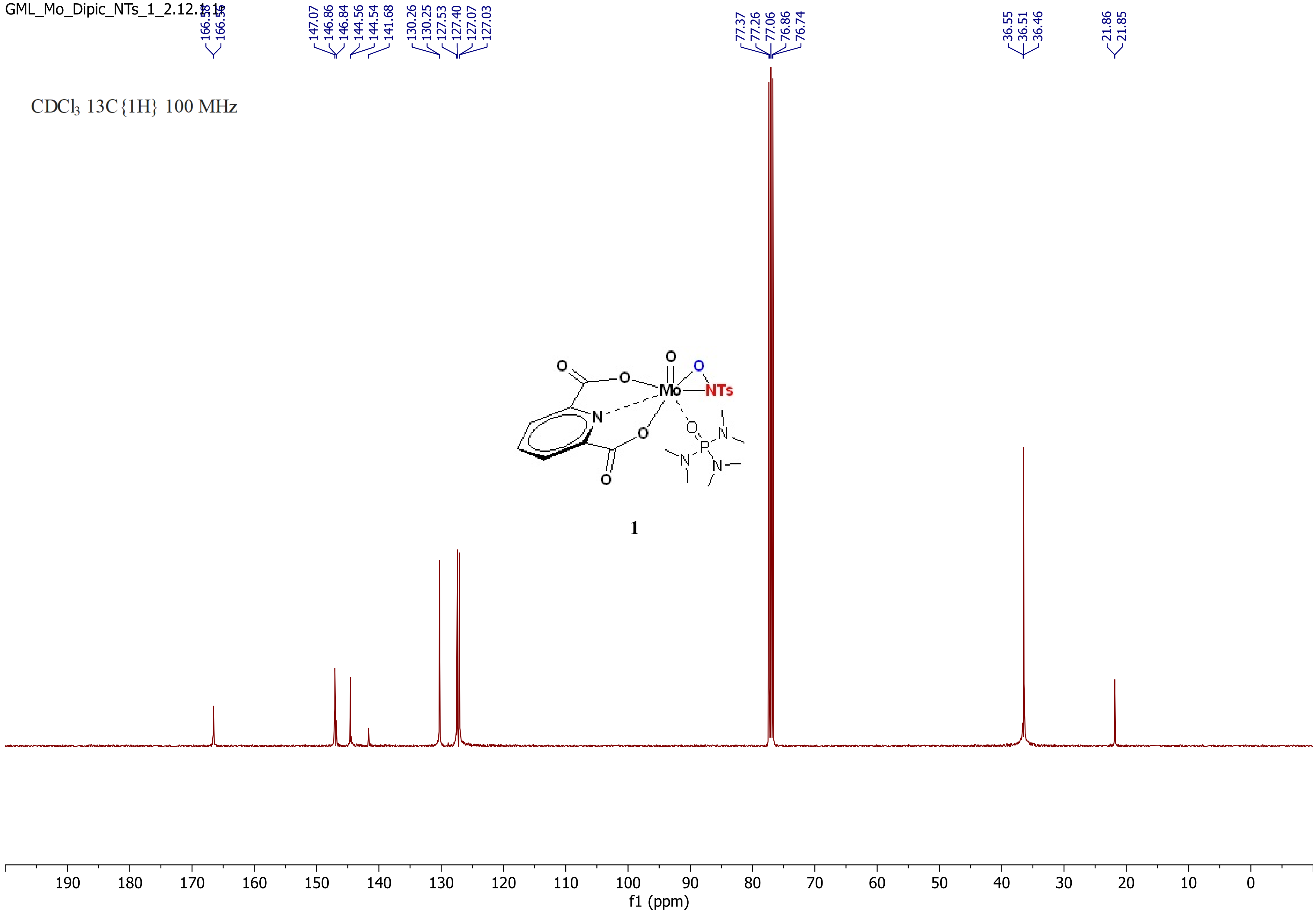
GML_Mo_Dipic_NTs_1_2.12.1.1r
166.58
166.56
147.07
146.86
146.84
144.56
144.54
141.68
130.26
130.25
127.53
127.40
127.07
127.03
77.37
77.26
77.06
76.86
76.74
36.55
36.51
36.46
21.86
21.85
CDCl3 13C{1H} 100 MHz
O
Mo
NTs
N
P
1
190 180 170 160 150 140 130 120 110 100 90 80 70 60 50 40 30 20 10 0
f1 (ppm)

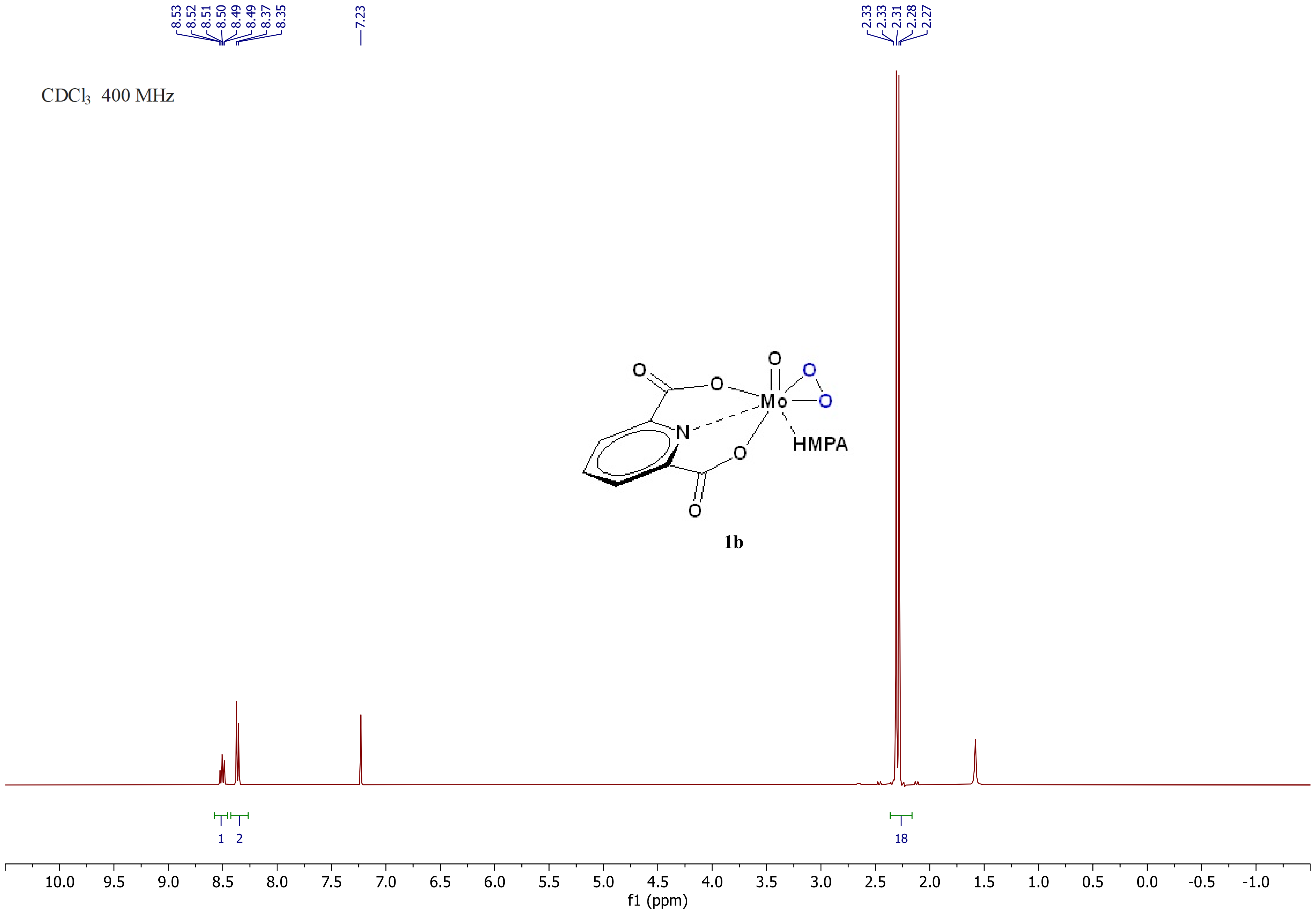

8.53
8.52
8.51
8.50
8.49
8.49
8.37
8.35
7.23
2.33
2.33
2.31
2.28
2.27
CDCl3 400 MHz
O
O
O
Mo
O
O
N
O
HMPA
O
1b
1
2
18
10.0
9.5
9.0
8.5
8.0
7.5
7.0
6.5
6.0
5.5
5.0
4.5
4.0
3.5
3.0
2.5
2.0
1.5
1.0
0.5
0.0
-0.5
-1.0
f1 (ppm)

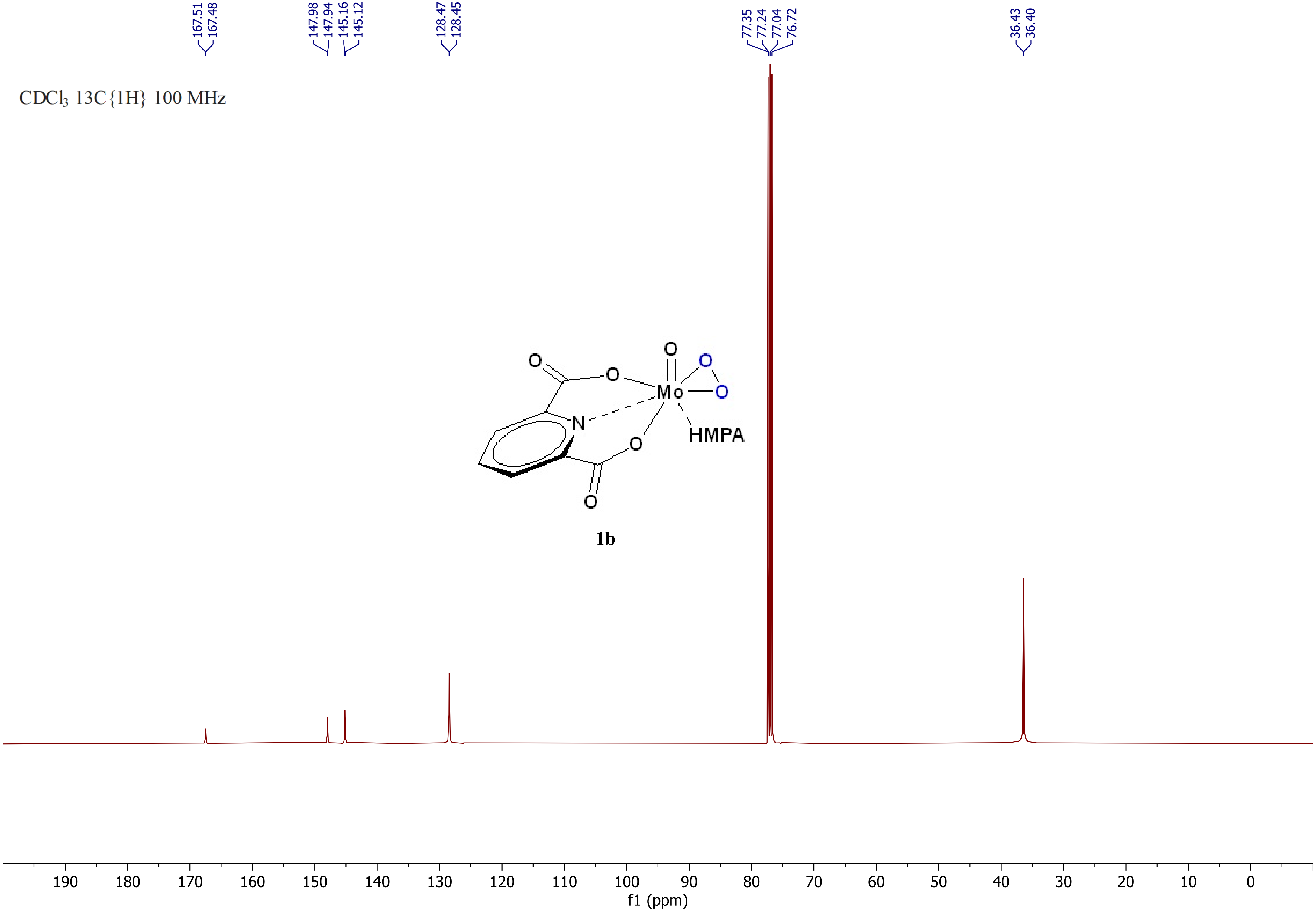

167.51
167.48
147.98
147.94
145.16
145.12
128.47
128.45
77.35
77.24
77.04
76.72
36.43
36.40
CDCl3 13C{1H} 100 MHz
O
O
Mo
O
O
O
N
HMPA
O
O
1b
190 180 170 160 150 140 130 120 110 100 90 80 70 60 50 40 30 20 10 0
f1 (ppm)

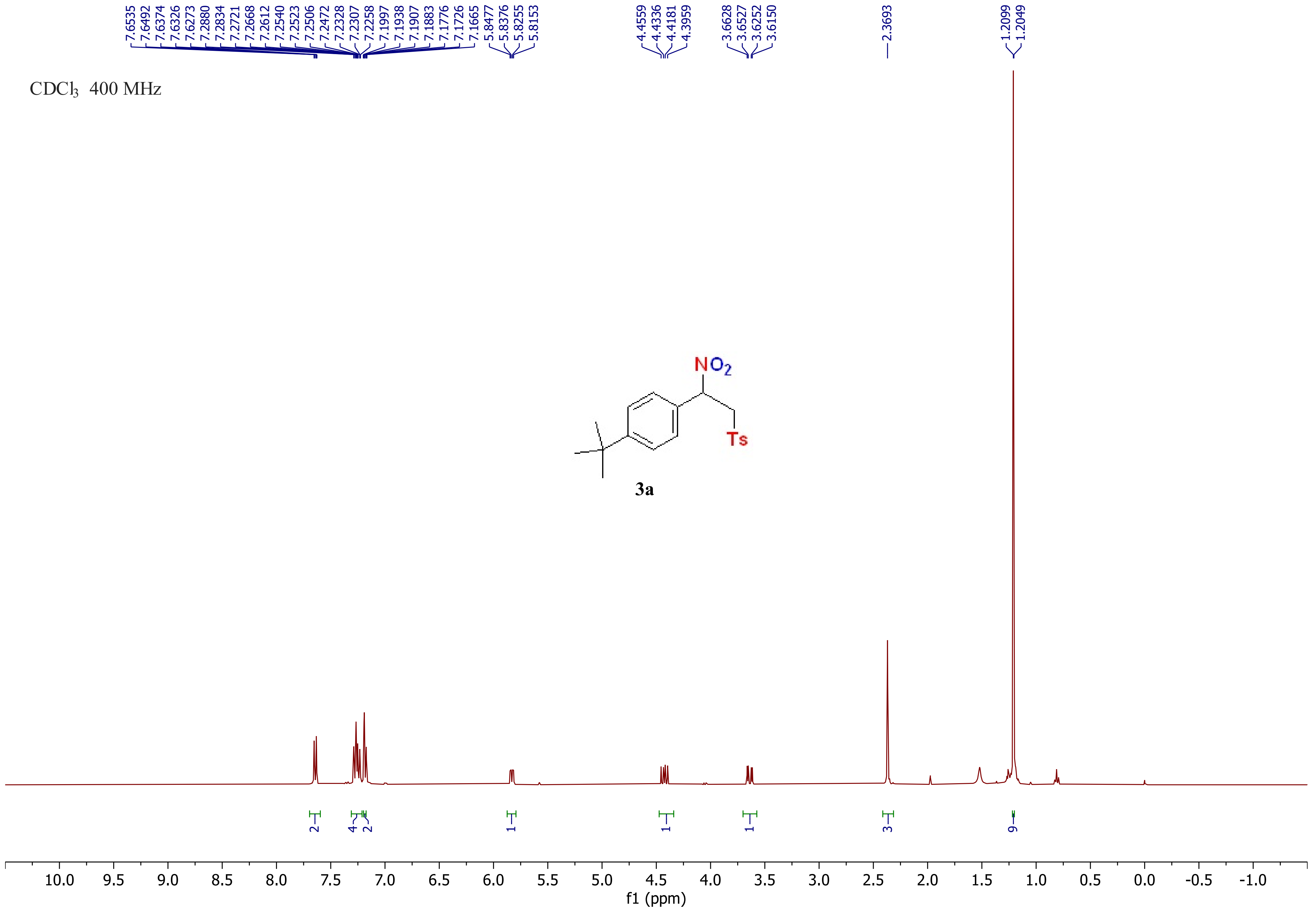
7.6535
7.6492
7.6374
7.6326
7.6273
7.2880
7.2834
7.2721
7.2668
7.2612
7.2540
7.2523
7.2506
7.2472
7.2328
7.2307
7.2258
7.1997
7.1938
7.1907
7.1883
7.1776
7.1726
7.1665
5.8477
5.8376
5.8255
5.8153
4.4559
4.4336
4.4181
4.3959
3.6628
3.6527
3.6252
3.6150
2.3693
1.2099
1.2049
CDCl3 400 MHz
NO2
Ts
3a
2
4
2
1
1
1
3
9
10.0 9.5 9.0 8.5 8.0 7.5 7.0 6.5 6.0 5.5 5.0 4.5 4.0 3.5 3.0 2.5 2.0 1.5 1.0 0.5 0.0 -0.5 -1.0
f1 (ppm)

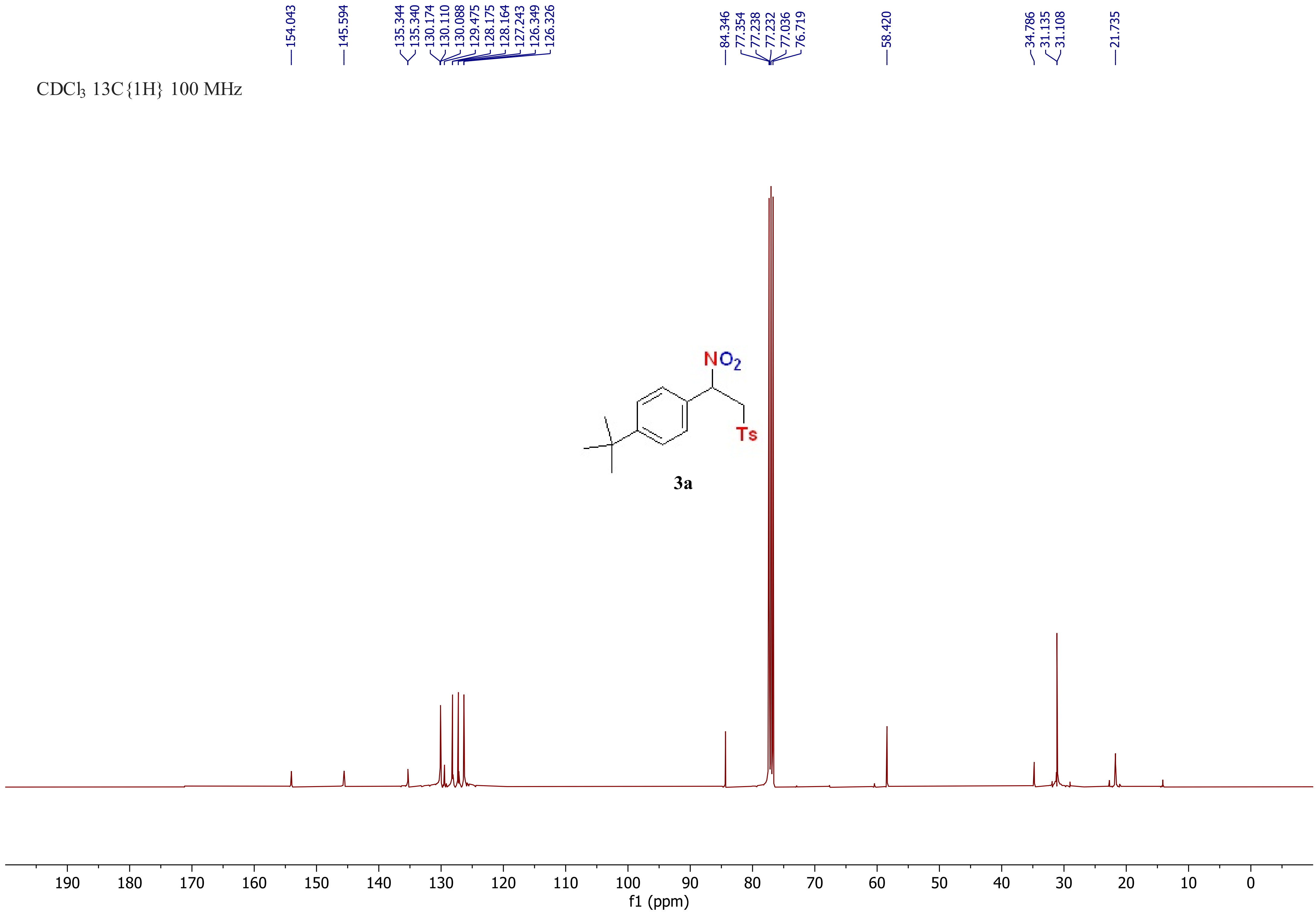
154.043
145.594
135.344
135.340
130.174
130.110
130.088
129.475
128.175
128.164
127.243
126.349
126.326
84.346
77.354
77.238
77.232
77.036
76.719
58.420
34.786
31.135
31.108
21.735
CDCl3 13C{1H} 100 MHz
NO2
Ts
3a
190
180
170
160
150
140
130
120
110
100
90
80
70
60
50
40
30
20
10
0
f1 (ppm)

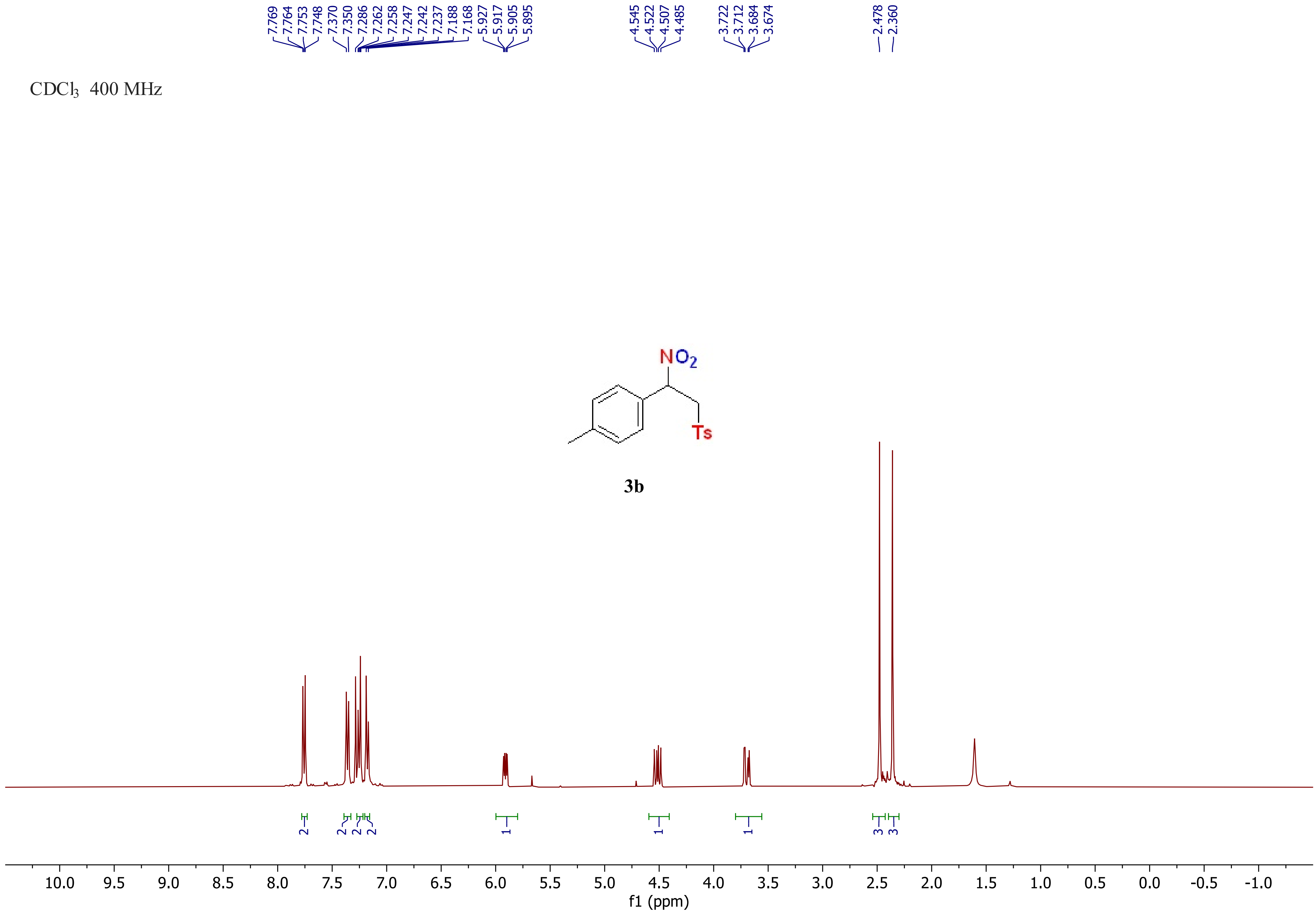
7.769
7.764
7.753
7.748
7.370
7.350
7.286
7.262
7.258
7.247
7.242
7.237
7.188
7.168
5.927
5.917
5.905
5.895
4.545
4.522
4.507
4.485
3.722
3.712
3.684
3.674
2.478
2.360
CDCl3 400 MHz
NO2
Ts
3b
2
2
2
2
1
1
1
3
3
10.0
9.5
9.0
8.5
8.0
7.5
7.0
6.5
6.0
5.5
5.0
4.5
4.0
3.5
3.0
2.5
2.0
1.5
1.0
0.5
0.0
-0.5
-1.0
f1 (ppm)

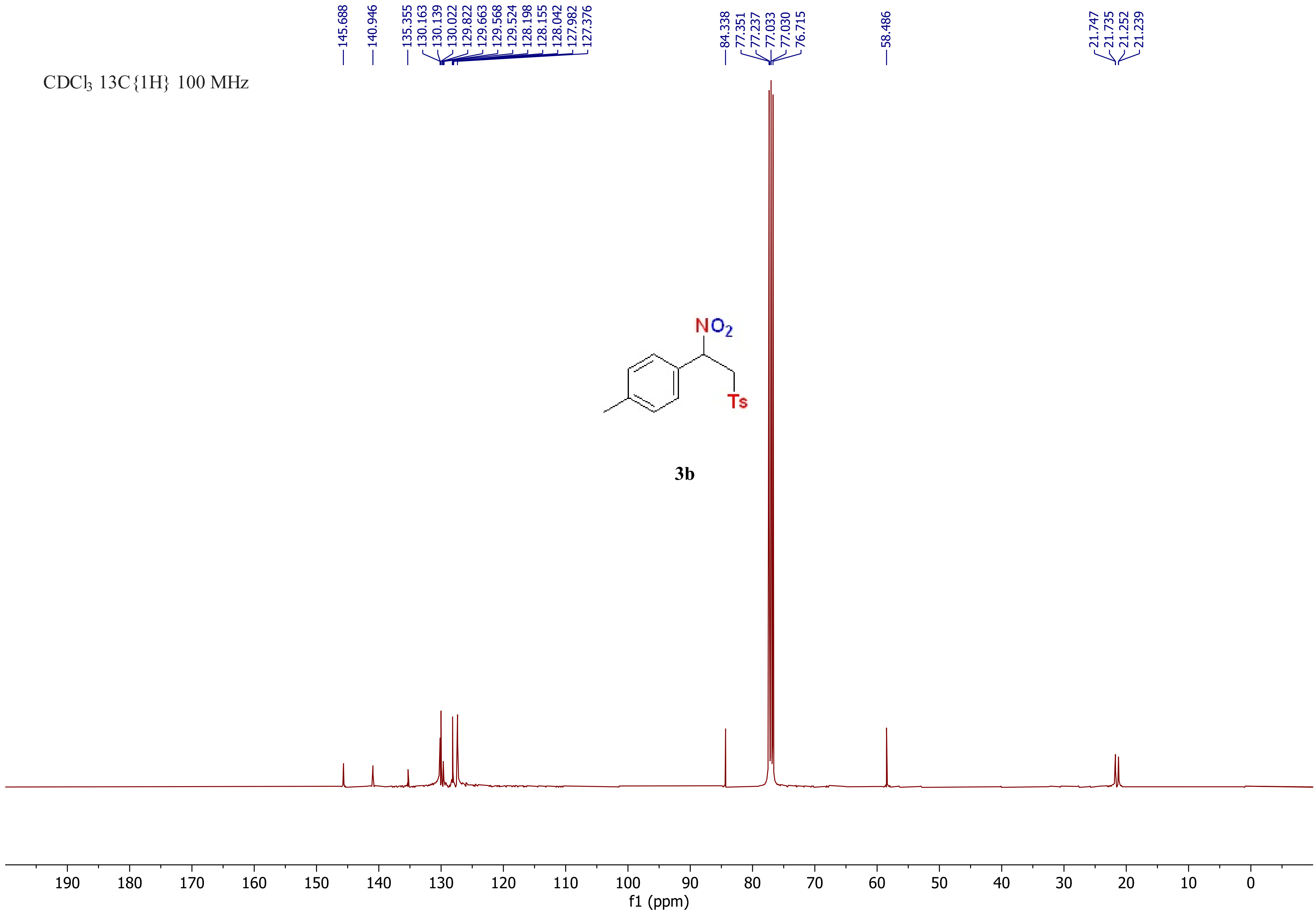
CDCl₃ 13C{1H} 100 MHz
145.688
140.946
135.355
130.163
130.139
130.022
129.822
129.663
129.568
129.524
128.198
128.155
128.042
127.982
127.376
84.338
77.351
77.237
77.033
77.030
76.715
58.486
21.747
21.735
21.252
21.239
NO2
Ts
3b
190 180 170 160 150 140 130 120 110 100 90 80 70 60 50 40 30 20 10 0
f1 (ppm)

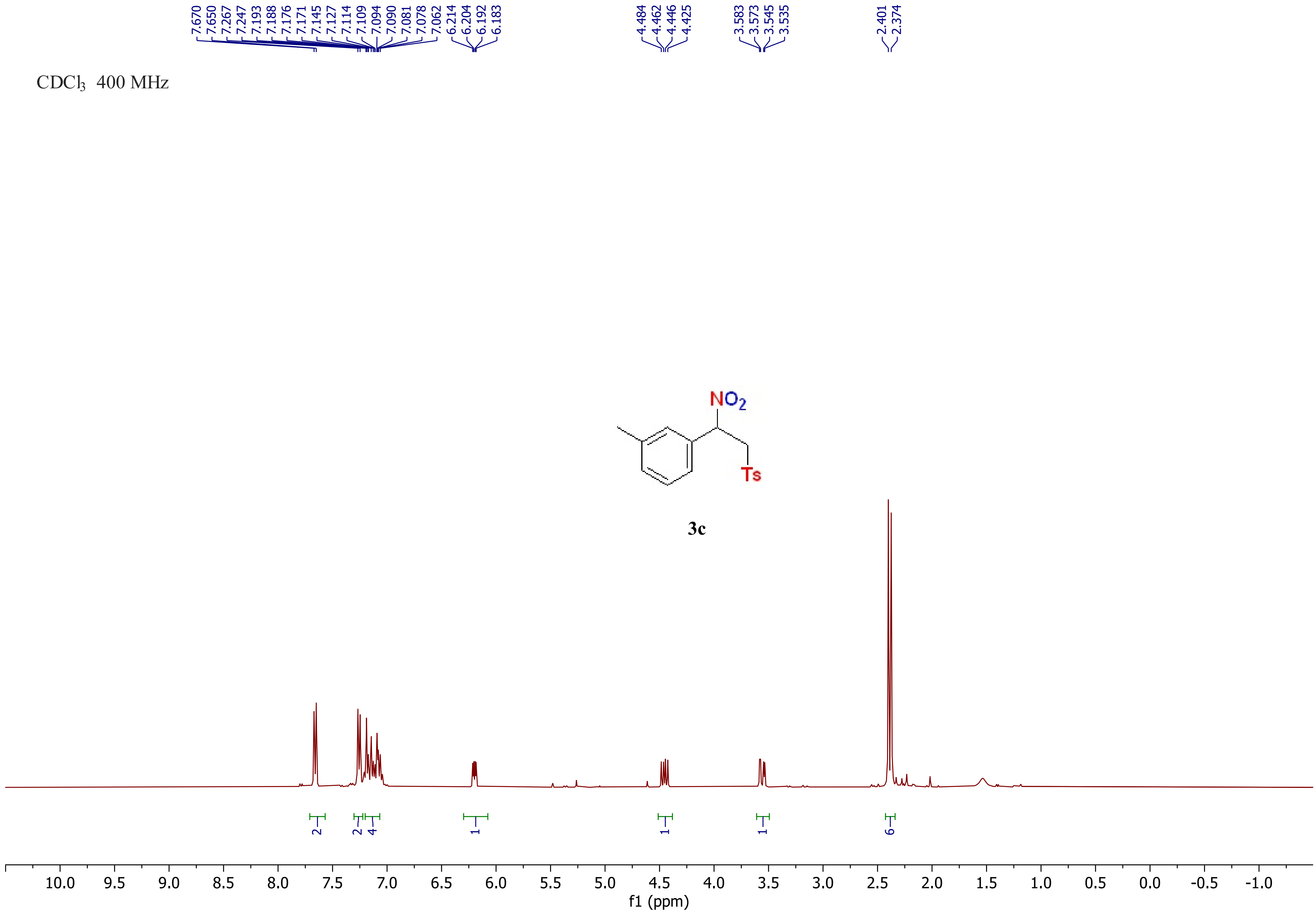

7.670
7.650
7.267
7.247
7.193
7.188
7.176
7.171
7.145
7.127
7.114
7.109
7.094
7.090
7.081
7.078
7.062
6.214
6.204
6.192
6.183
4.484
4.462
4.446
4.425
3.583
3.573
3.545
3.535
2.401
2.374
CDCl3 400 MHz
NO2
Ts
3c
2
2
4
1
1
1
6
10.0 9.5 9.0 8.5 8.0 7.5 7.0 6.5 6.0 5.5 5.0 4.5 4.0 3.5 3.0 2.5 2.0 1.5 1.0 0.5 0.0 -0.5 -1.0
f1 (ppm)

CDCl$_3$ 13C{1H} 100 MHz

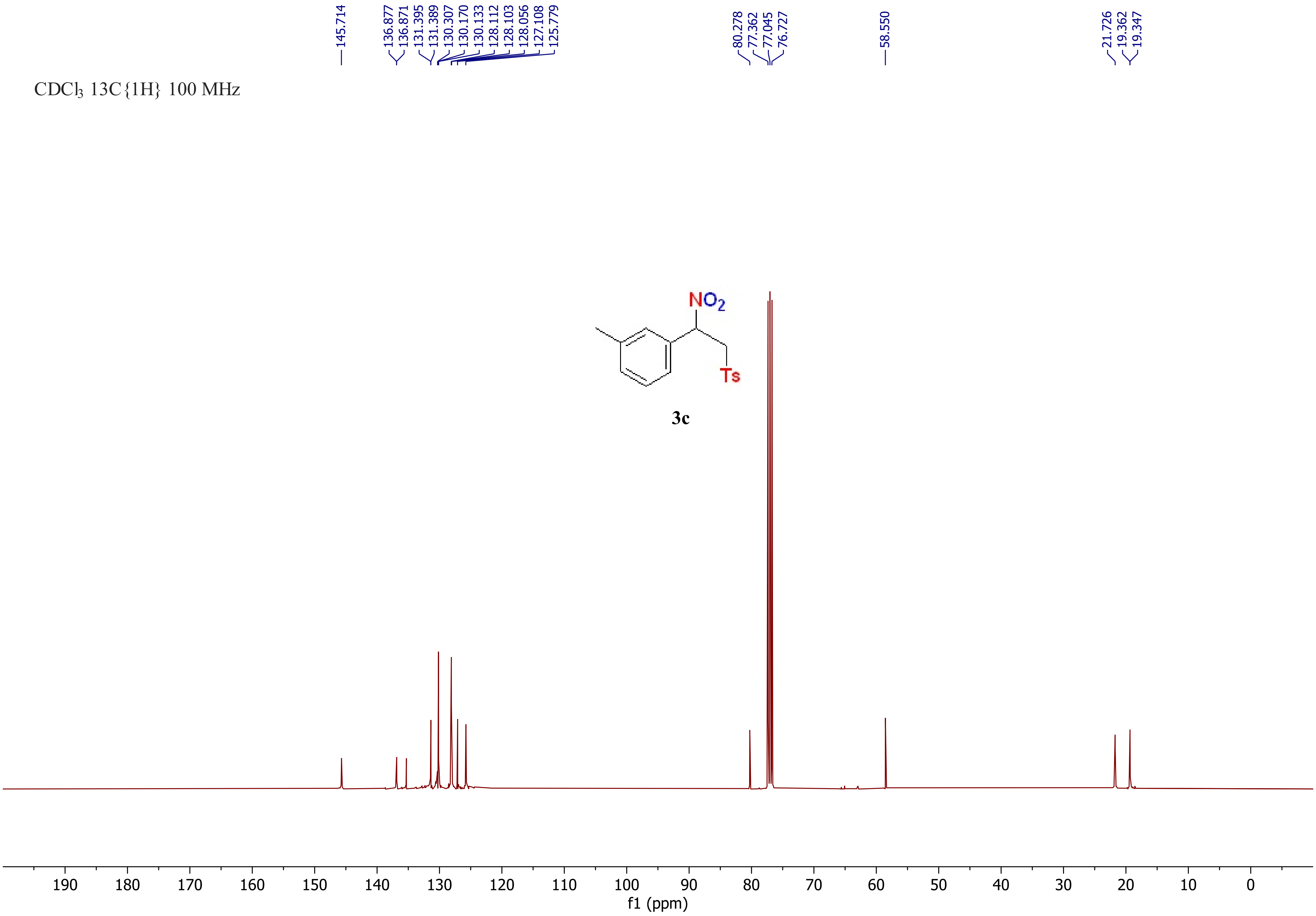

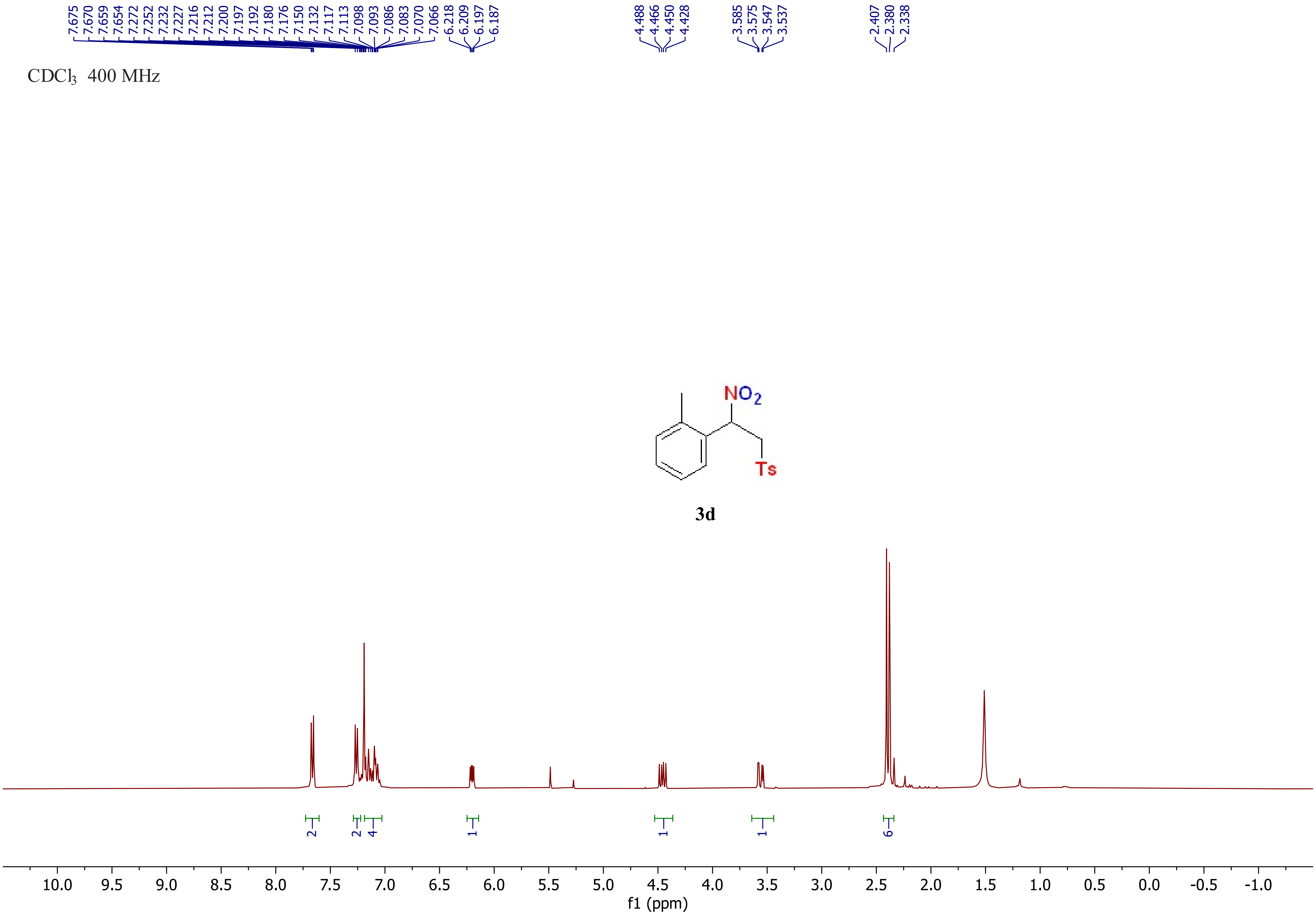

7.675
7.670
7.659
7.654
7.272
7.252
7.232
7.227
7.216
7.212
7.200
7.197
7.192
7.180
7.176
7.150
7.132
7.117
7.113
7.098
7.093
7.086
7.083
7.070
7.066
6.218
6.209
6.197
6.187
4.488
4.466
4.450
4.428
3.585
3.575
3.547
3.537
2.407
2.380
2.338
CDCl3 400 MHz
NO2
Ts
3d
2
2
4
1
1
1
6
10.0 9.5 9.0 8.5 8.0 7.5 7.0 6.5 6.0 5.5 5.0 4.5 4.0 3.5 3.0 2.5 2.0 1.5 1.0 0.5 0.0 -0.5 -1.0
f1 (ppm)

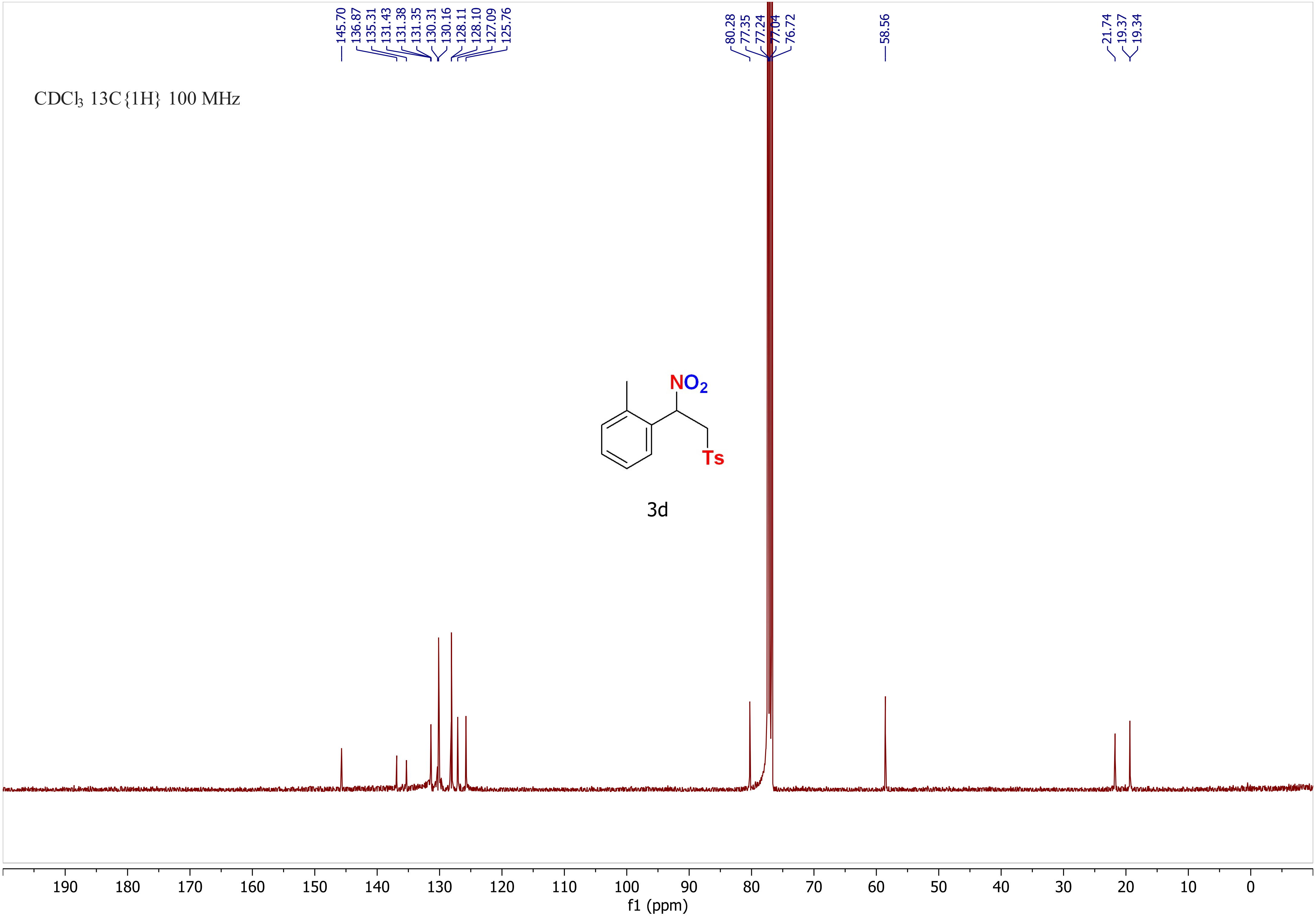

145.70
136.87
135.31
131.43
131.38
131.35
130.31
130.16
128.11
128.10
127.09
125.76
80.28
77.35
77.24
77.04
76.72
58.56
21.74
19.37
19.34
CDCl3 13C{1H} 100 MHz
NO2
Ts
3d
f1 (ppm)

CDCl3 400 MHz

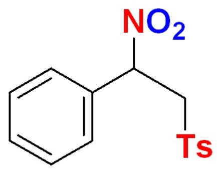

3e

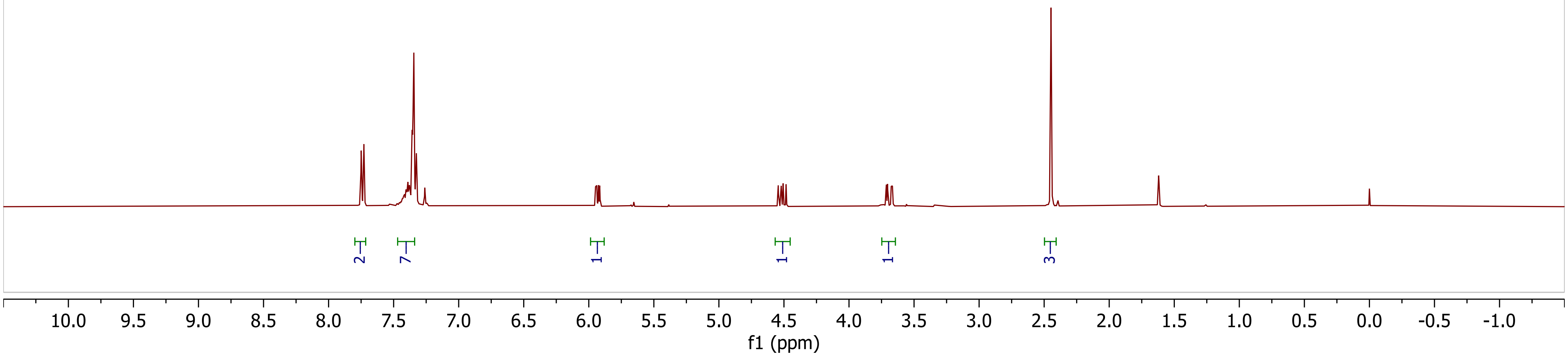

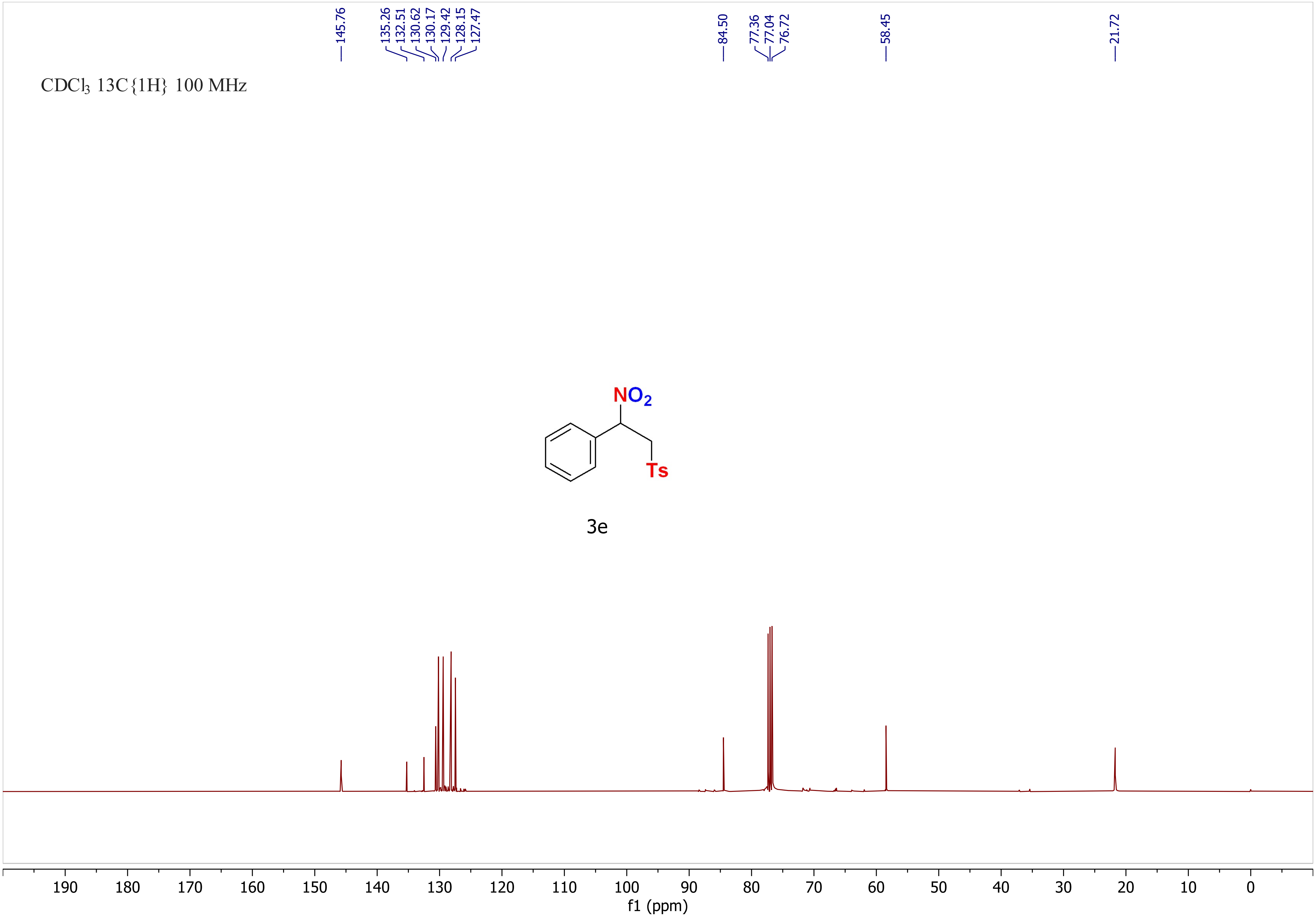
CDCl3 13C{1H} 100 MHz
145.76
135.26
132.51
130.62
130.17
129.42
128.15
127.47
84.50
77.36
77.04
76.72
58.45
21.72
NO2
Ts
3e
190 180 170 160 150 140 130 120 110 100 90 80 70 60 50 40 30 20 10 0
f1 (ppm)

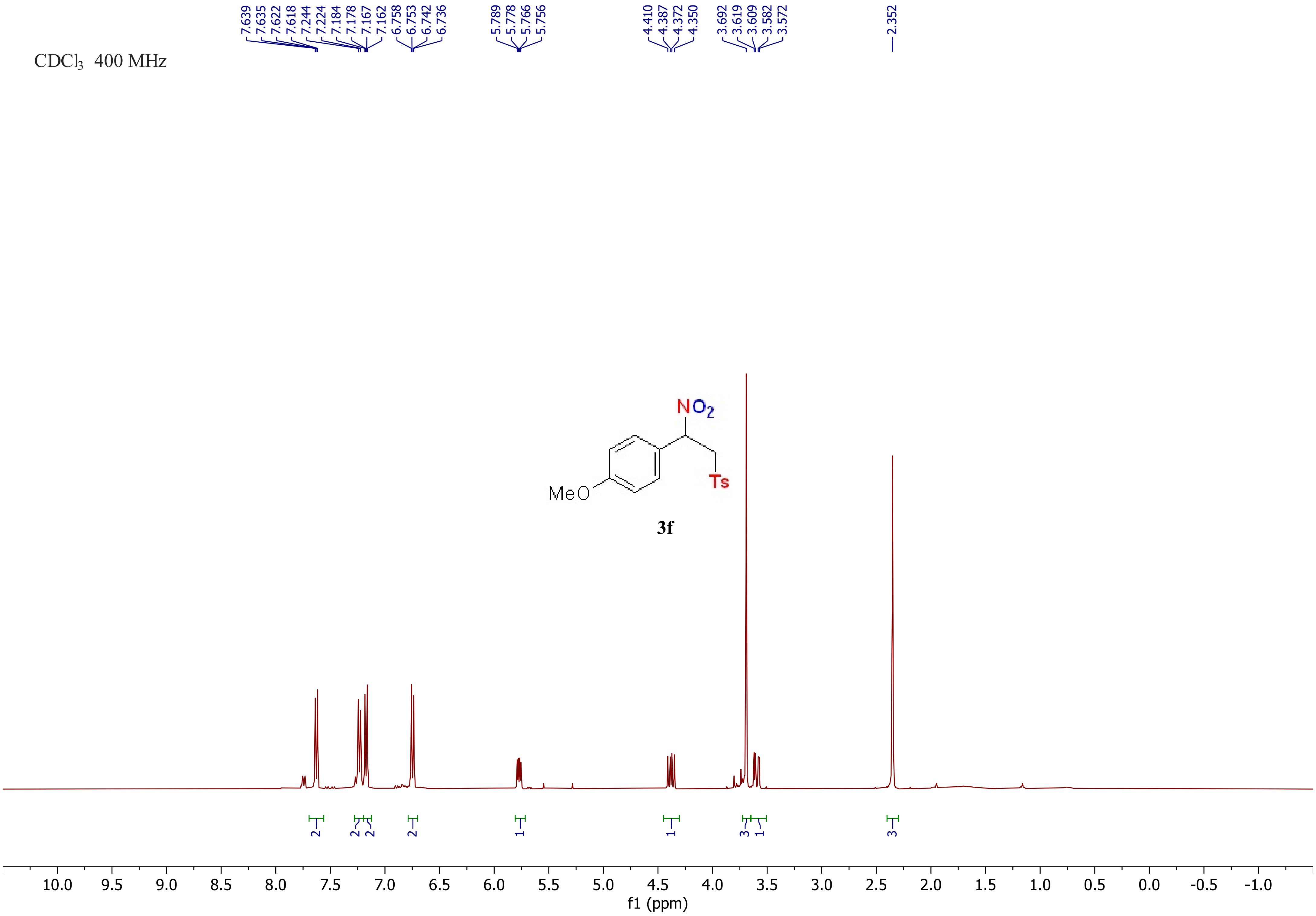
CDCl3 400 MHz
7.639
7.635
7.622
7.618
7.244
7.224
7.184
7.178
7.167
7.162
6.758
6.753
6.742
6.736
5.789
5.778
5.766
5.756
4.410
4.387
4.372
4.350
3.692
3.619
3.609
3.582
3.572
2.352
NO2
MeO
Ts
3f
2
2
2
2
1
1
3
1
3
10.0 9.5 9.0 8.5 8.0 7.5 7.0 6.5 6.0 5.5 5.0 4.5 4.0 3.5 3.0 2.5 2.0 1.5 1.0 0.5 0.0 -0.5 -1.0
f1 (ppm)

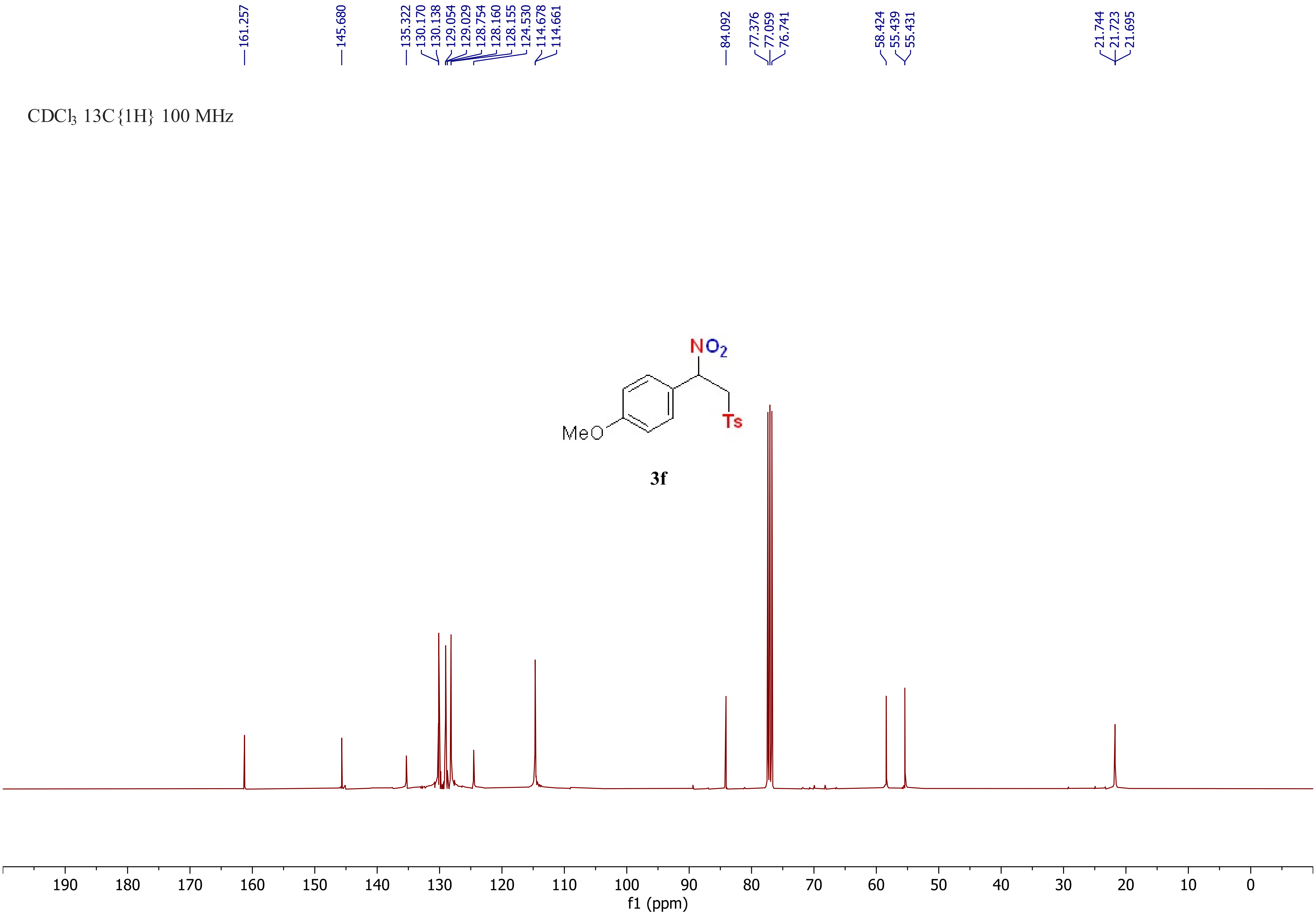
161.257
145.680
135.322
130.170
130.138
129.054
129.029
128.754
128.160
128.155
124.530
114.678
114.661
84.092
77.376
77.059
76.741
58.424
55.439
55.431
21.744
21.723
21.695
CDCl3 13C{1H} 100 MHz
NO2
Ts
MeO
3f
190 180 170 160 150 140 130 120 110 100 90 80 70 60 50 40 30 20 10 0
f1 (ppm)

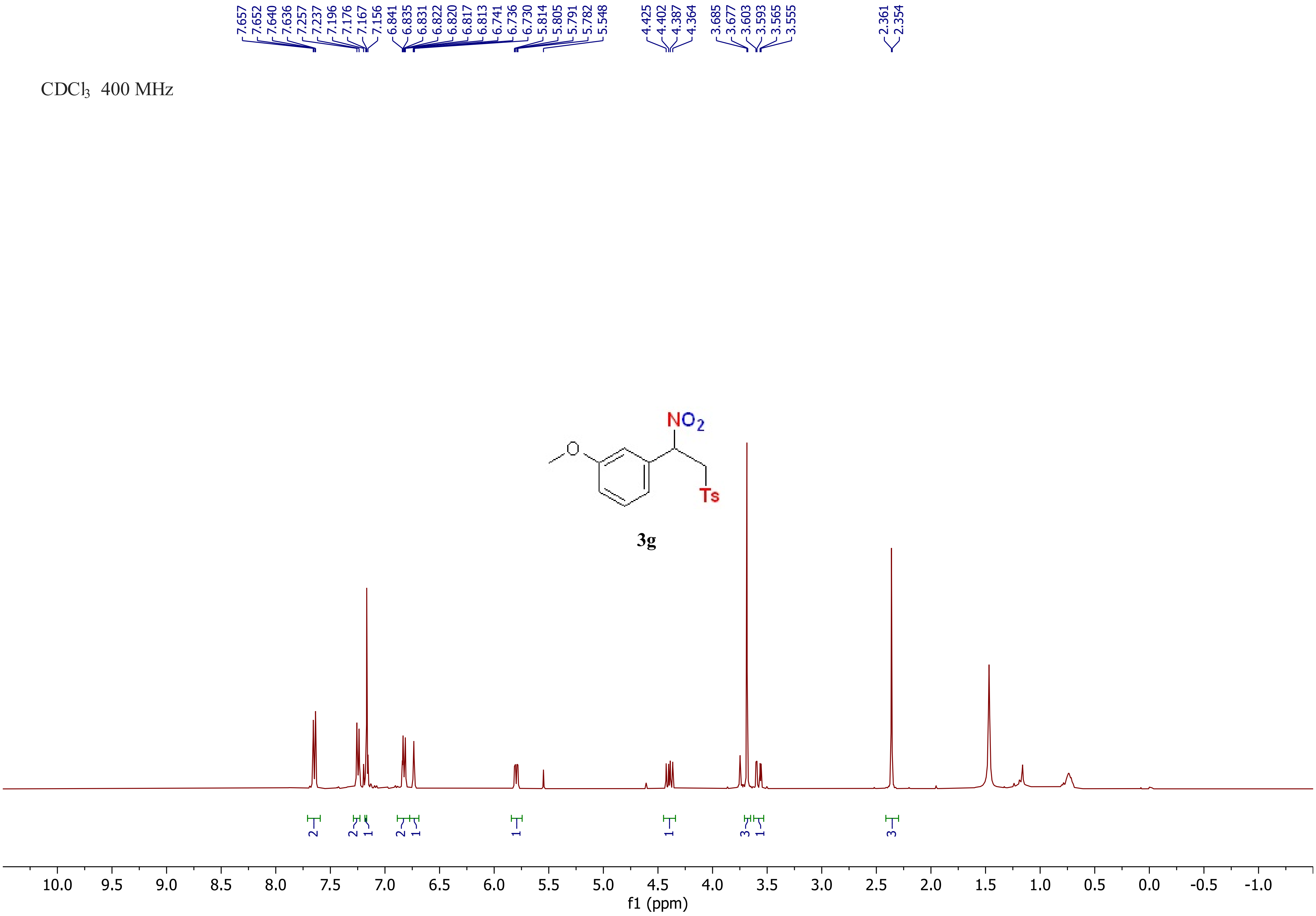

7.657
7.652
7.640
7.636
7.257
7.237
7.196
7.176
7.167
7.156
6.841
6.835
6.831
6.822
6.820
6.817
6.813
6.741
6.736
6.730
5.814
5.805
5.791
5.782
5.548
4.425
4.402
4.387
4.364
3.685
3.677
3.603
3.593
3.565
3.555
2.361
2.354
CDCl3 400 MHz
NO2
O
Ts
2
2
1
2
1
1
1
3
1
3
10.0 9.5 9.0 8.5 8.0 7.5 7.0 6.5 6.0 5.5 5.0 4.5 4.0 3.5 3.0 2.5 2.0 1.5 1.0 0.5 0.0 -0.5 -1.0
f1 (ppm)

3g

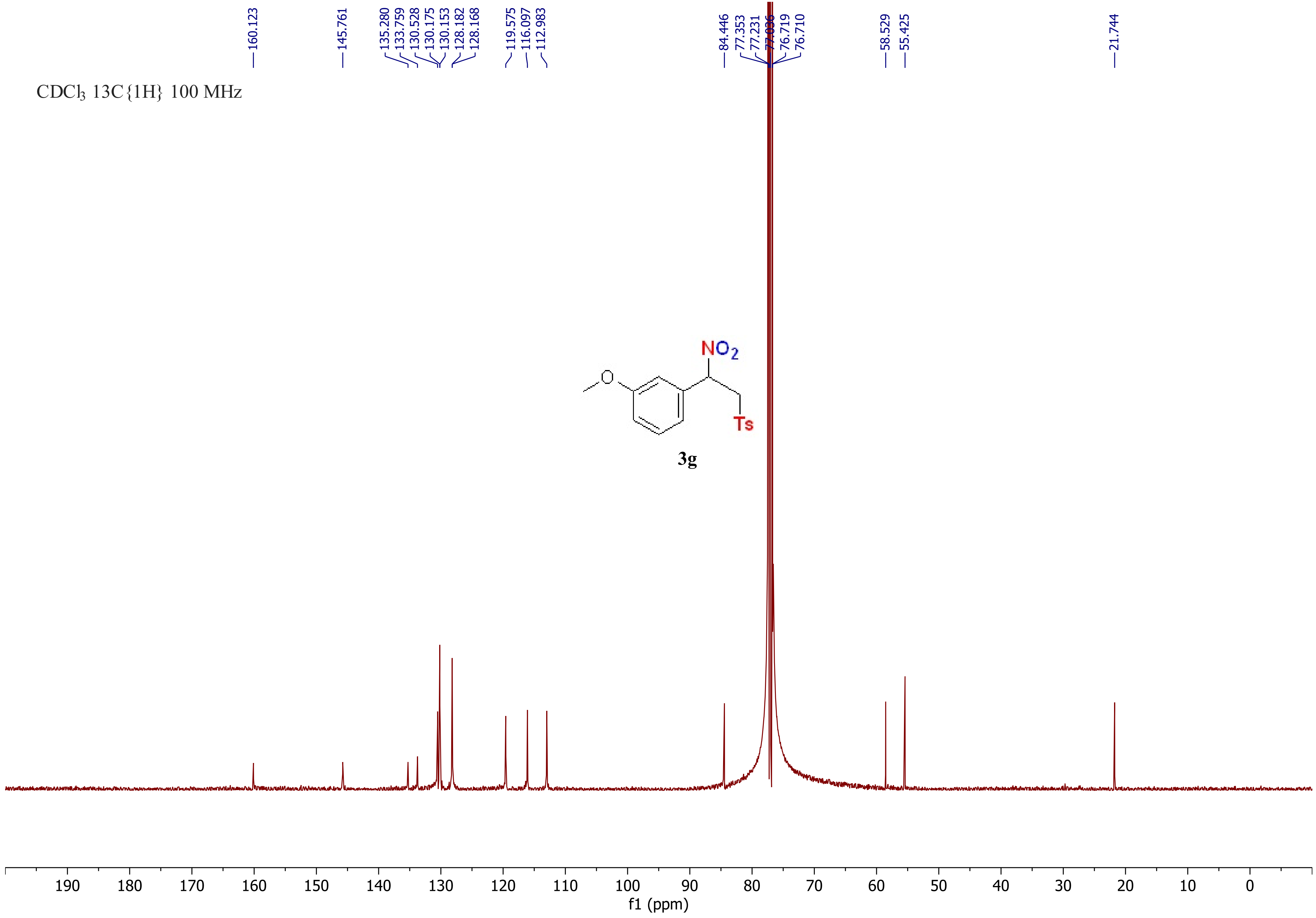
160.123
145.761
135.280
133.759
130.528
130.175
130.153
128.182
128.168
119.575
116.097
112.983
84.446
77.353
77.231
77.036
76.719
76.710
58.529
55.425
21.744
CDCl3 13C{1H} 100 MHz
NO2
O
Ts
3g
190 180 170 160 150 140 130 120 110 100 90 80 70 60 50 40 30 20 10 0
f1 (ppm)

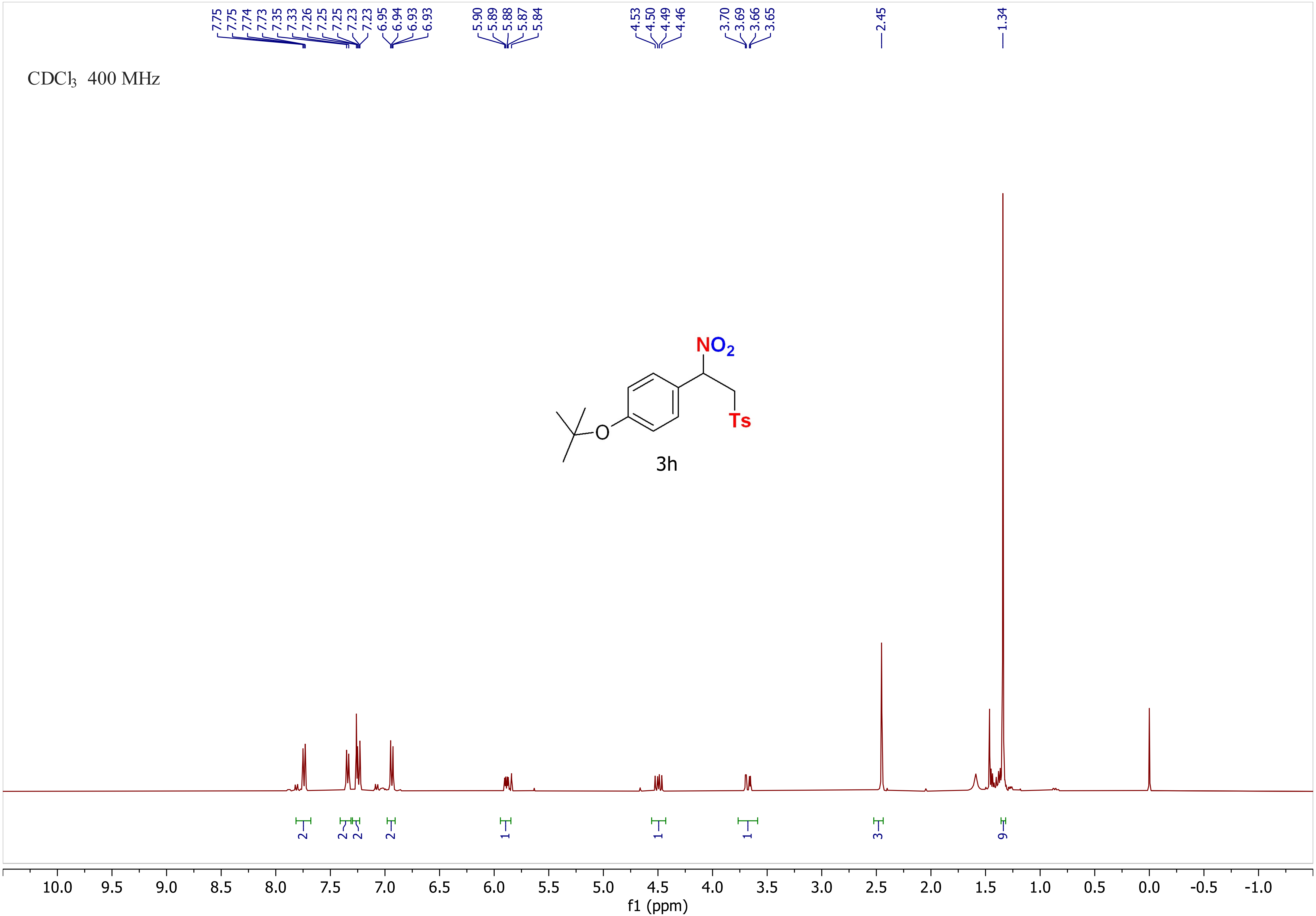

7.75
7.75
7.74
7.73
7.35
7.33
7.26
7.25
7.25
7.23
7.23
6.95
6.94
6.93
6.93
5.90
5.89
5.88
5.87
5.84
4.53
4.50
4.49
4.46
3.70
3.69
3.66
3.65
2.45
1.34
CDCl3 400 MHz
NO2
Ts
O
3h
2
2
2
2
1
1
1
3
9
10.0 9.5 9.0 8.5 8.0 7.5 7.0 6.5 6.0 5.5 5.0 4.5 4.0 3.5 3.0 2.5 2.0 1.5 1.0 0.5 0.0 -0.5 -1.0
f1 (ppm)

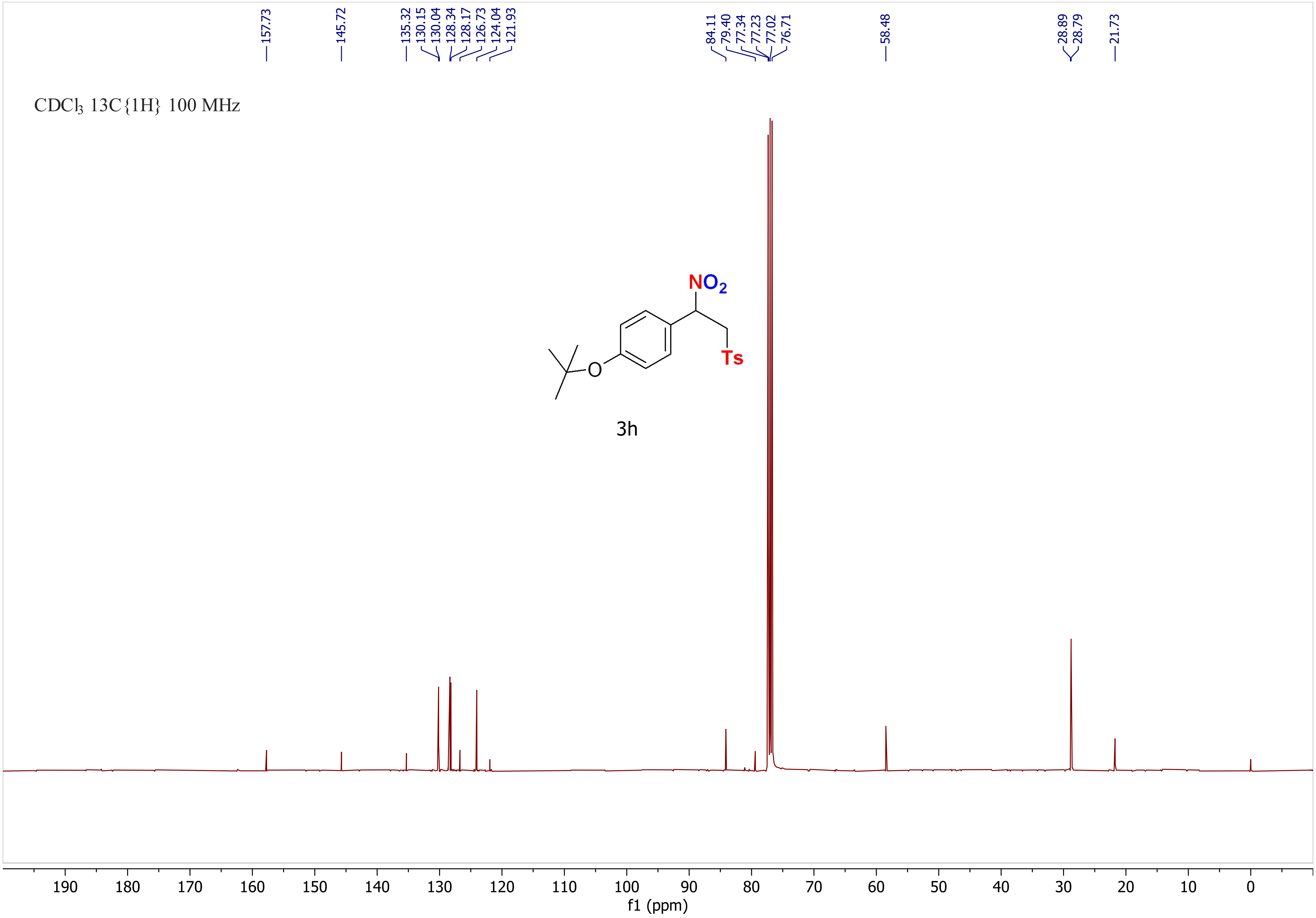

157.73
145.72
135.32
130.15
130.04
128.34
128.17
126.73
124.04
121.93
84.11
79.40
77.34
77.23
77.02
76.71
58.48
28.89
28.79
21.73
CDCl3 13C{1H} 100 MHz
NO2
O
Ts
3h
190 180 170 160 150 140 130 120 110 100 90 80 70 60 50 40 30 20 10 0
f1 (ppm)

CDCl$_3$ 400 MHz

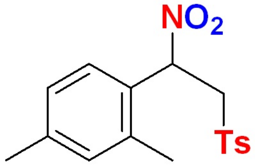

3i

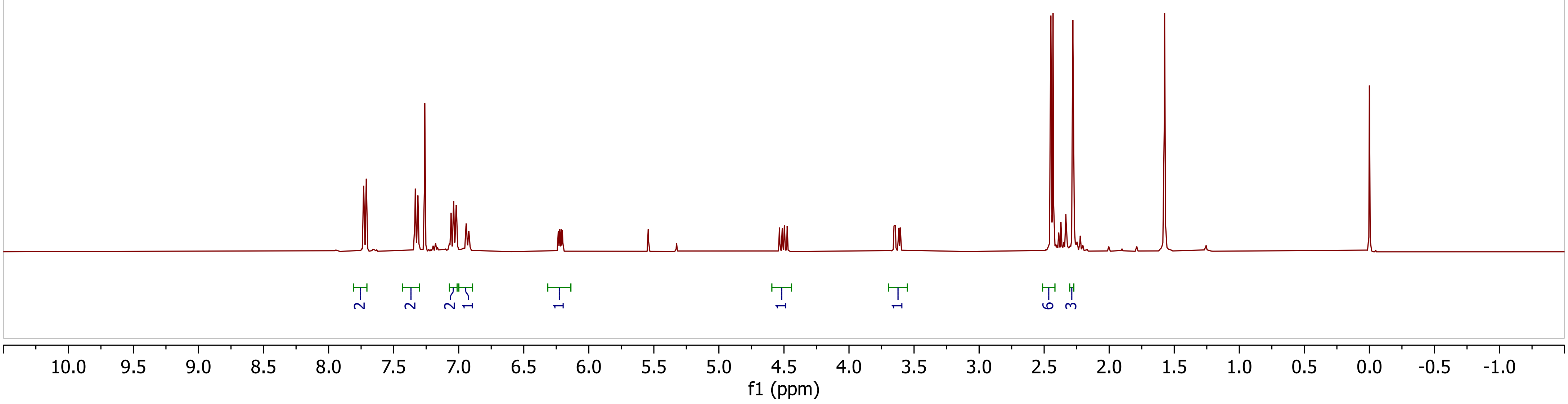

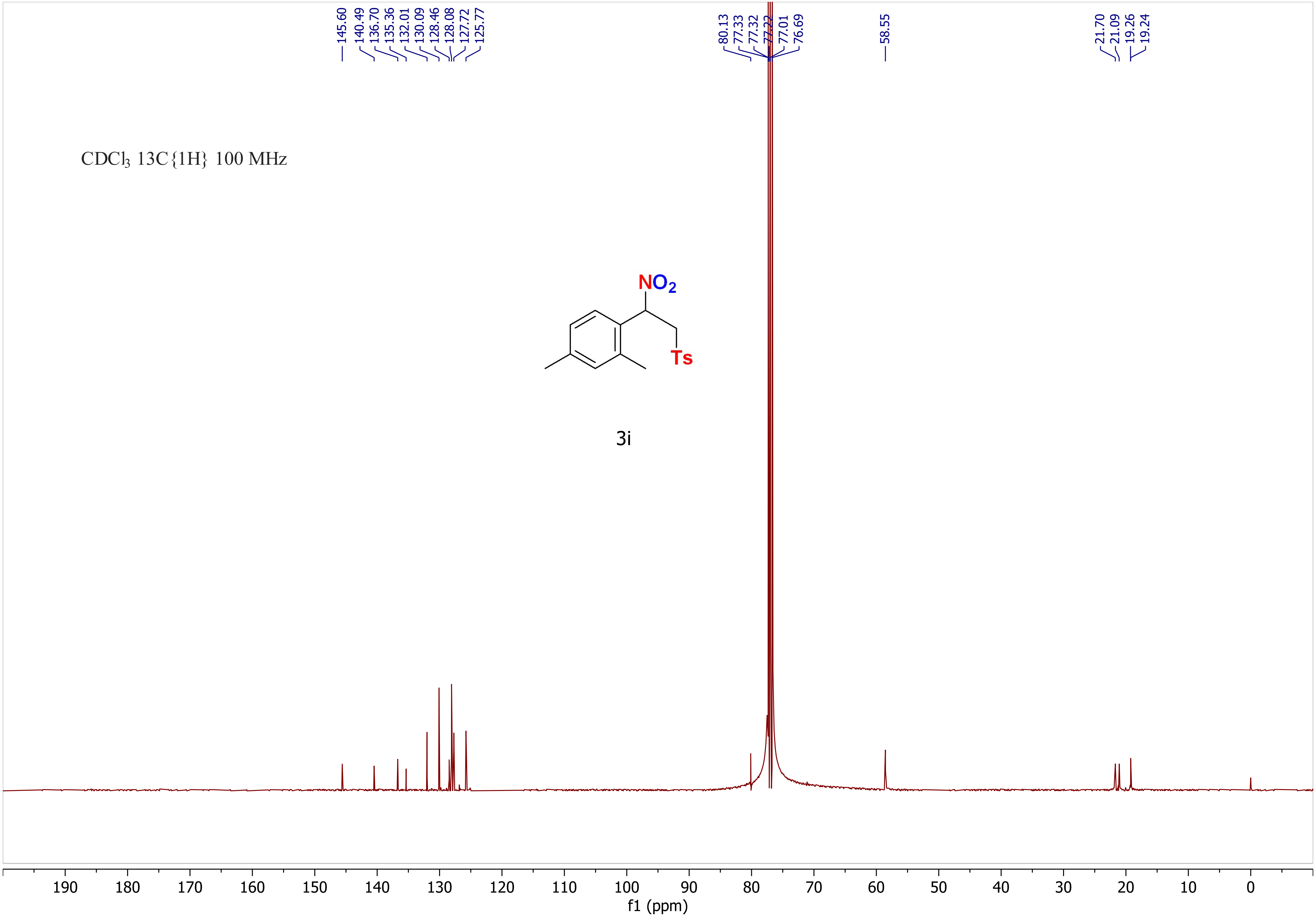

145.60
140.49
136.70
135.36
132.01
130.09
128.46
128.08
127.72
125.77
80.13
77.33
77.32
77.22
77.01
76.69
58.55
21.70
21.09
19.26
19.24
CDCl3 13C{1H} 100 MHz
NO2
Ts
3i
190 180 170 160 150 140 130 120 110 100 90 80 70 60 50 40 30 20 10 0
f1 (ppm)

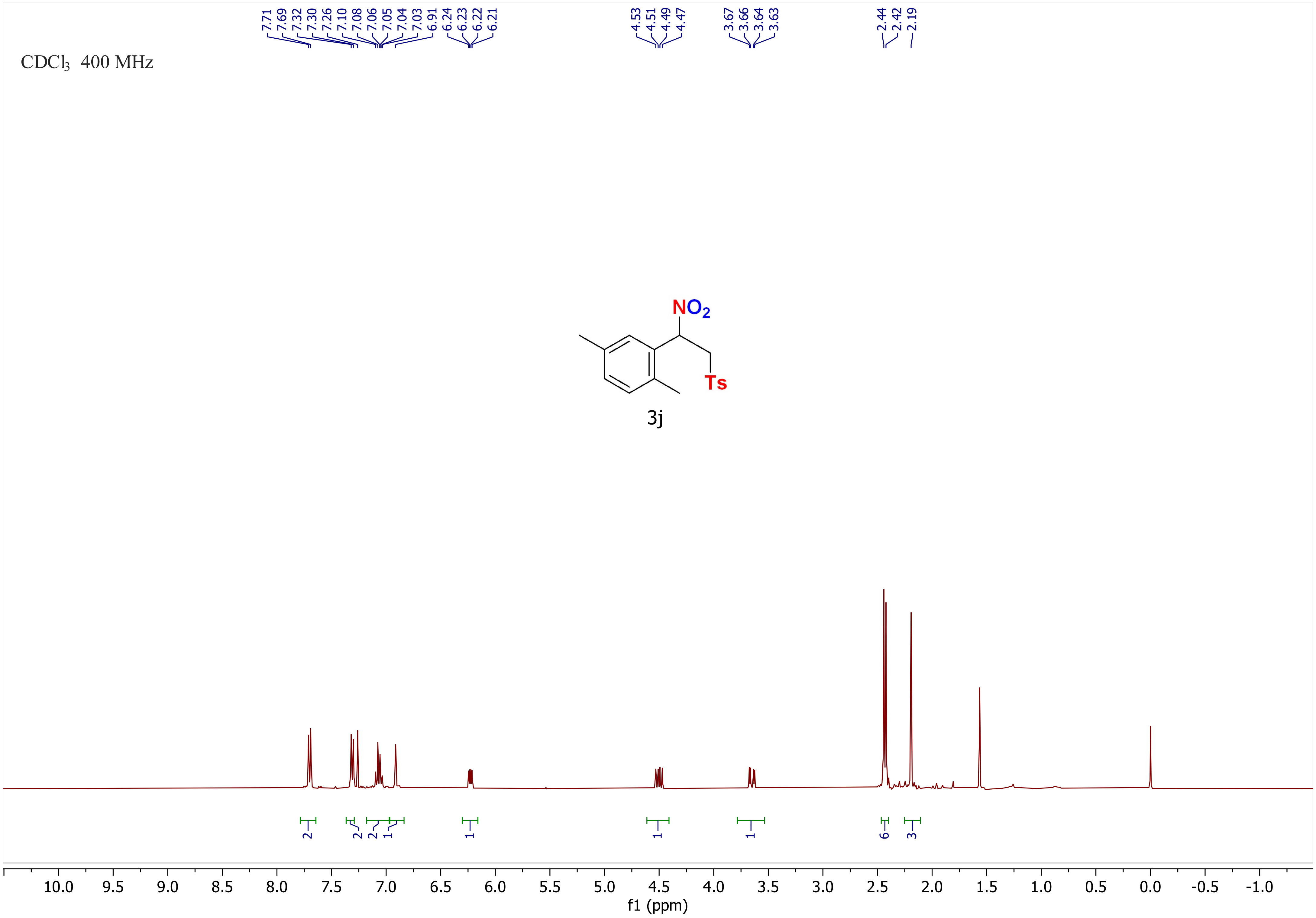
CDCl$_3$ 400 MHz
7.71 7.69 7.32 7.30 7.26 7.10 7.08 7.06 7.05 7.04 7.03 6.91 6.24 6.23 6.22 6.21
4.53 4.51 4.49 4.47
3.67 3.66 3.64 3.63
2.44 2.42 2.19
NO2
Ts
3j
2 2 2 1 1 1 1 6 3
10.0 9.5 9.0 8.5 8.0 7.5 7.0 6.5 6.0 5.5 5.0 4.5 4.0 3.5 3.0 2.5 2.0 1.5 1.0 0.5 0.0 -0.5 -1.0
f1 (ppm)

CDCl$_3$ 13C{1H} 100 MHz

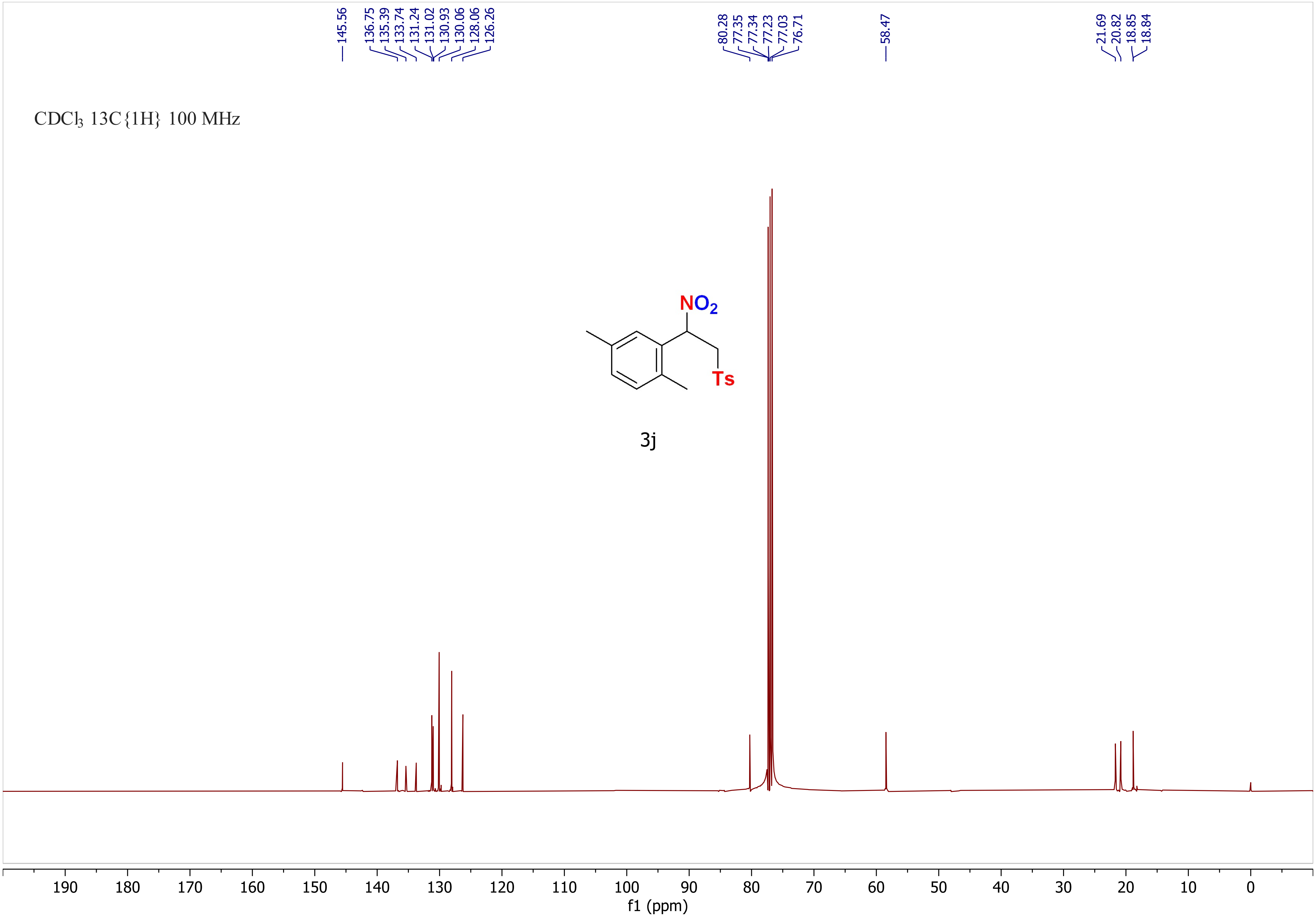

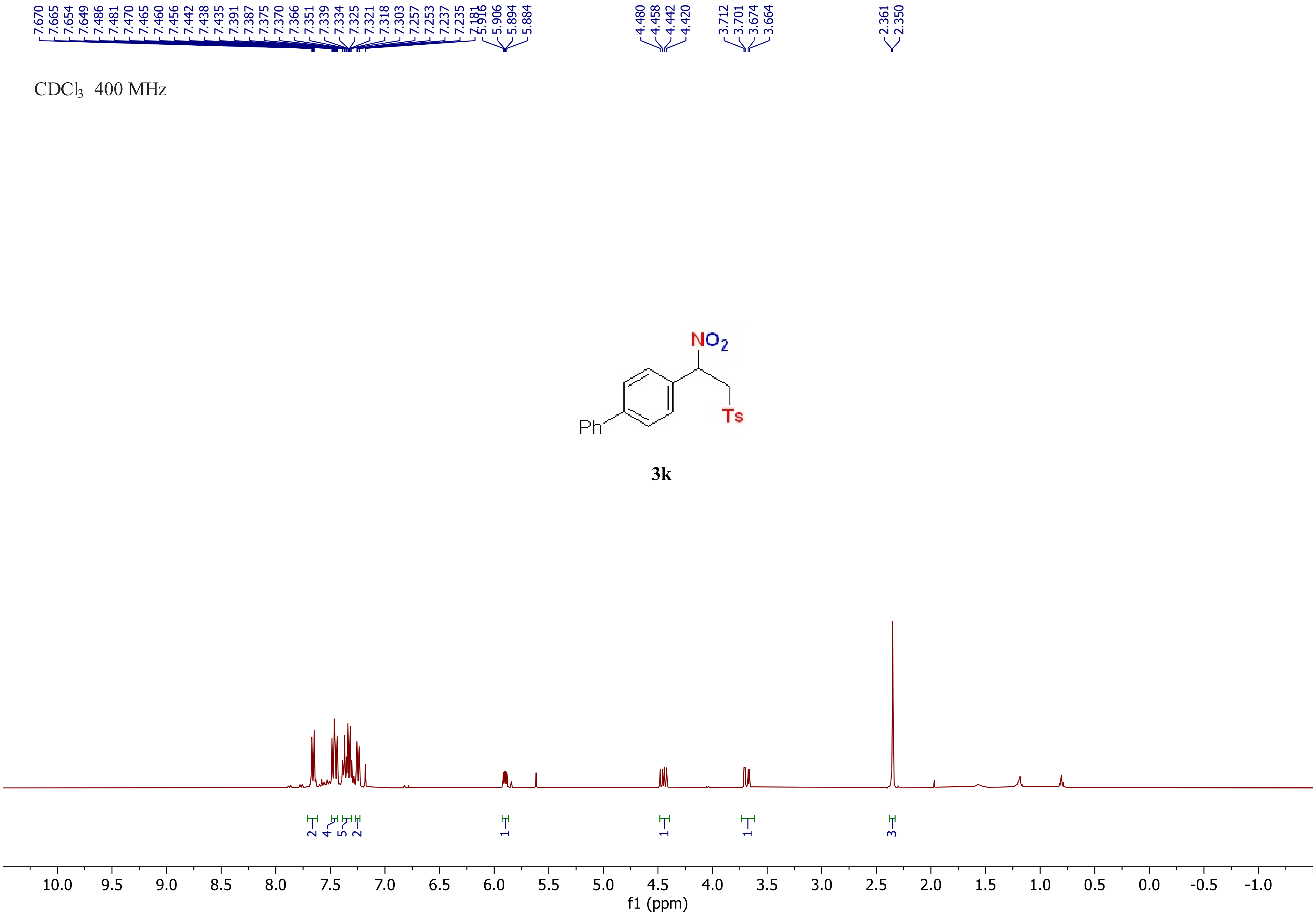
7.670
7.665
7.654
7.649
7.486
7.481
7.470
7.465
7.460
7.456
7.442
7.438
7.435
7.391
7.387
7.375
7.370
7.366
7.351
7.339
7.334
7.325
7.321
7.318
7.303
7.257
7.253
7.237
7.235
7.181
5.916
5.906
5.894
5.884
4.480
4.458
4.442
4.420
3.712
3.701
3.674
3.664
2.361
2.350
CDCl3 400 MHz
NO2
Ph
Ts
2
4
5
2
1
1
1
3
10.0
9.5
9.0
8.5
8.0
7.5
7.0
6.5
6.0
5.5
5.0
4.5
4.0
3.5
3.0
2.5
2.0
1.5
1.0
0.5
0.0
-0.5
-1.0
f1 (ppm)

3k

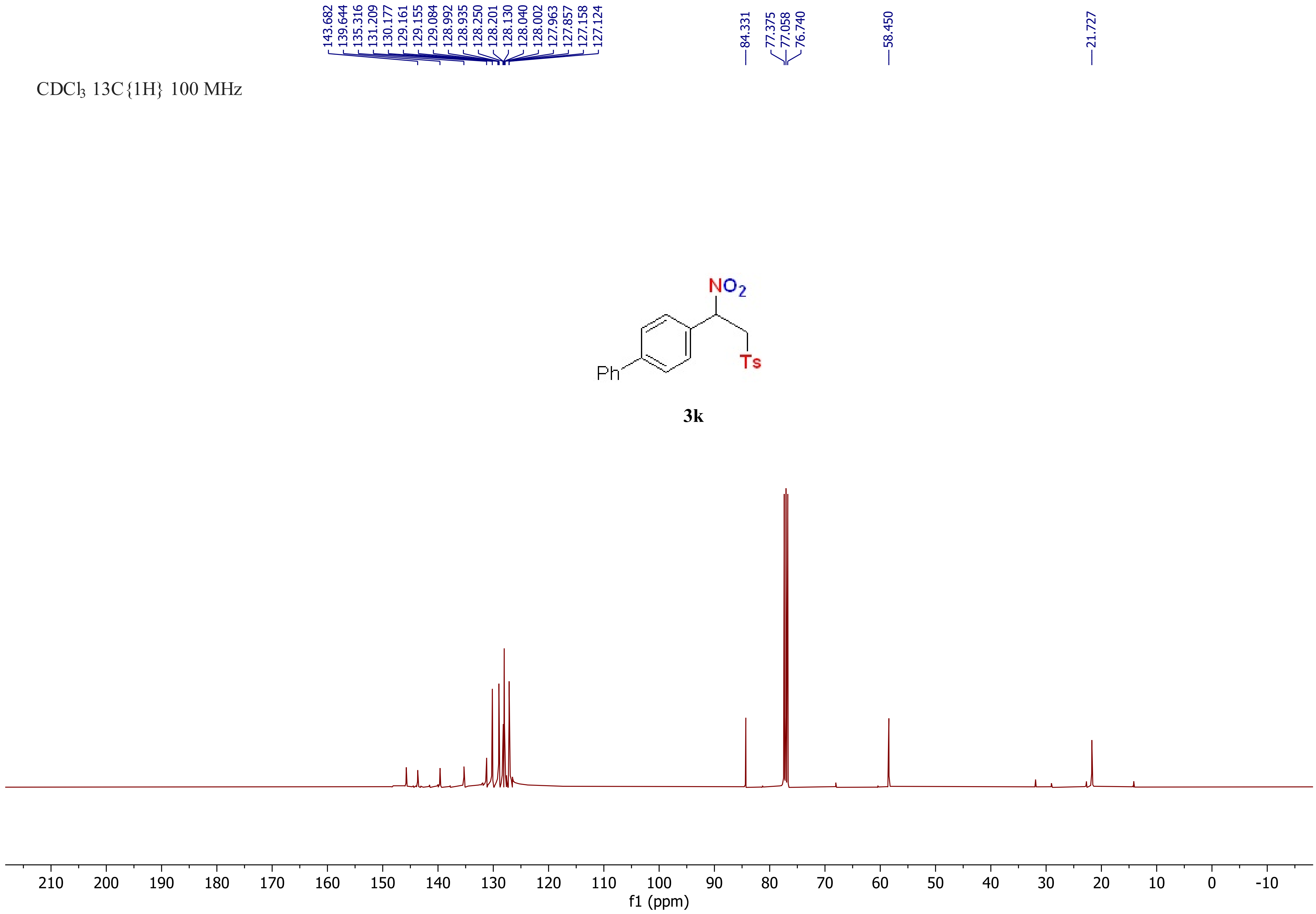
143.682
139.644
135.316
131.209
130.177
129.161
129.155
129.084
128.992
128.935
128.250
128.201
128.130
128.040
128.002
127.963
127.857
127.158
127.124
84.331
77.375
77.058
76.740
58.450
21.727
CDCl$_3$ 13C{1H} 100 MHz
NO$_2$
Ts
Ph
3k
210 200 190 180 170 160 150 140 130 120 110 100 90 80 70 60 50 40 30 20 10 0 -10
f1 (ppm)

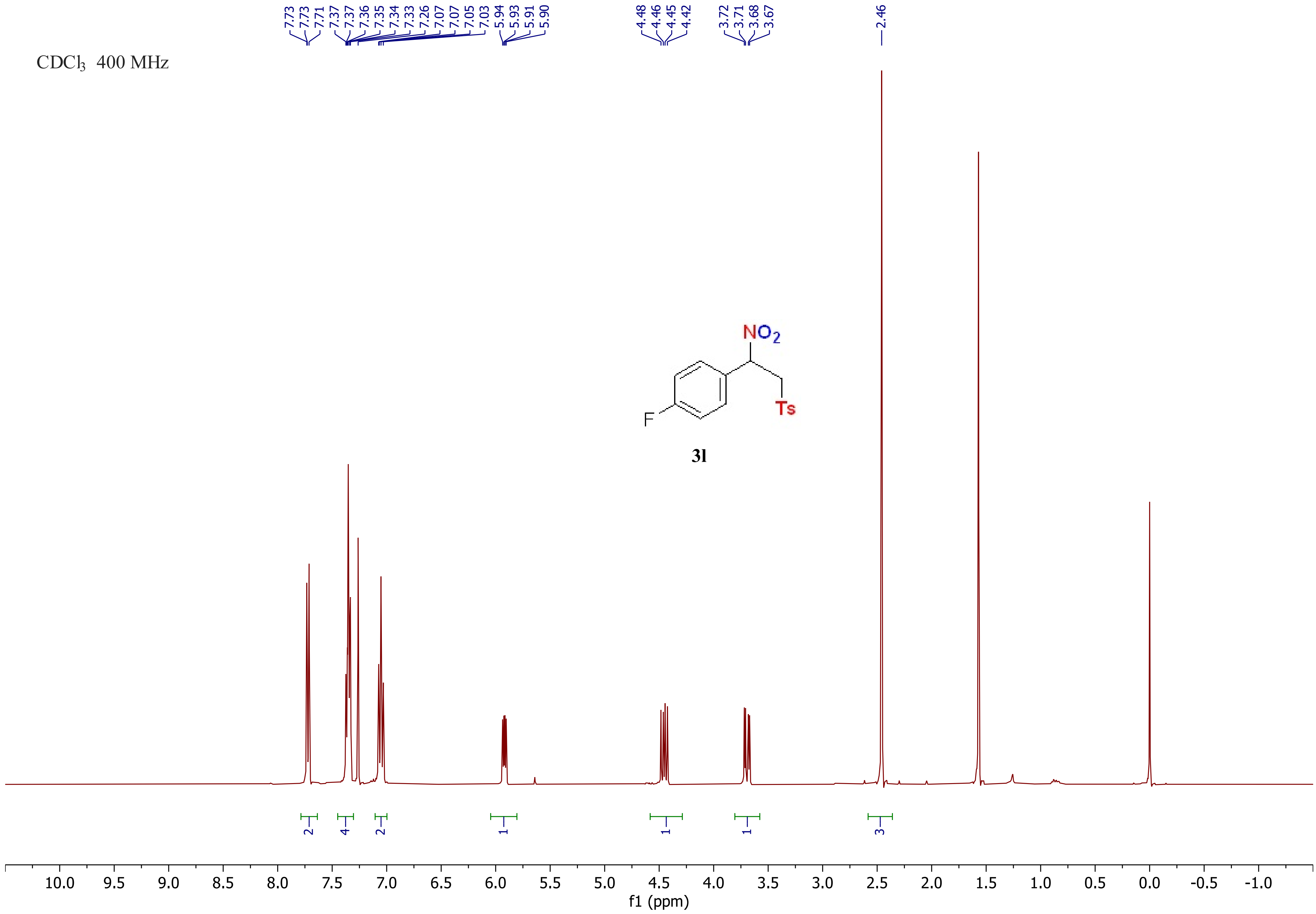
CDCl$_3$ 400 MHz
7.73
7.73
7.71
7.37
7.37
7.36
7.35
7.34
7.33
7.26
7.07
7.07
7.05
7.03
5.94
5.93
5.91
5.90
4.48
4.46
4.45
4.42
3.72
3.71
3.68
3.67
2.46
NO2
F
Ts
3l
2
4
2
1
1
1
3
10.0 9.5 9.0 8.5 8.0 7.5 7.0 6.5 6.0 5.5 5.0 4.5 4.0 3.5 3.0 2.5 2.0 1.5 1.0 0.5 0.0 -0.5 -1.0
f1 (ppm)

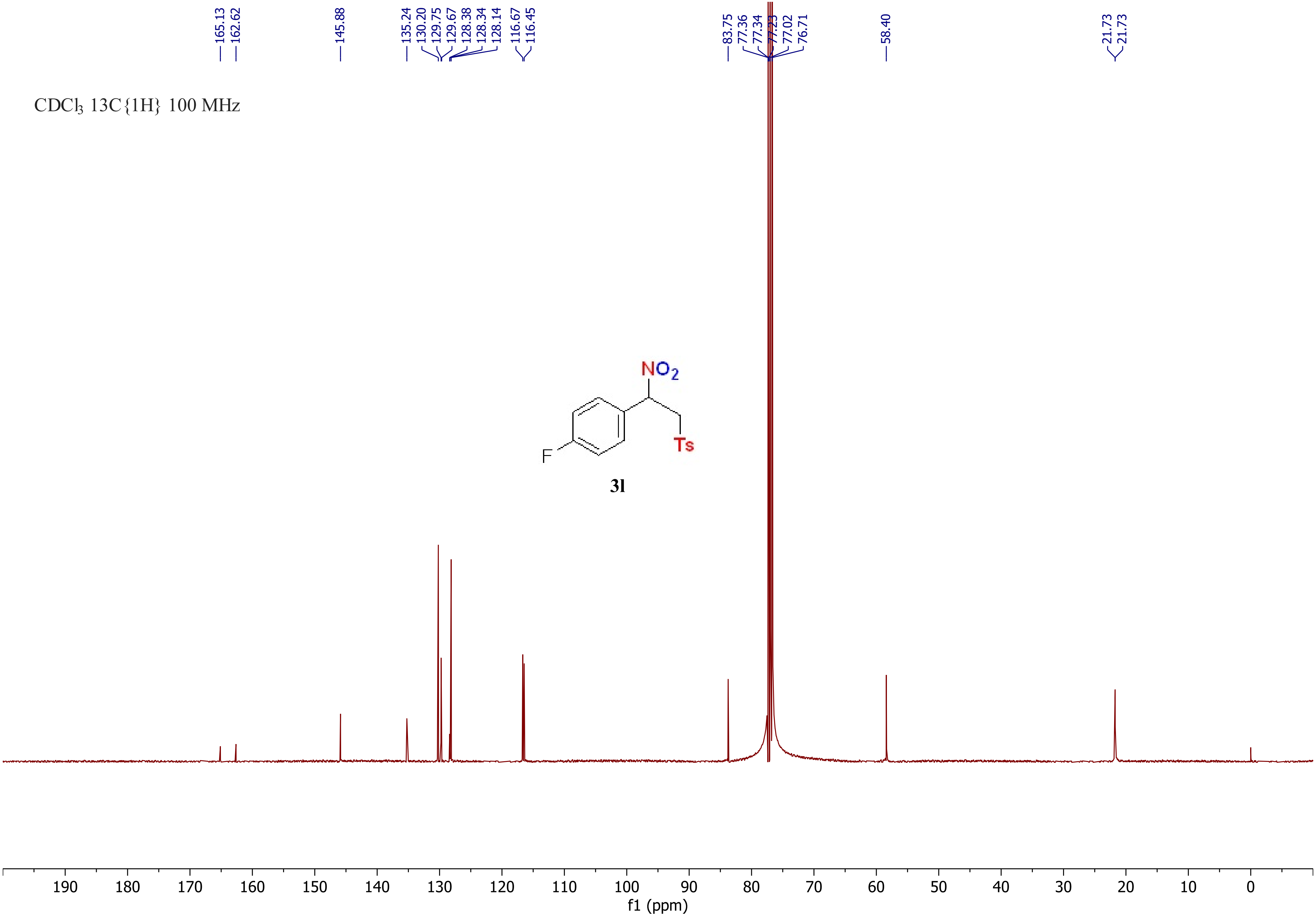

165.13
162.62
145.88
135.24
130.20
129.75
129.67
128.38
128.34
128.14
116.67
116.45
83.75
77.36
77.34
77.23
77.02
76.71
58.40
21.73
21.73
CDCl3 13C{1H} 100 MHz
NO2
F
Ts
3l
190 180 170 160 150 140 130 120 110 100 90 80 70 60 50 40 30 20 10 0
f1 (ppm)

CDCl$_3$ 400 MHz

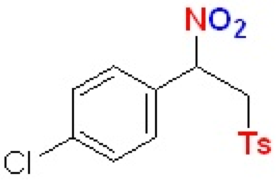

3m

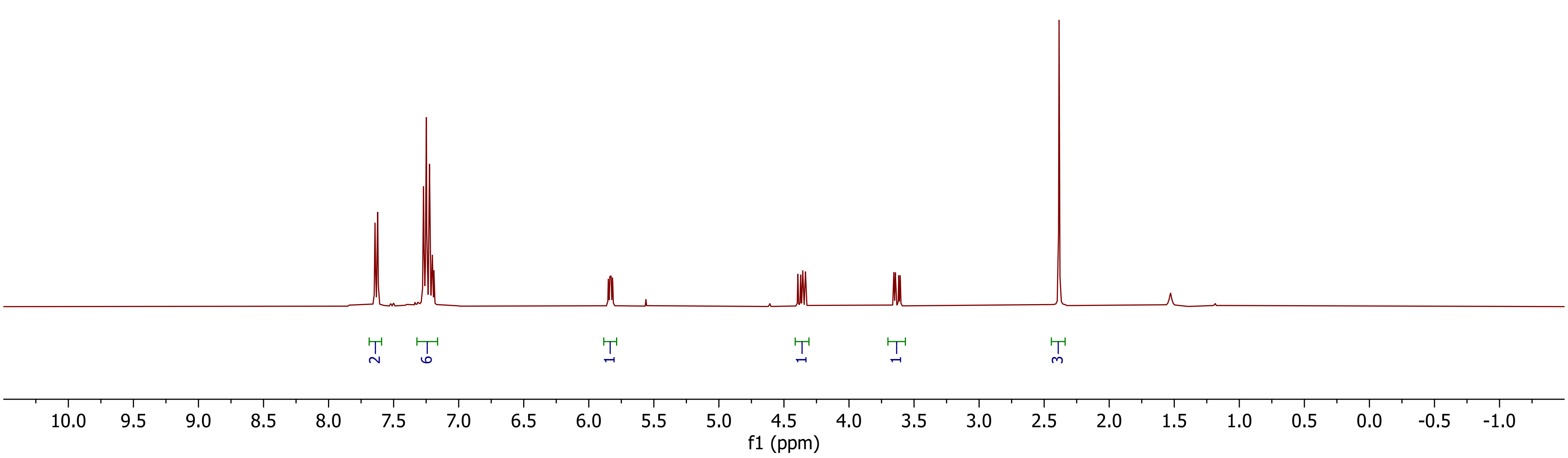

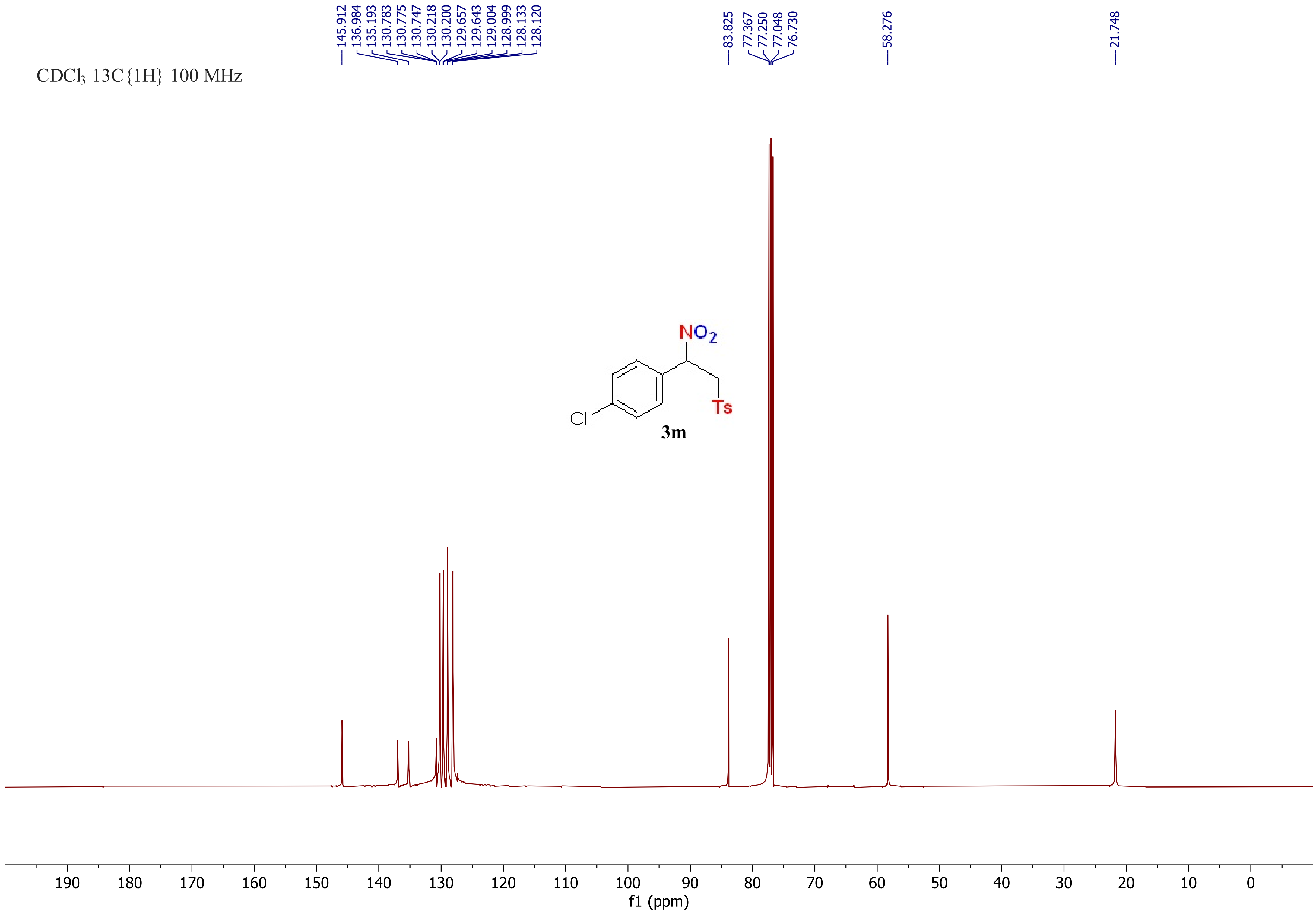
CDCl3 13C{1H} 100 MHz
145.912
136.984
135.193
130.783
130.775
130.747
130.218
130.200
129.657
129.643
129.004
128.999
128.133
128.120
83.825
77.367
77.250
77.048
76.730
58.276
21.748
NO2
Cl
Ts
3m
190
180
170
160
150
140
130
120
110
100
90
80
70
60
50
40
30
20
10
0
f1 (ppm)

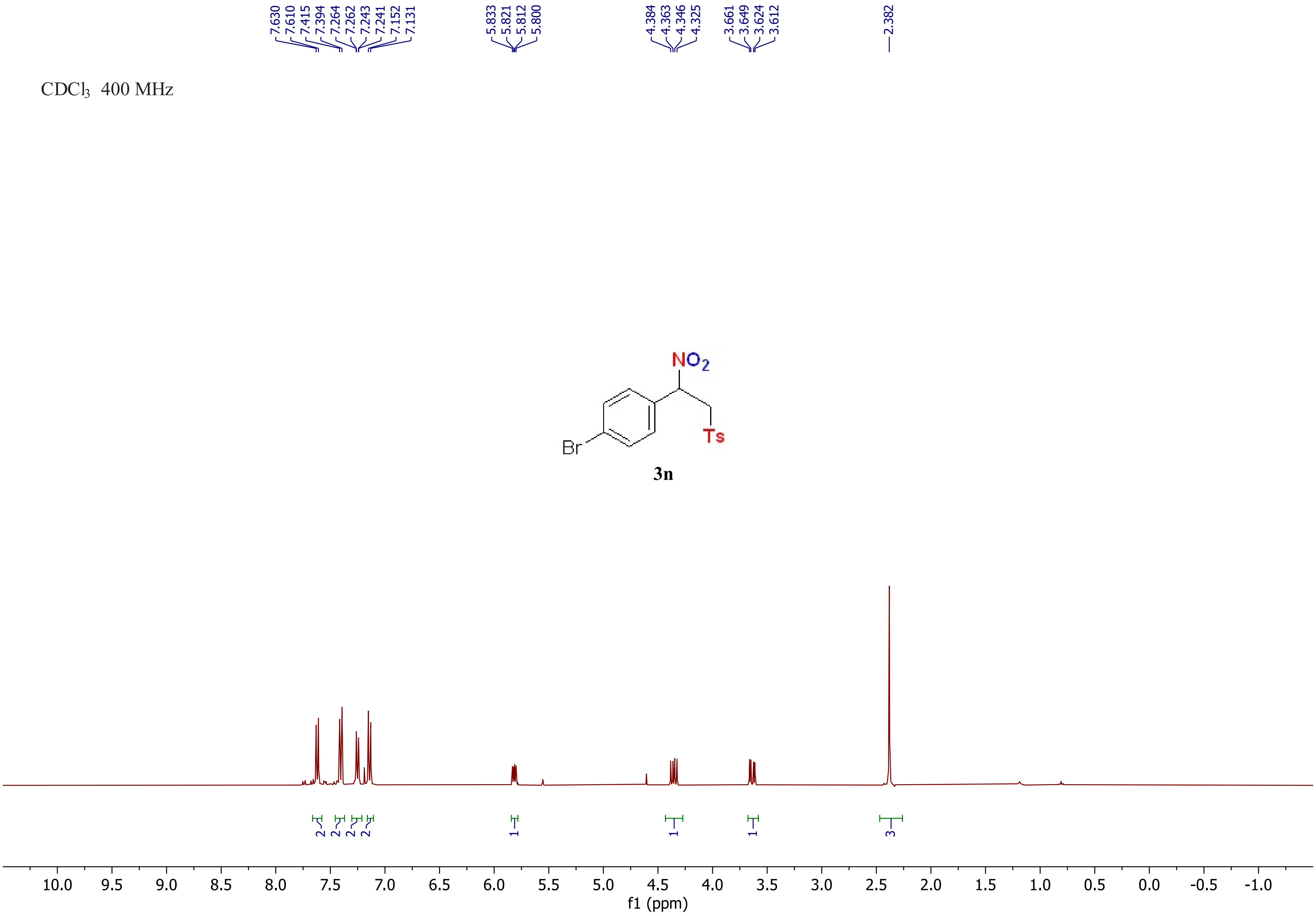

7.630
7.610
7.415
7.394
7.264
7.262
7.243
7.241
7.152
7.131
5.833
5.821
5.812
5.800
4.384
4.363
4.346
4.325
3.661
3.649
3.624
3.612
2.382
CDCl3 400 MHz
NO2
Br
Ts
2
2
2
2
1
1
1
3
10.0 9.5 9.0 8.5 8.0 7.5 7.0 6.5 6.0 5.5 5.0 4.5 4.0 3.5 3.0 2.5 2.0 1.5 1.0 0.5 0.0 -0.5 -1.0
f1 (ppm)

3n

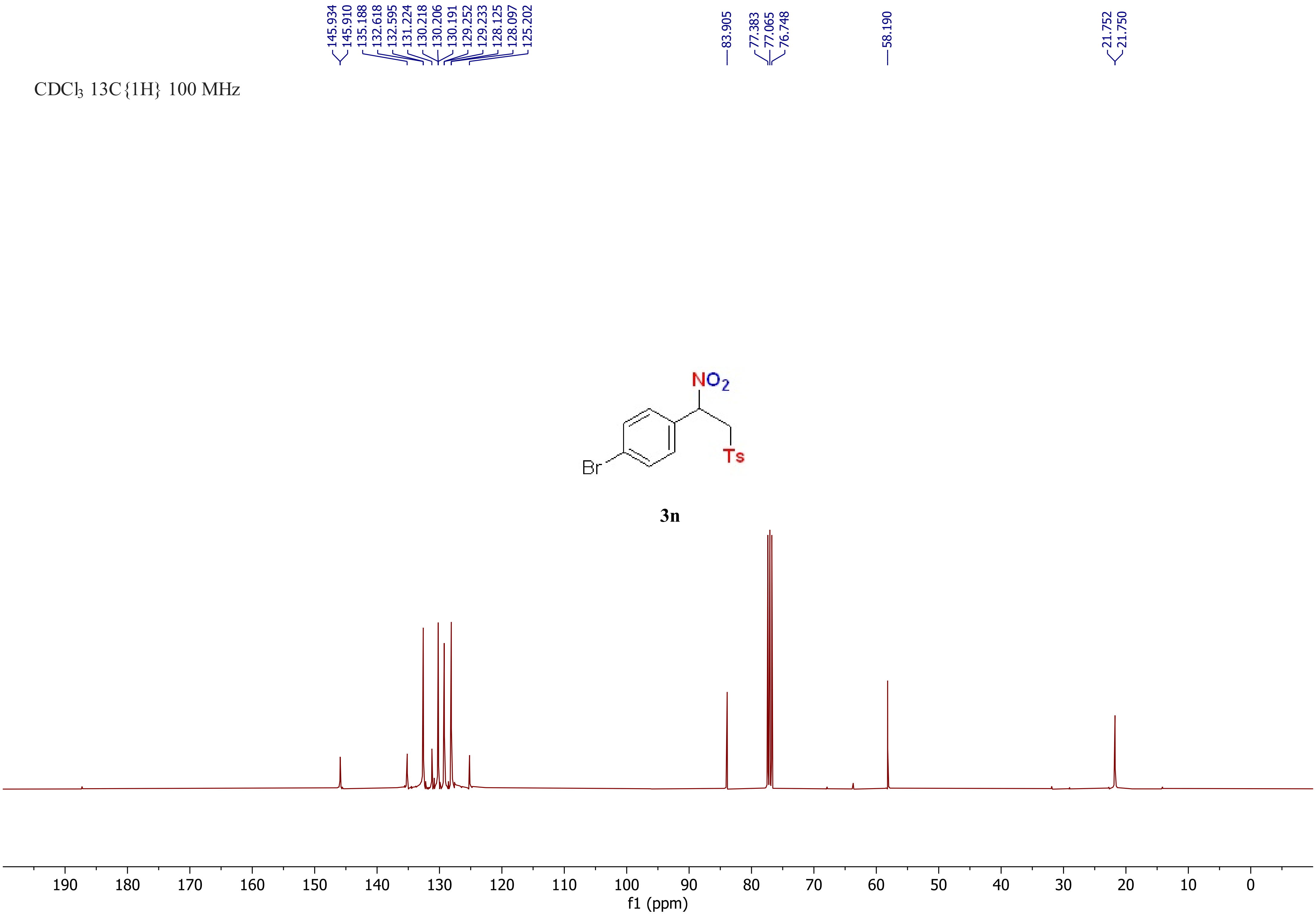

145.934
145.910
135.188
132.618
132.595
131.224
130.218
130.206
130.191
129.252
129.233
128.125
128.097
125.202
83.905
77.383
77.065
76.748
58.190
21.752
21.750
$CDCl_3$ 13C{1H} 100 MHz
NO_2
Br
Ts
3n
190 180 170 160 150 140 130 120 110 100 90 80 70 60 50 40 30 20 10 0
f1 (ppm)

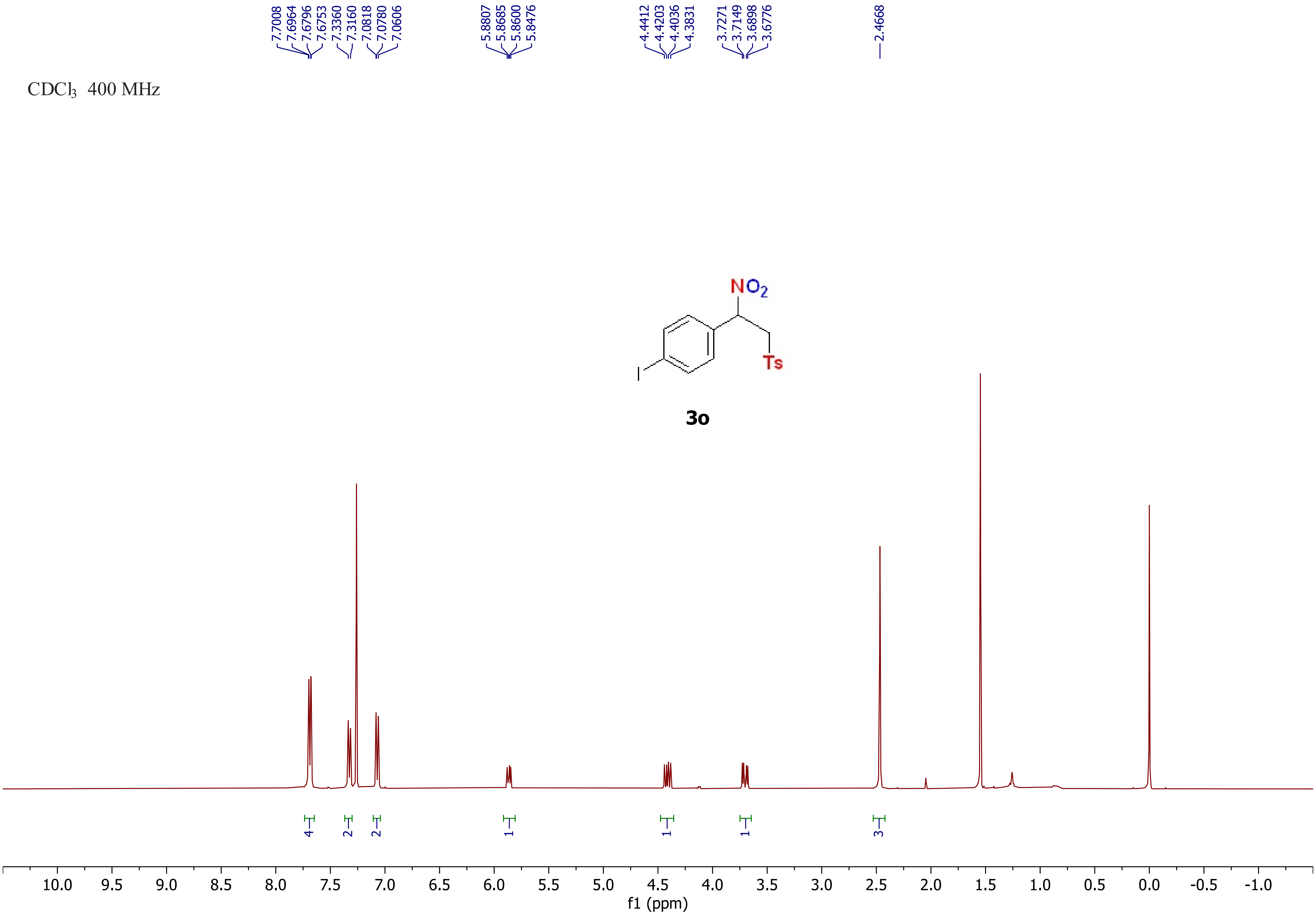
7.7008
7.6964
7.6796
7.6753
7.3360
7.3160
7.0818
7.0780
7.0606
5.8807
5.8685
5.8600
5.8476
4.4412
4.4203
4.4036
4.3831
3.7271
3.7149
3.6898
3.6776
2.4668
CDCl3 400 MHz
NO2
I
Ts
3o
4
2
2
1
1
1
3
10.0
9.5
9.0
8.5
8.0
7.5
7.0
6.5
6.0
5.5
5.0
4.5
4.0
3.5
3.0
2.5
2.0
1.5
1.0
0.5
0.0
-0.5
-1.0
f1 (ppm)

CDCl3 13C{1H} 100 MHz

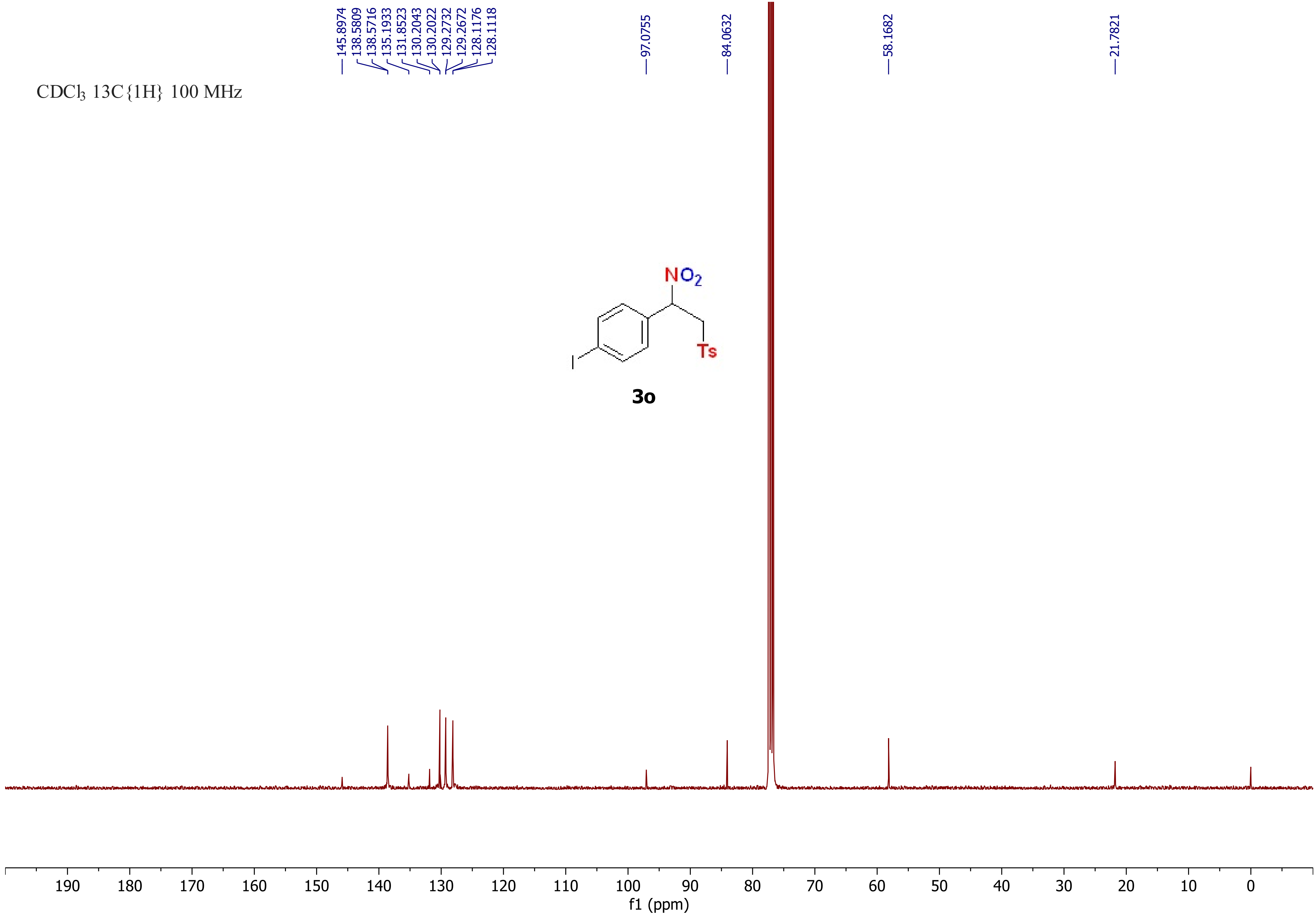

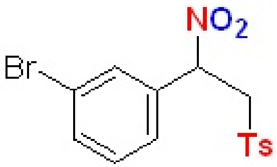
NO2
Br
Ts

3p

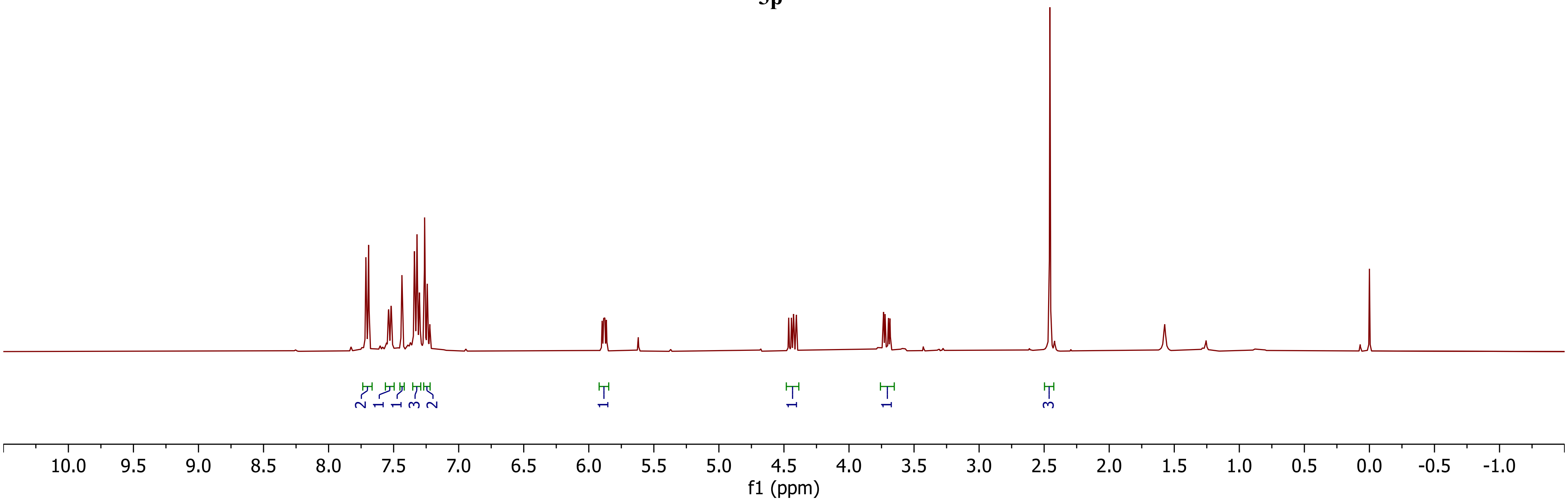
CDCl3 400 MHz
7.7129
7.6926
7.5430
7.5402
7.5377
7.5354
7.5233
7.5205
7.5180
7.5158
7.4413
7.4364
7.4316
7.3401
7.3202
7.3052
7.3019
7.2981
7.2617
7.2590
7.2417
7.2220
5.8975
5.8860
5.8765
5.8648
4.4632
4.4422
4.4258
4.4046
3.7344
3.7227
3.6969
3.6850
2.4556
2
1
1
3
2
1
1
1
3
10.0
9.5
9.0
8.5
8.0
7.5
7.0
6.5
6.0
5.5
5.0
4.5
4.0
3.5
3.0
2.5
2.0
1.5
1.0
0.5
0.0
-0.5
-1.0
f1 (ppm)

CDCl$_3$ 13C{1H} 100 MHz

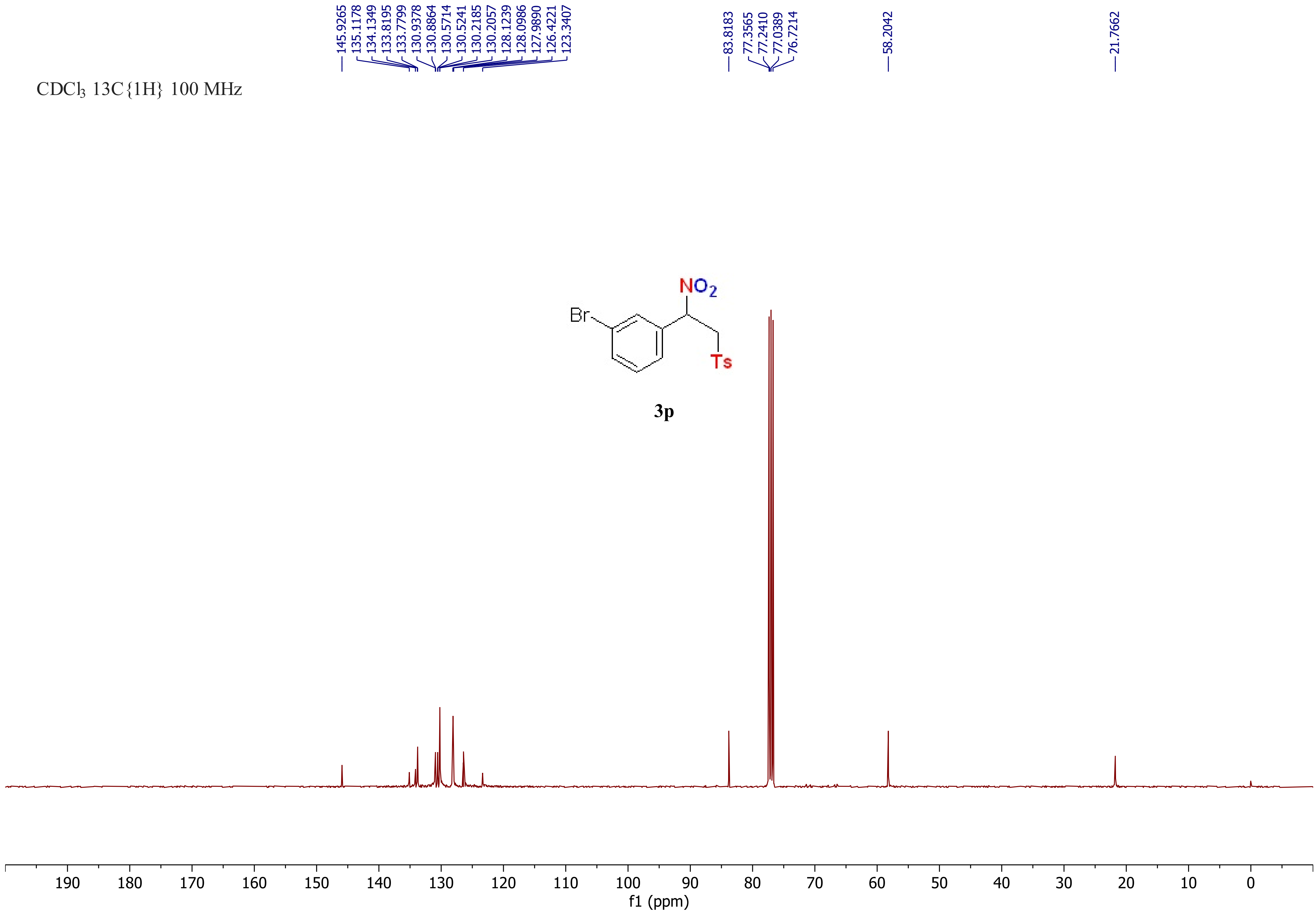

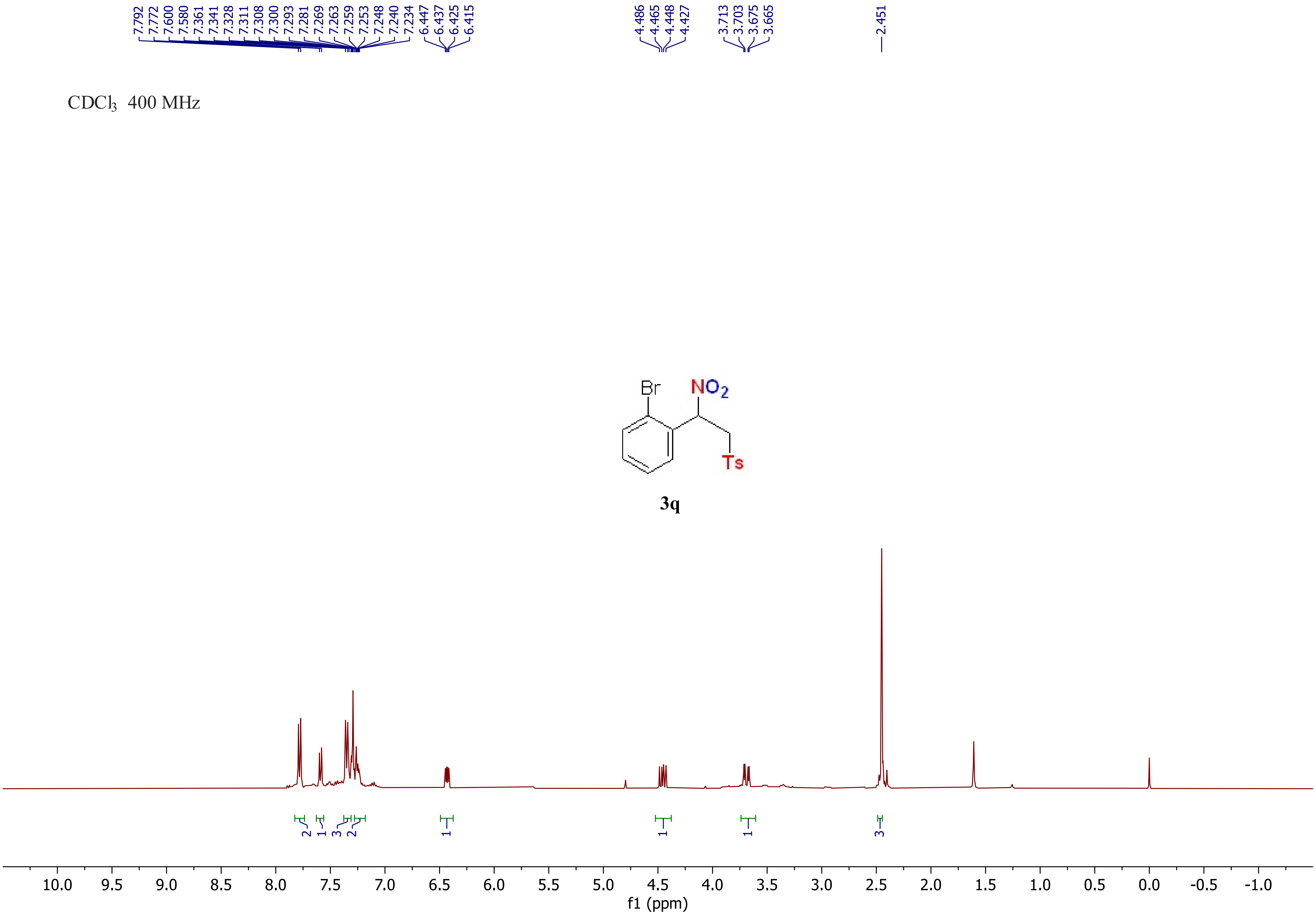

7.792
7.772
7.600
7.580
7.361
7.341
7.328
7.311
7.308
7.300
7.293
7.281
7.269
7.263
7.259
7.253
7.248
7.240
7.234
6.447
6.437
6.425
6.415
4.486
4.465
4.448
4.427
3.713
3.703
3.675
3.665
2.451
CDCl3 400 MHz
Br
NO2
Ts
3q
2
1
3
2
1
1
1
3
10.0 9.5 9.0 8.5 8.0 7.5 7.0 6.5 6.0 5.5 5.0 4.5 4.0 3.5 3.0 2.5 2.0 1.5 1.0 0.5 0.0 -0.5 -1.0
f1 (ppm)

$CDCl_3$ 13C{1H} 100 MHz

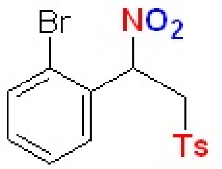

3q

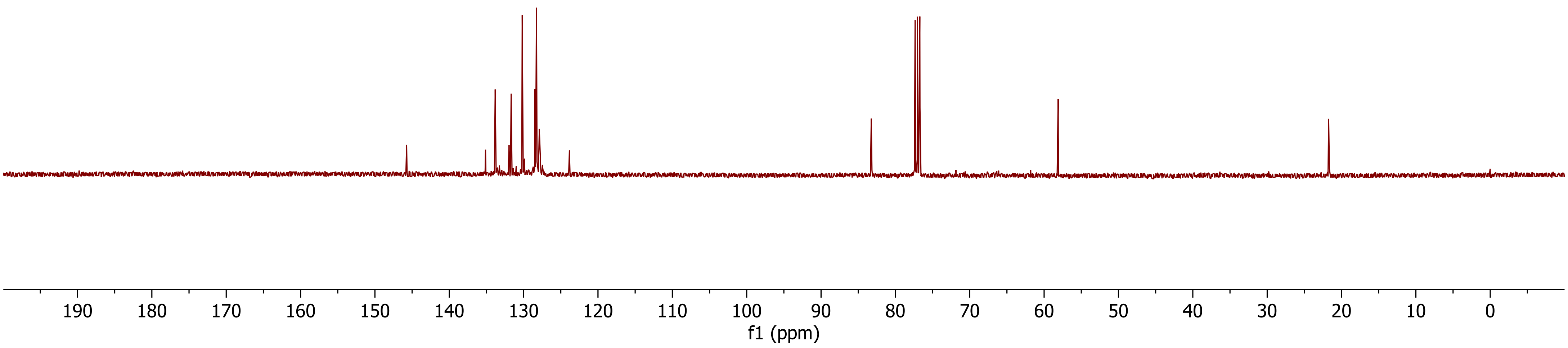

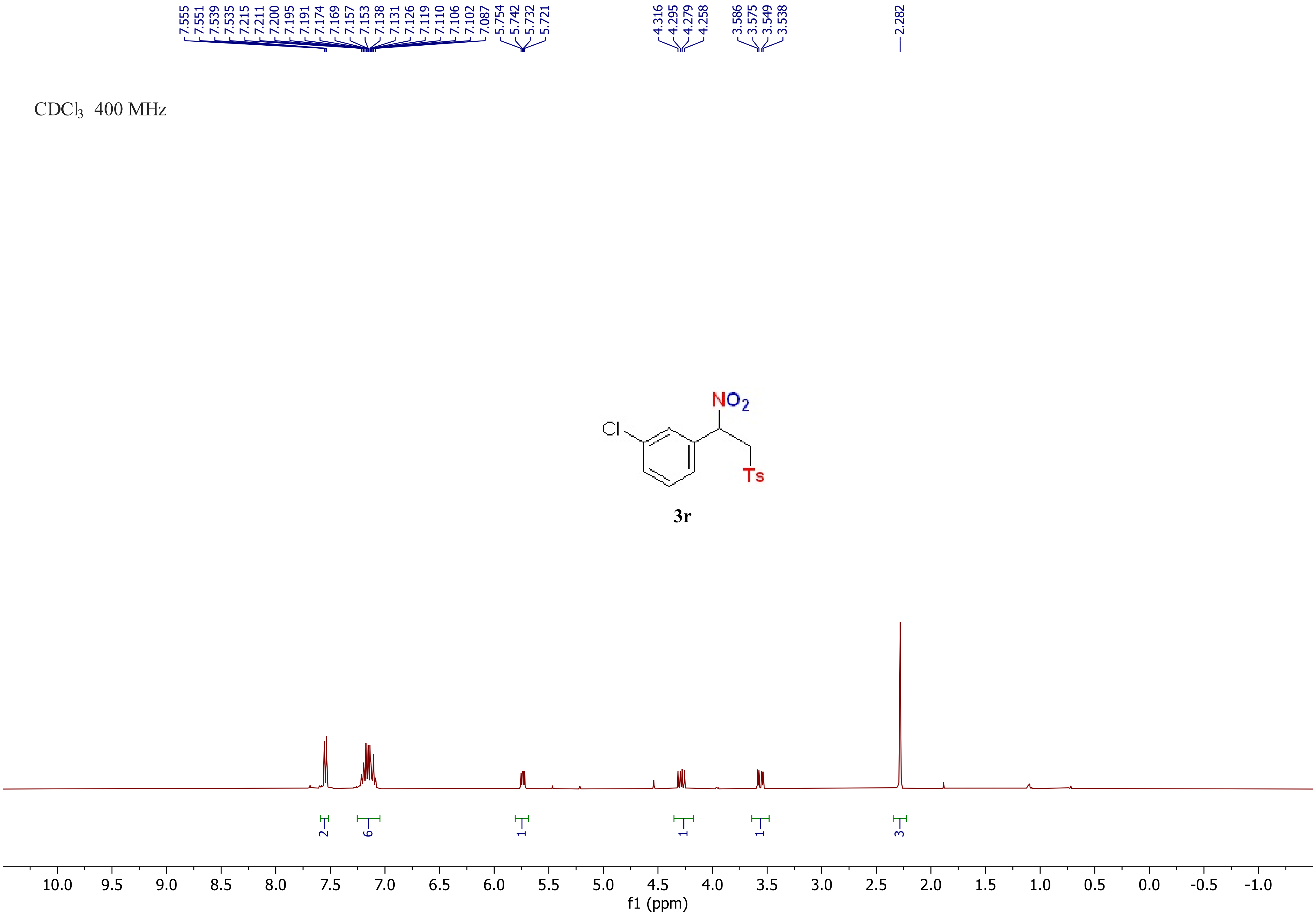
7.555
7.551
7.539
7.535
7.215
7.211
7.200
7.195
7.191
7.174
7.169
7.157
7.153
7.138
7.131
7.126
7.119
7.110
7.106
7.102
7.087
5.754
5.742
5.732
5.721
4.316
4.295
4.279
4.258
3.586
3.575
3.549
3.538
2.282
CDCl3 400 MHz
NO2
Cl
Ts
2
6
1
1
1
3
10.0 9.5 9.0 8.5 8.0 7.5 7.0 6.5 6.0 5.5 5.0 4.5 4.0 3.5 3.0 2.5 2.0 1.5 1.0 0.5 0.0 -0.5 -1.0
f1 (ppm)

3r

CDCl$_3$ 13C{1H} 100 MHz

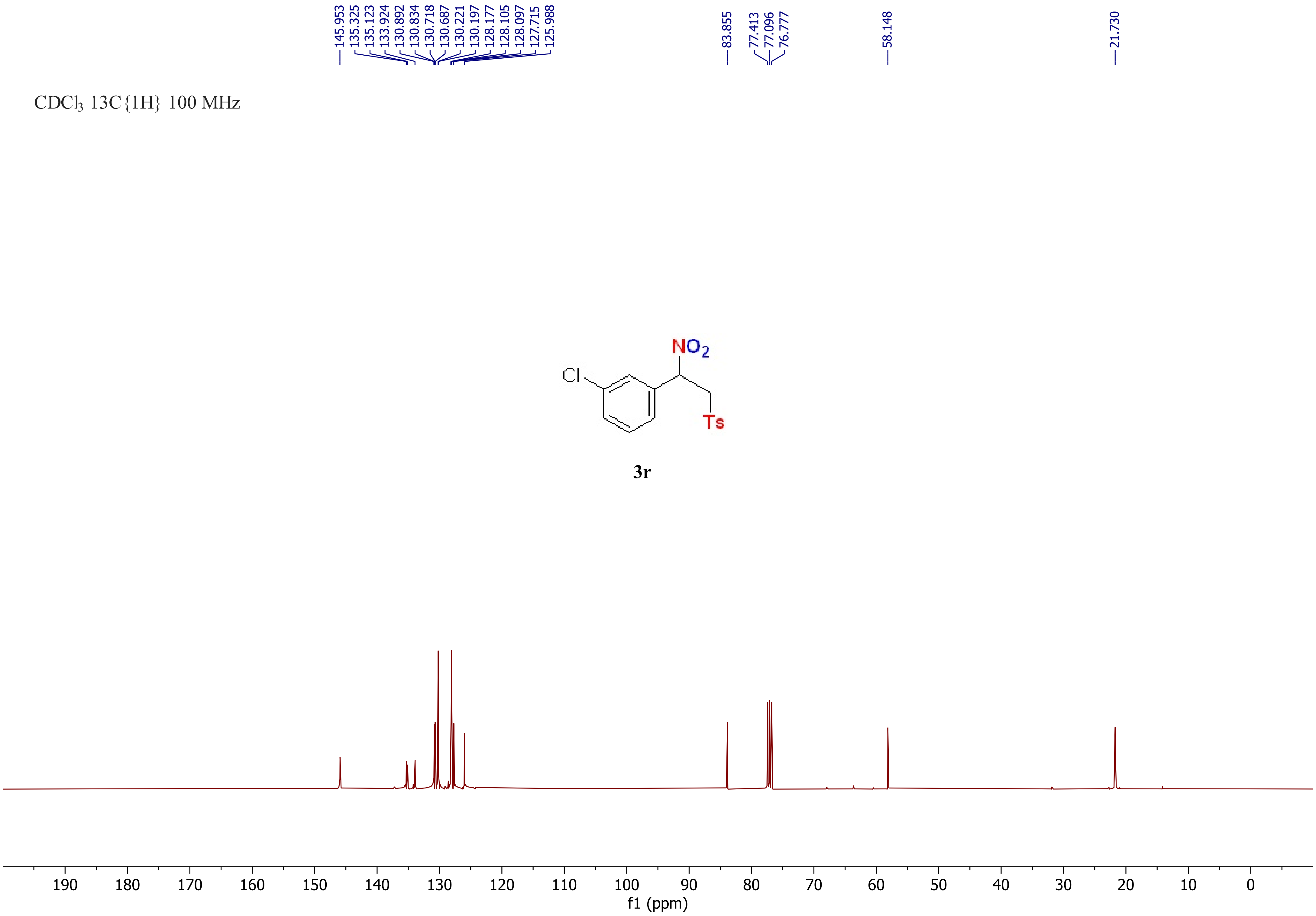

3r

$CDCl_3$ 400 MHz

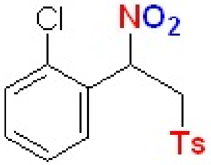

3s

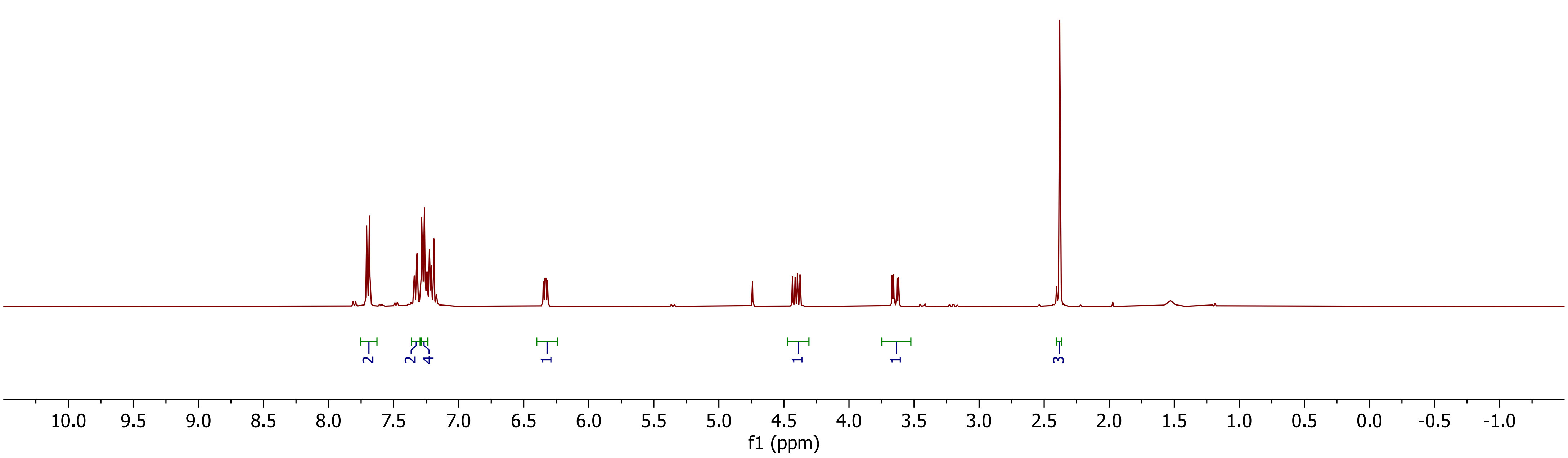

CDCl$_3$ 13C{1H} 100 MHz

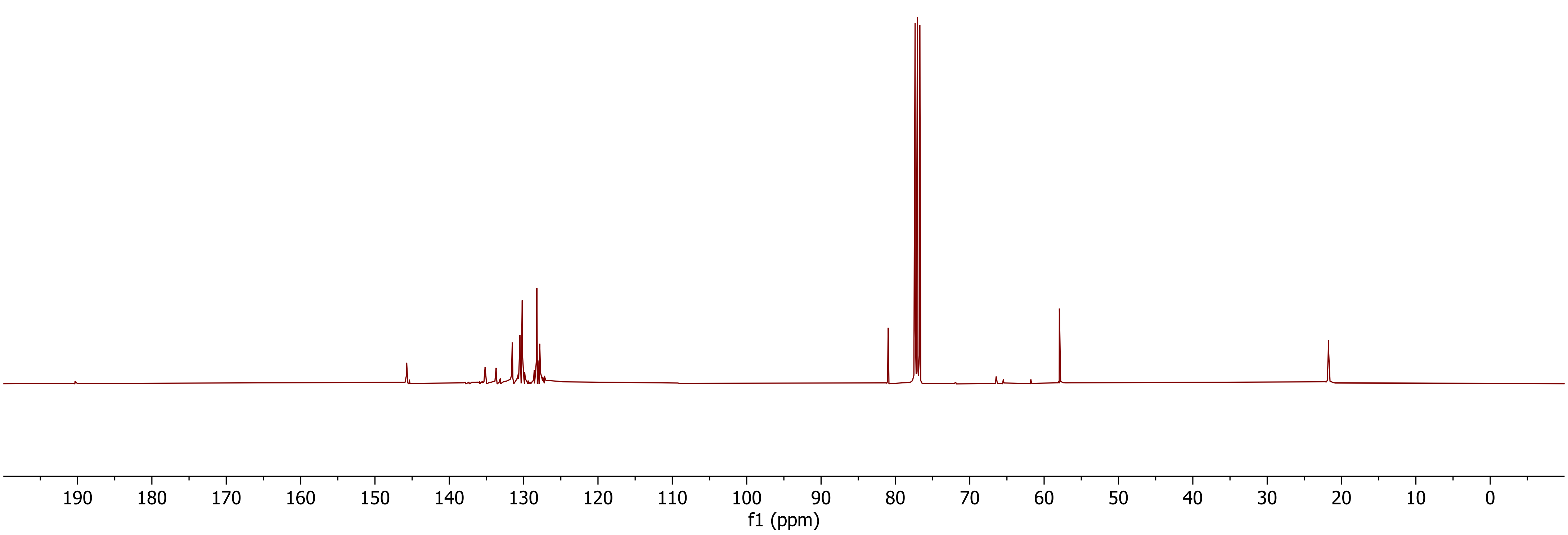

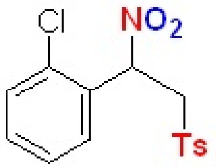

3s

CDCl$_3$ 400 MHz

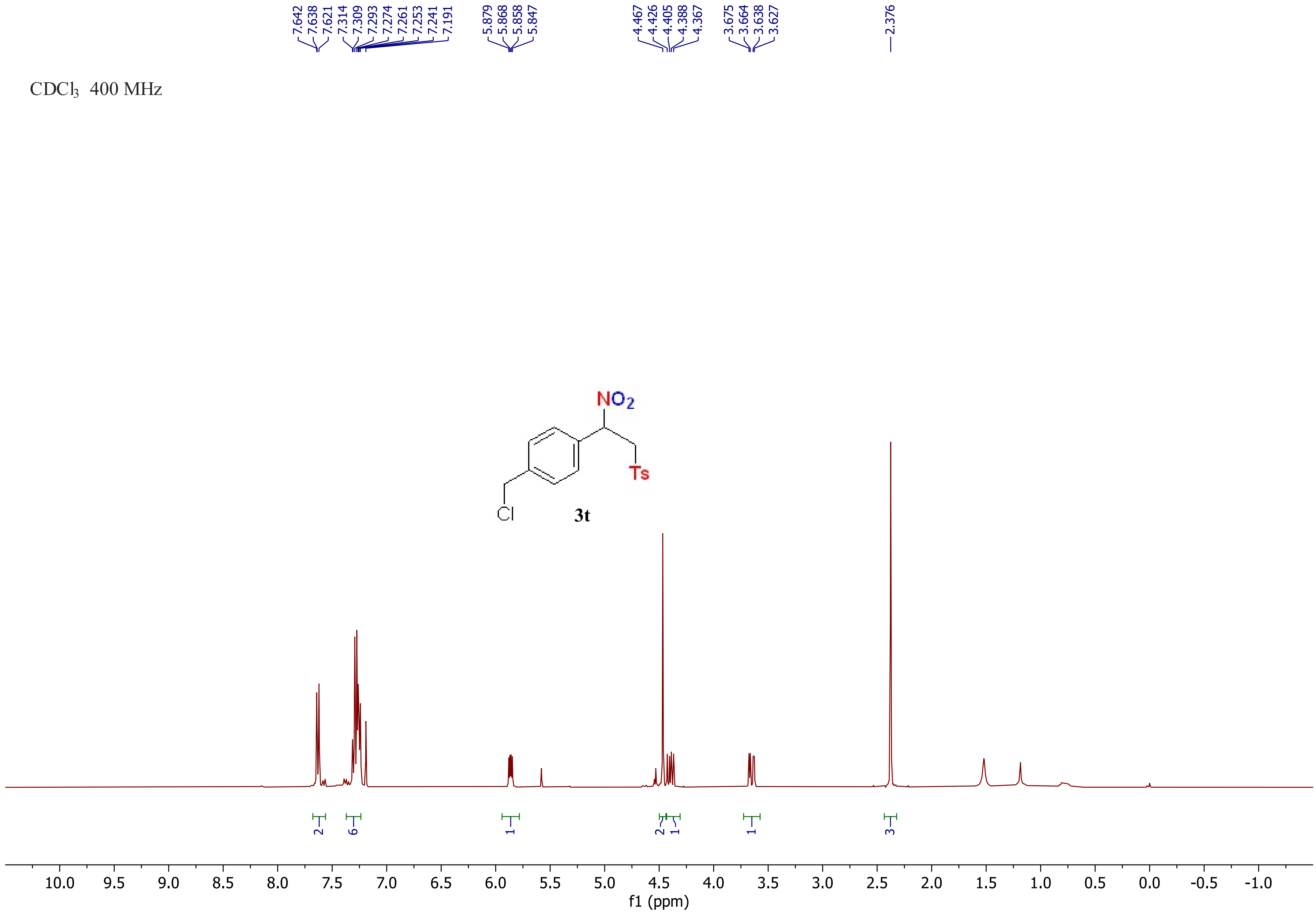

3t

CDCl$_3$ 13C{1H} 100 MHz

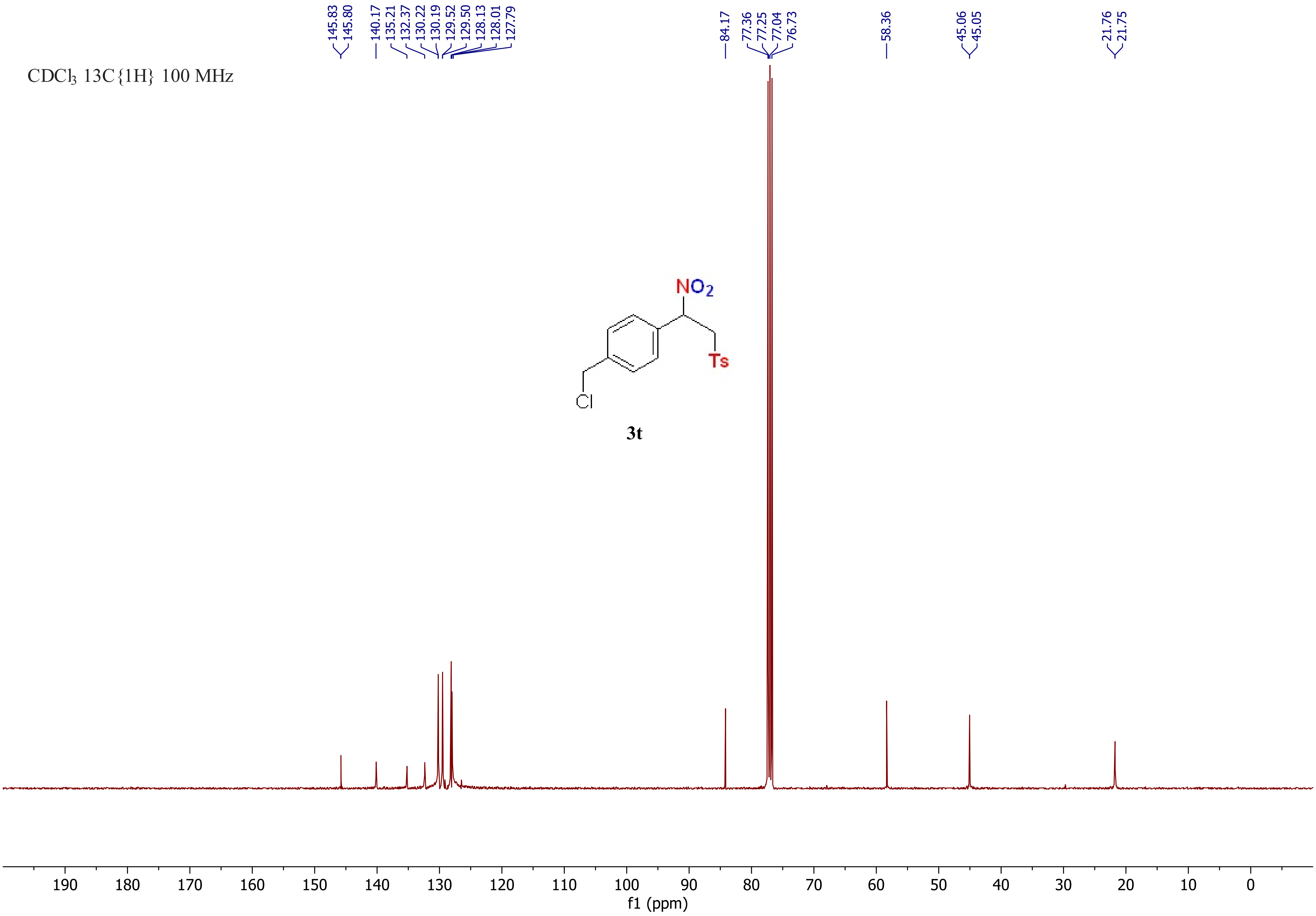

3t

CDCl$_3$ 400 MHz

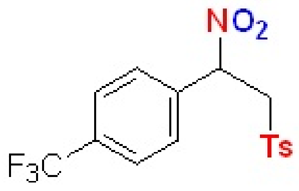

3u

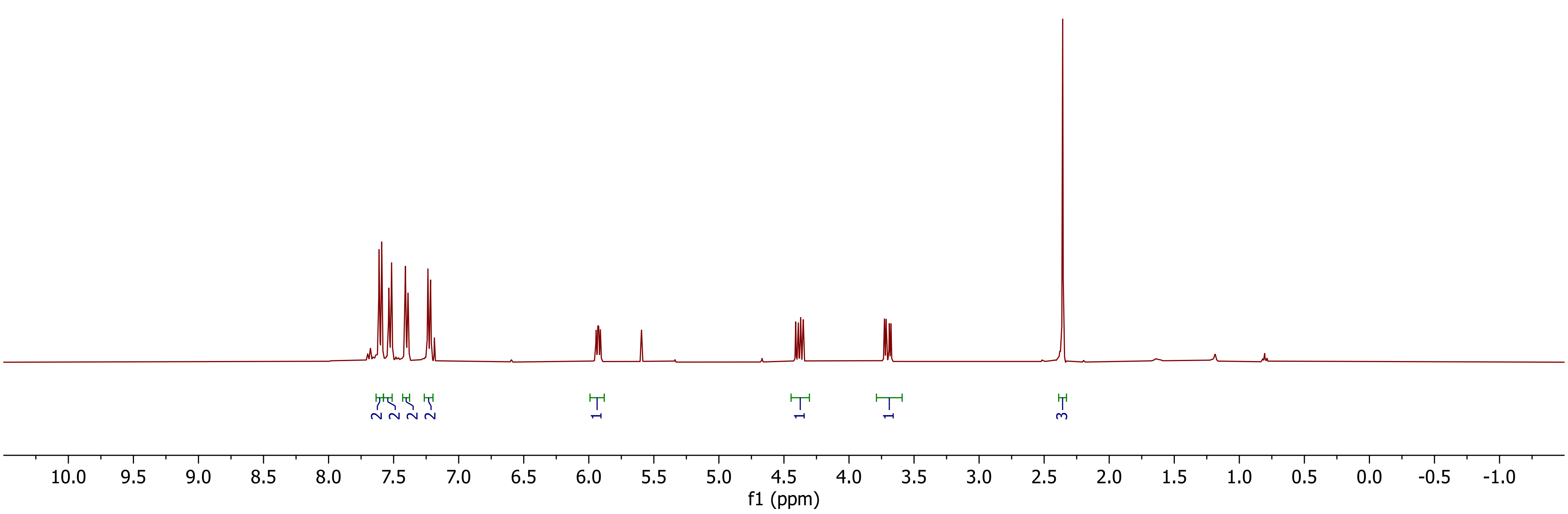

CDCl3 13C{1H} 100 MHz

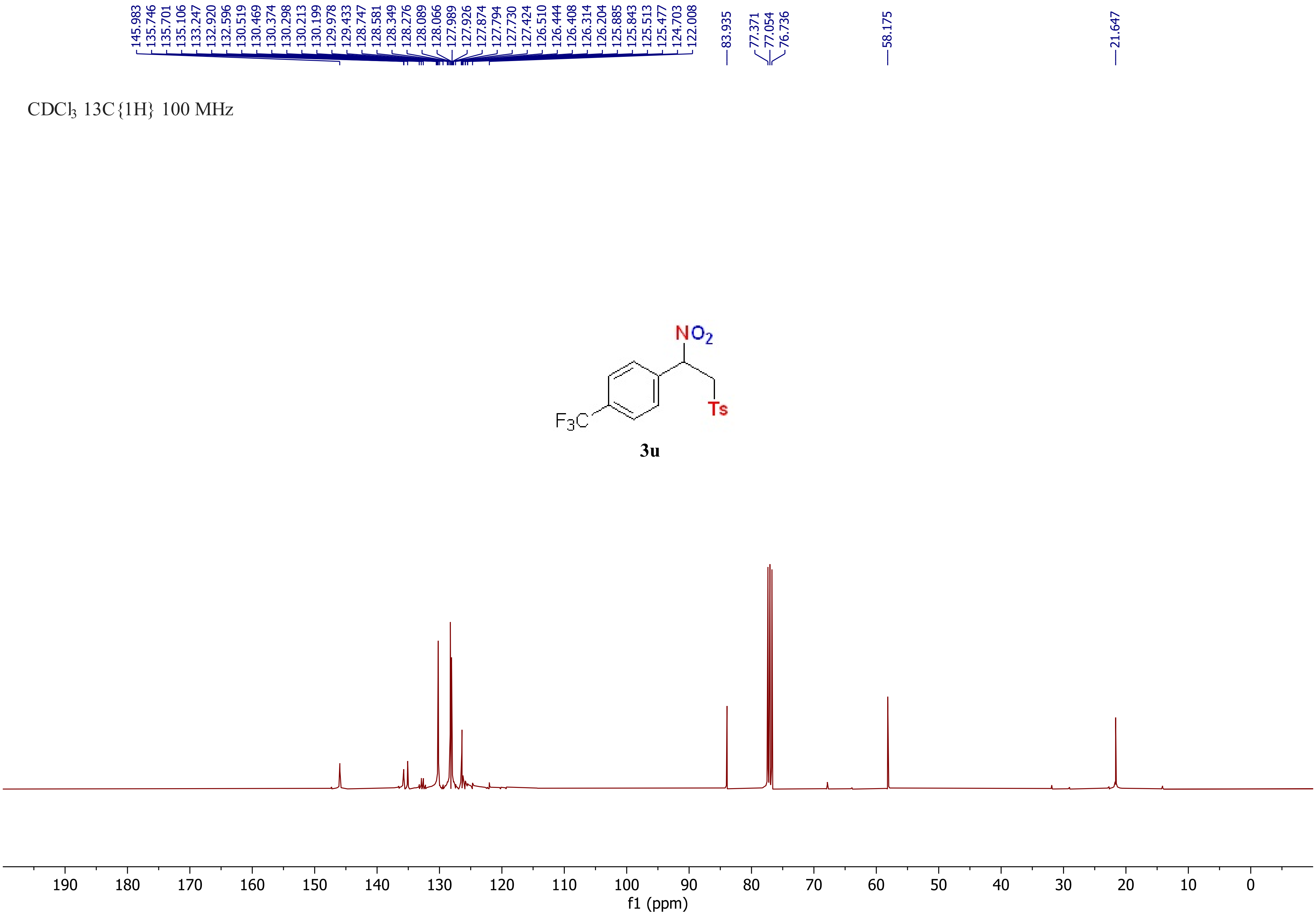

3u

CDCl$_3$ 400 MHz

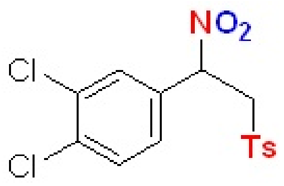

3v

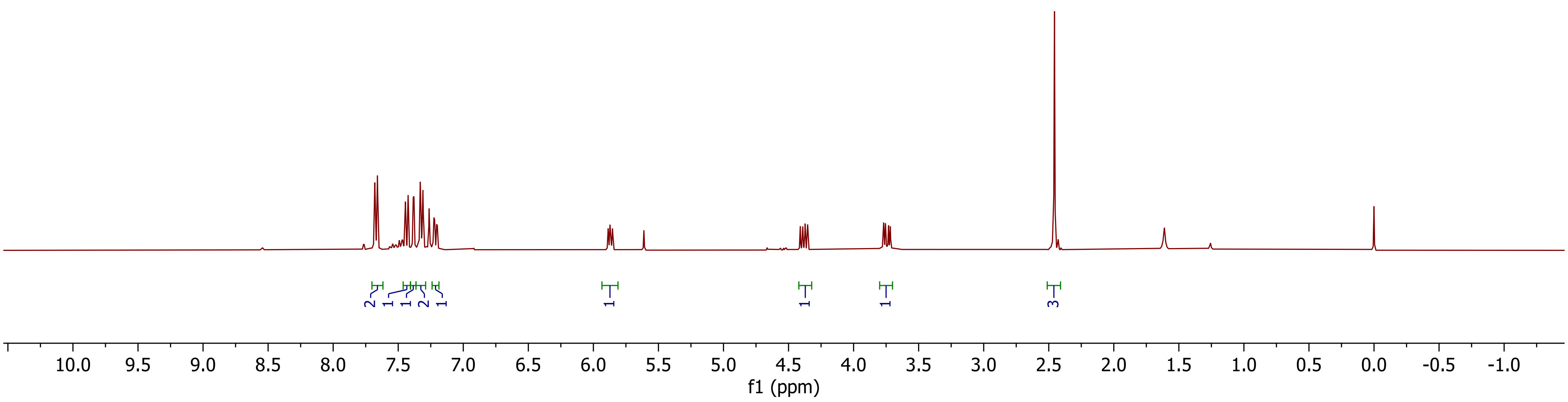

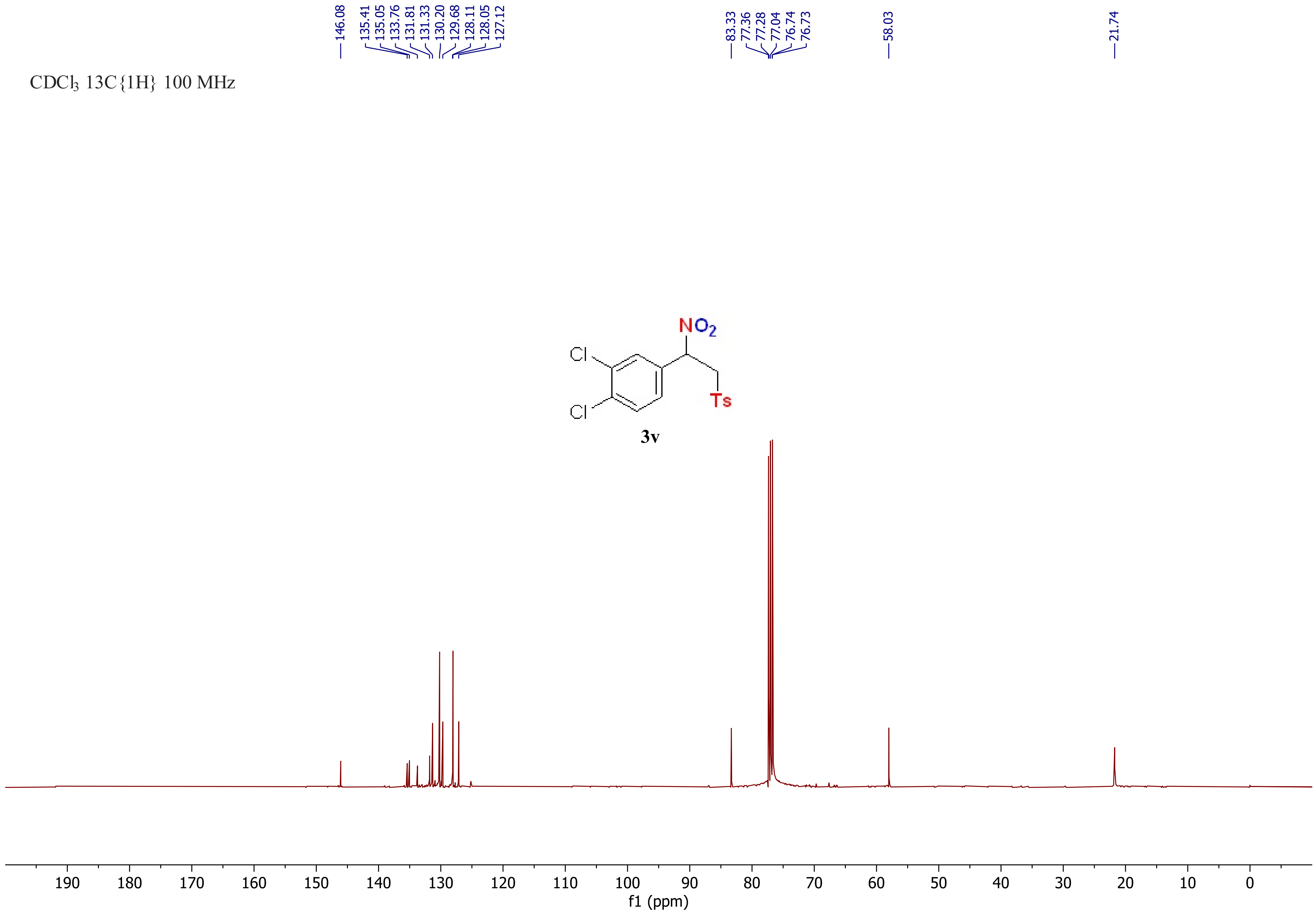

146.08
135.41
135.05
133.76
131.81
131.33
130.20
129.68
128.11
128.05
127.12
83.33
77.36
77.28
77.04
76.74
76.73
58.03
21.74
CDCl3 13C{1H} 100 MHz
NO2
Cl
Cl
Ts
3v
190
180
170
160
150
140
130
120
110
100
90
80
70
60
50
40
30
20
10
0
f1 (ppm)

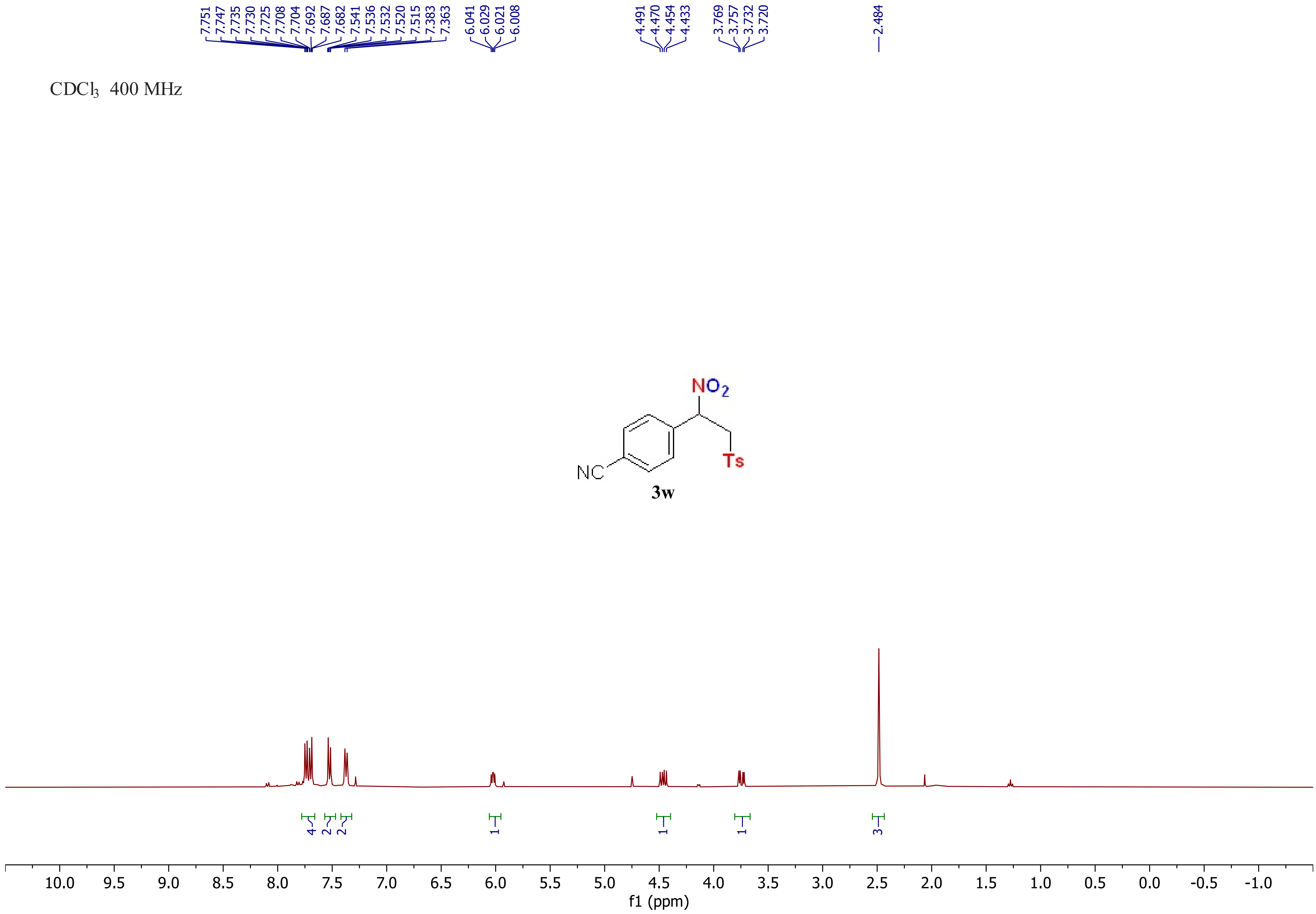
7.751
7.747
7.735
7.730
7.725
7.708
7.704
7.692
7.687
7.682
7.541
7.536
7.532
7.520
7.515
7.383
7.363
6.041
6.029
6.021
6.008
4.491
4.470
4.454
4.433
3.769
3.757
3.732
3.720
2.484
$CDCl_3$ 400 MHz
NO_2
Ts
NC
3w
4
2
2
1
1
1
3
10.0 9.5 9.0 8.5 8.0 7.5 7.0 6.5 6.0 5.5 5.0 4.5 4.0 3.5 3.0 2.5 2.0 1.5 1.0 0.5 0.0 -0.5 -1.0
f1 (ppm)

CDCl$_3$ 13C{1H} 100 MHz

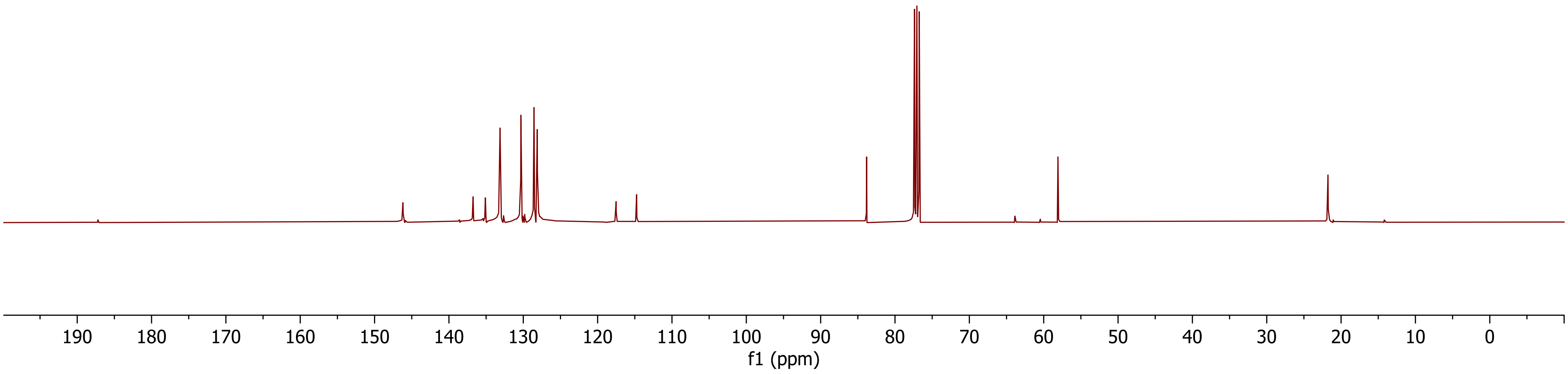

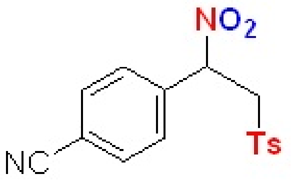

3w

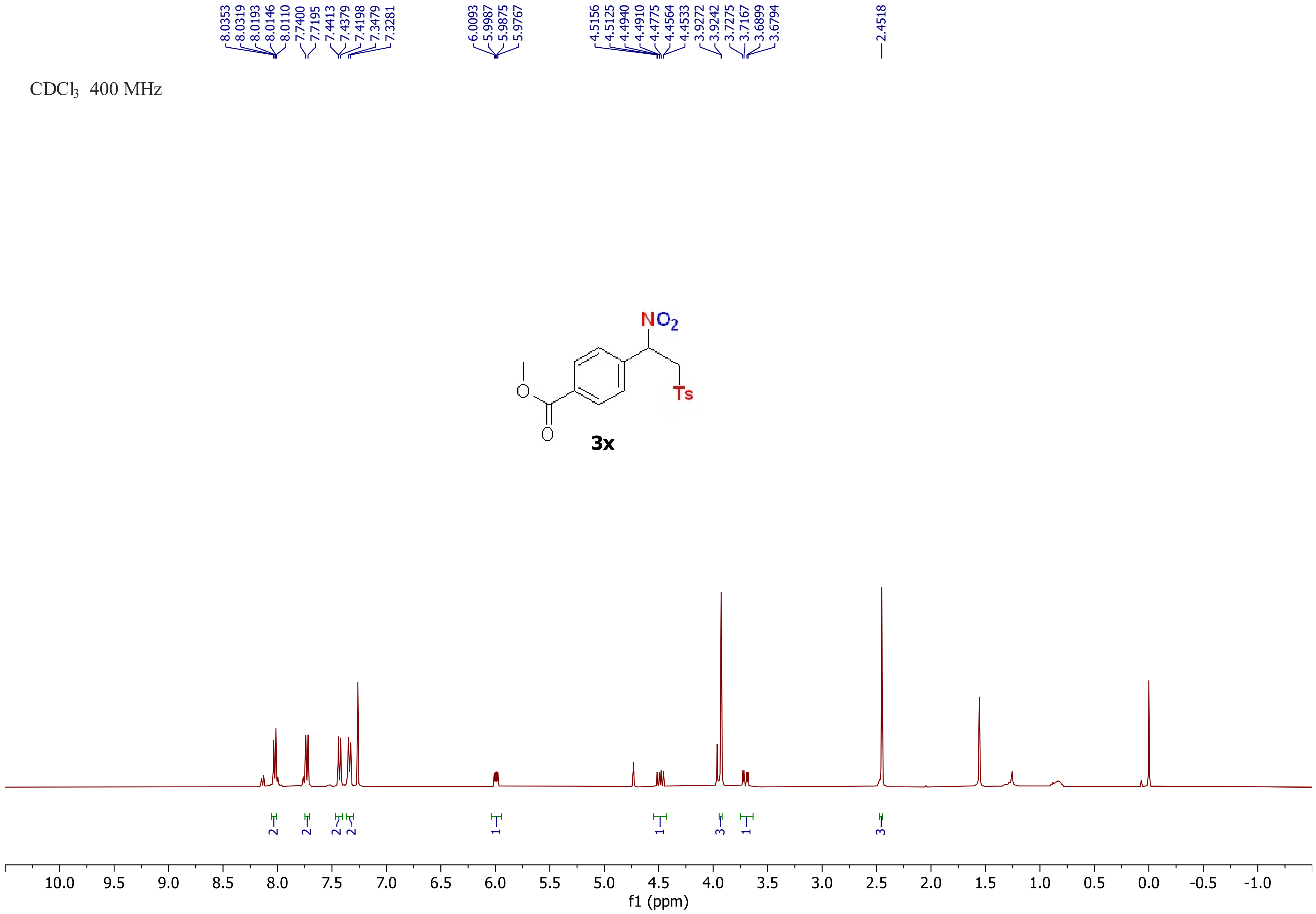
CDCl$_3$ 400 MHz
8.0353
8.0319
8.0193
8.0146
8.0110
7.7400
7.7195
7.4413
7.4379
7.4198
7.3479
7.3281
6.0093
5.9987
5.9875
5.9767
4.5156
4.5125
4.4940
4.4910
4.4775
4.4564
4.4533
3.9272
3.9242
3.7275
3.7167
3.6899
3.6794
2.4518
NO$_2$
Ts
3x
2
2
2
2
1
1
3
1
3
10.0
9.5
9.0
8.5
8.0
7.5
7.0
6.5
6.0
5.5
5.0
4.5
4.0
3.5
3.0
2.5
2.0
1.5
1.0
0.5
0.0
-0.5
-1.0
f1 (ppm)

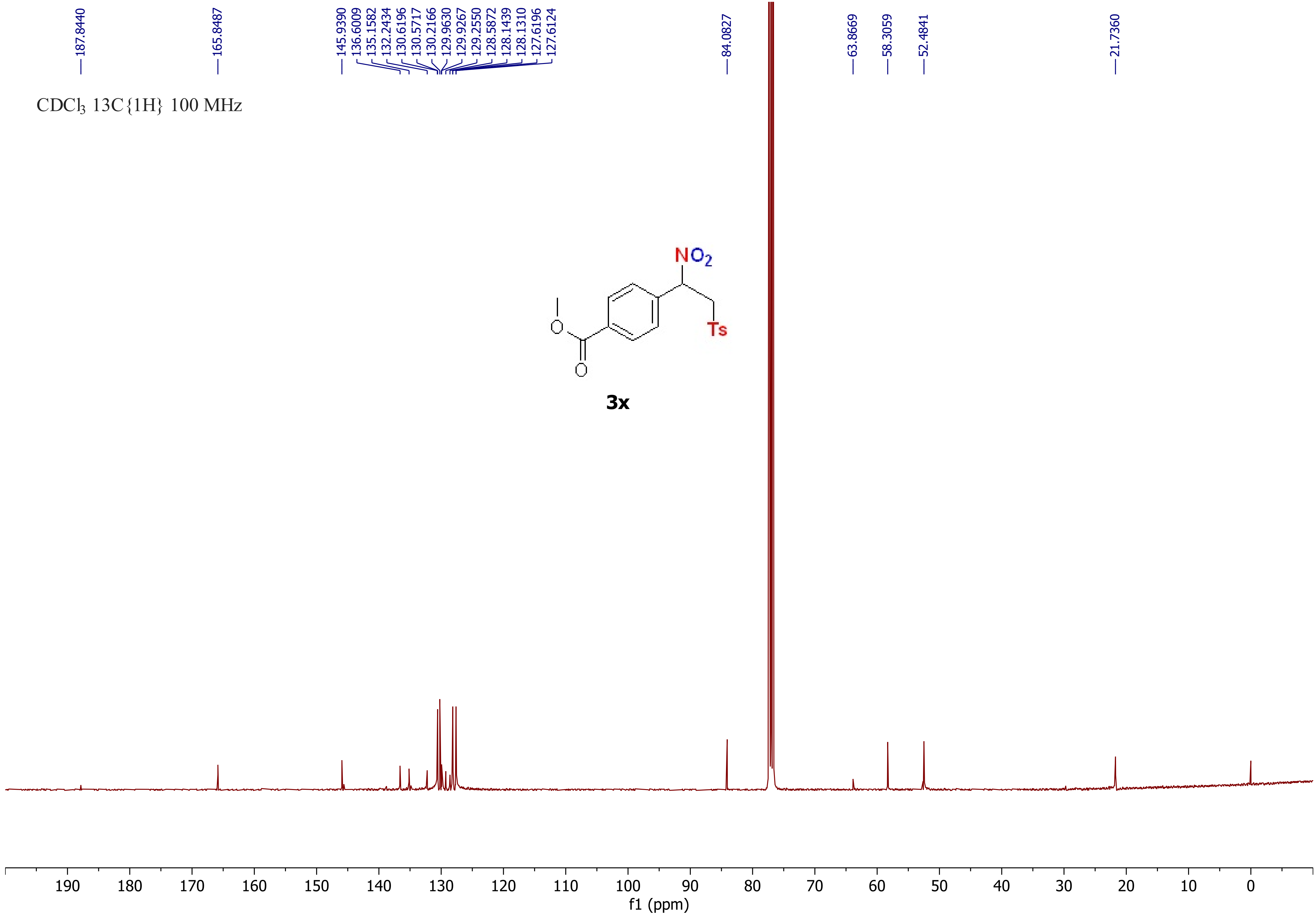
187.8440
165.8487
145.9390
136.6009
135.1582
132.2434
130.6196
130.5717
130.2166
129.9630
129.9267
129.2550
128.5872
128.1439
128.1310
127.6196
127.6124
84.0827
63.8669
58.3059
52.4841
21.7360
CDCl3 13C{1H} 100 MHz
NO2
Ts
O
O
3x
190 180 170 160 150 140 130 120 110 100 90 80 70 60 50 40 30 20 10 0
f1 (ppm)

CDCl$_3$ 400 MHz

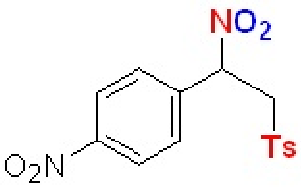

3y

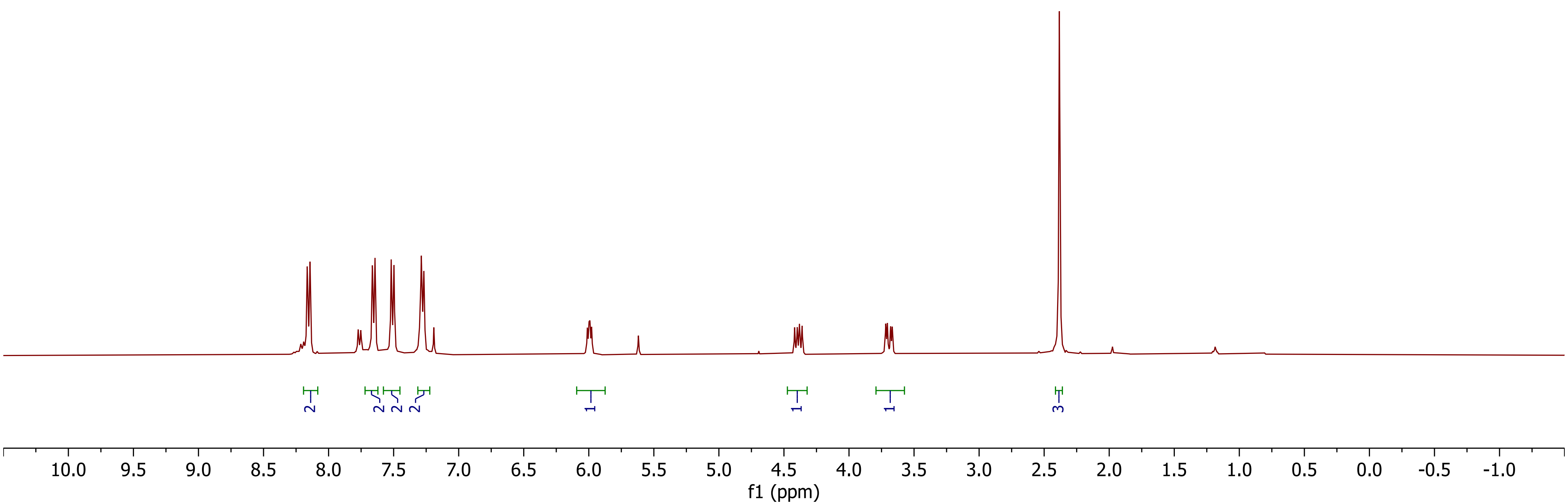

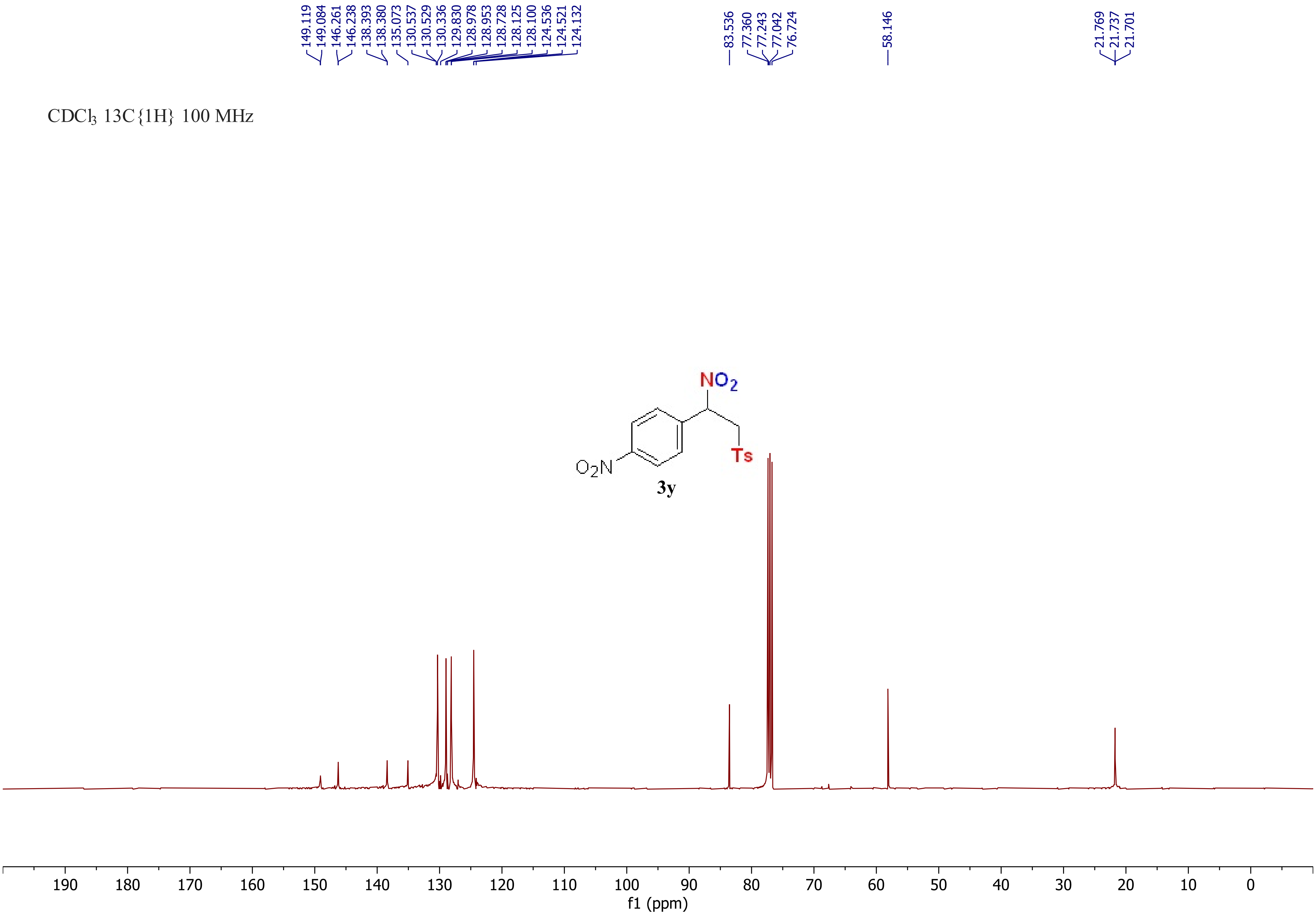
149.119
149.084
146.261
146.238
138.393
138.380
135.073
130.537
130.529
130.336
129.830
128.978
128.953
128.728
128.125
128.100
124.536
124.521
124.132
83.536
77.360
77.243
77.042
76.724
58.146
21.769
21.737
21.701
CDCl3 13C{1H} 100 MHz
NO2
Ts
O2N
3y
190 180 170 160 150 140 130 120 110 100 90 80 70 60 50 40 30 20 10 0
f1 (ppm)

CDCl$_3$ 400 MHz

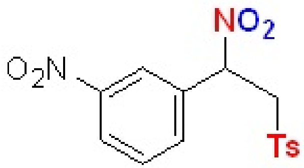

3z

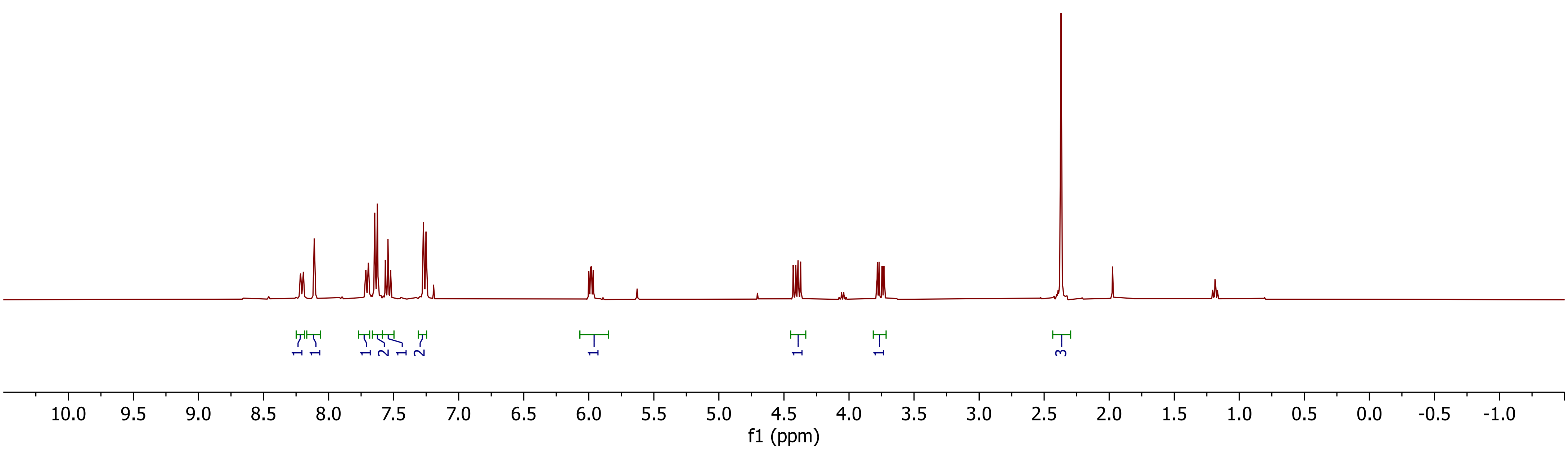

CDCl$_3$ 13C{1H} 100 MHz

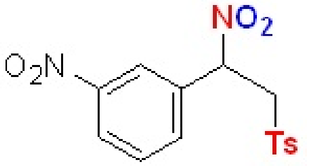

3z

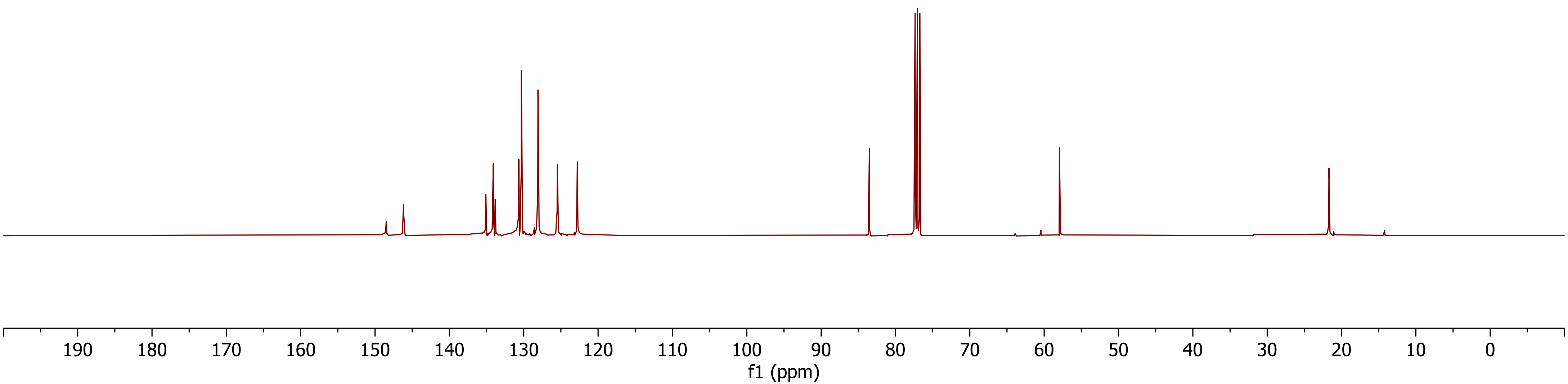

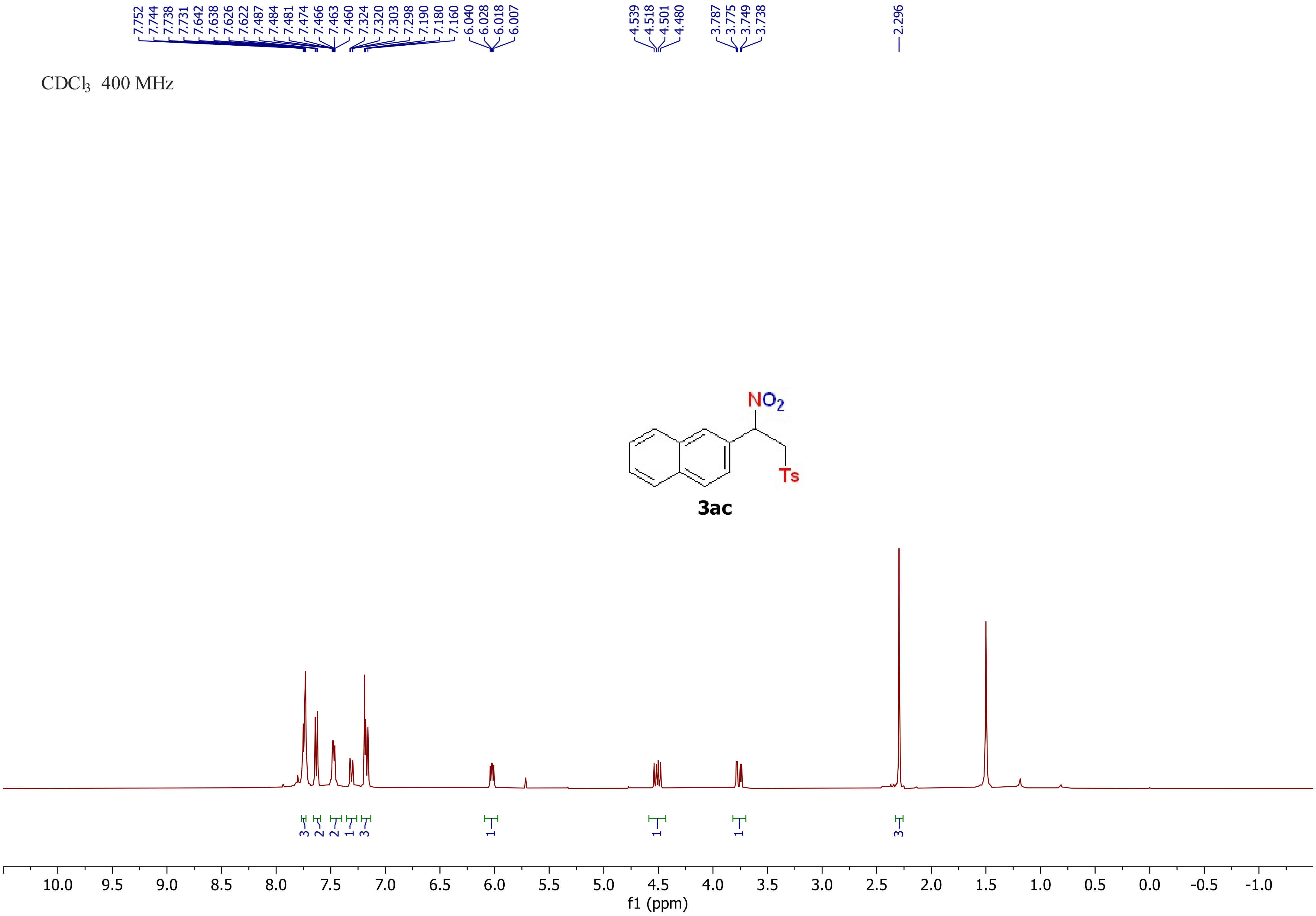
7.752 7.744 7.738 7.731 7.642 7.638 7.626 7.622 7.487 7.484 7.481 7.474 7.466 7.463 7.460 7.324 7.320 7.303 7.298 7.190 7.180 7.160 6.040 6.028 6.018 6.007
4.539 4.518 4.501 4.480
3.787 3.775 3.749 3.738
2.296
CDCl3 400 MHz
NO2
Ts
3ac
3 2 2 1 3 1 1 1 3
10.0 9.5 9.0 8.5 8.0 7.5 7.0 6.5 6.0 5.5 5.0 4.5 4.0 3.5 3.0 2.5 2.0 1.5 1.0 0.5 0.0 -0.5 -1.0
f1 (ppm)

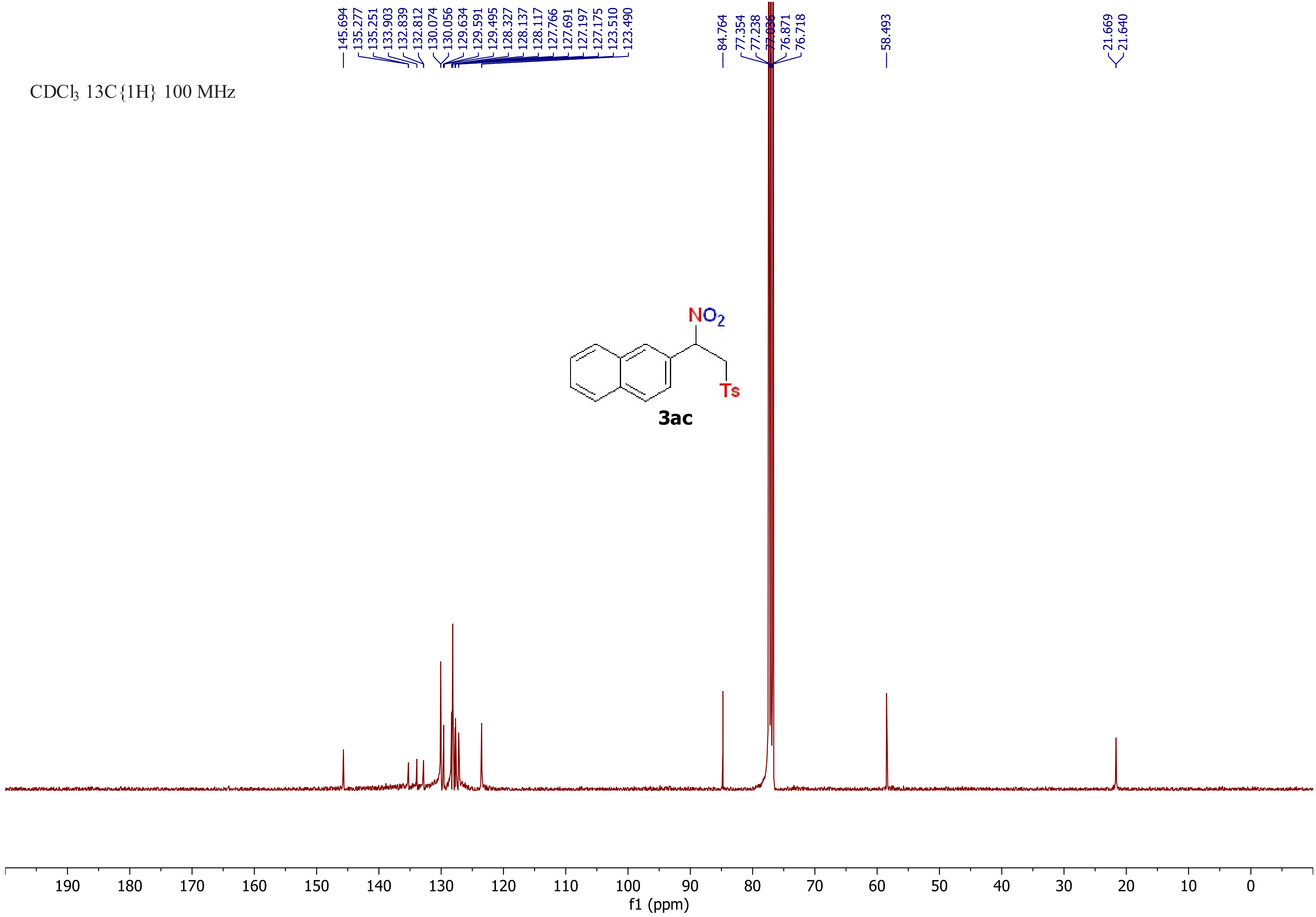

CDCl3 13C{1H} 100 MHz
145.694
135.277
135.251
133.903
132.839
132.812
130.074
130.056
129.634
129.591
129.495
128.327
128.137
128.117
127.766
127.691
127.197
127.175
123.510
123.490
84.764
77.354
77.238
77.036
76.871
76.718
58.493
21.669
21.640
NO2
Ts
3ac
190
180
170
160
150
140
130
120
110
100
90
80
70
60
50
40
30
20
10
0
f1 (ppm)

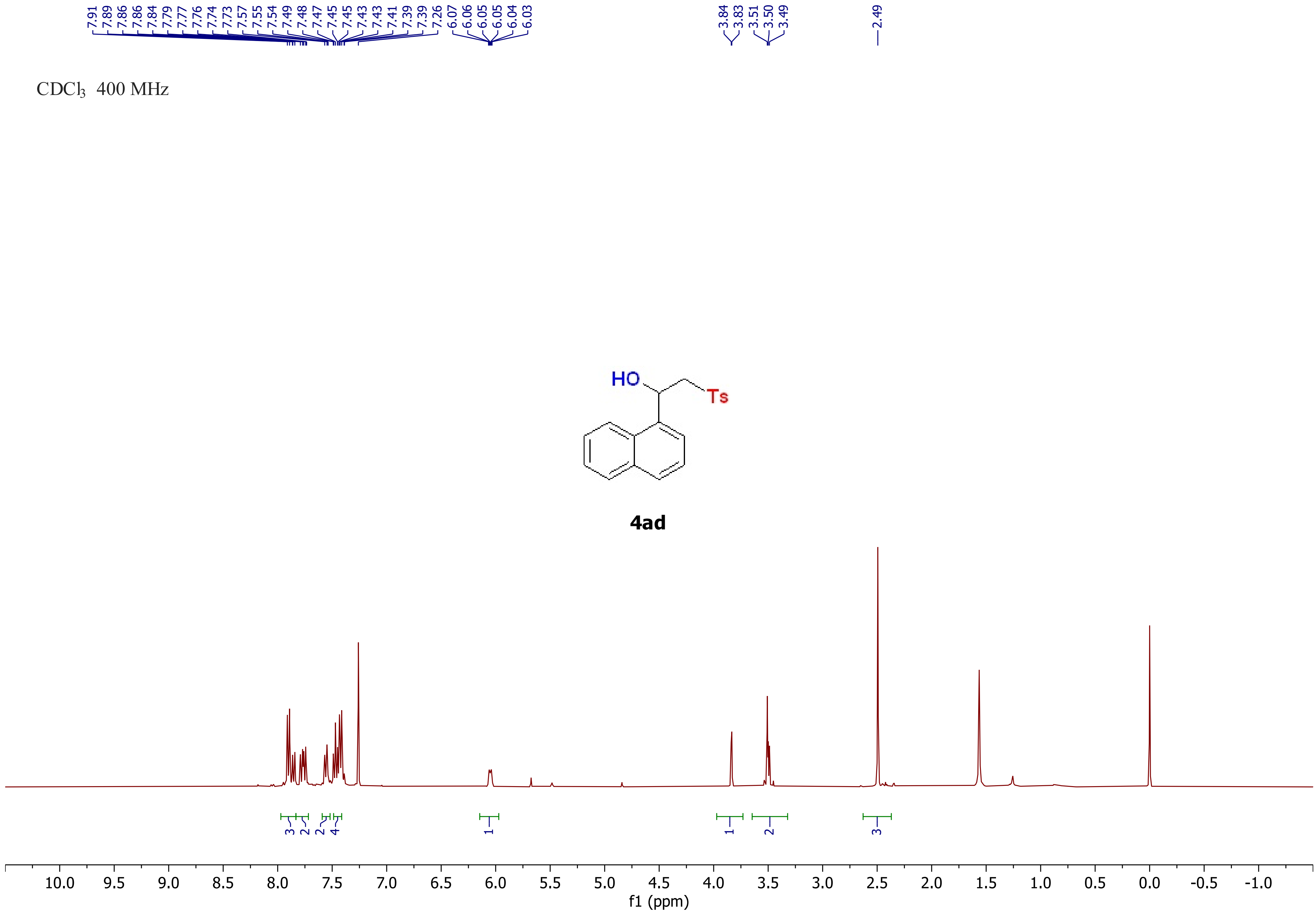
7.91
7.89
7.86
7.86
7.84
7.79
7.77
7.76
7.74
7.73
7.57
7.55
7.54
7.49
7.48
7.47
7.45
7.45
7.43
7.43
7.41
7.39
7.39
7.26
6.07
6.06
6.05
6.05
6.04
6.03
3.84
3.83
3.51
3.50
3.49
2.49
CDCl3 400 MHz
HO
Ts
4ad
3
2
2
4
1
1
2
3
10.0
9.5
9.0
8.5
8.0
7.5
7.0
6.5
6.0
5.5
5.0
4.5
4.0
3.5
3.0
2.5
2.0
1.5
1.0
0.5
0.0
-0.5
-1.0
f1 (ppm)

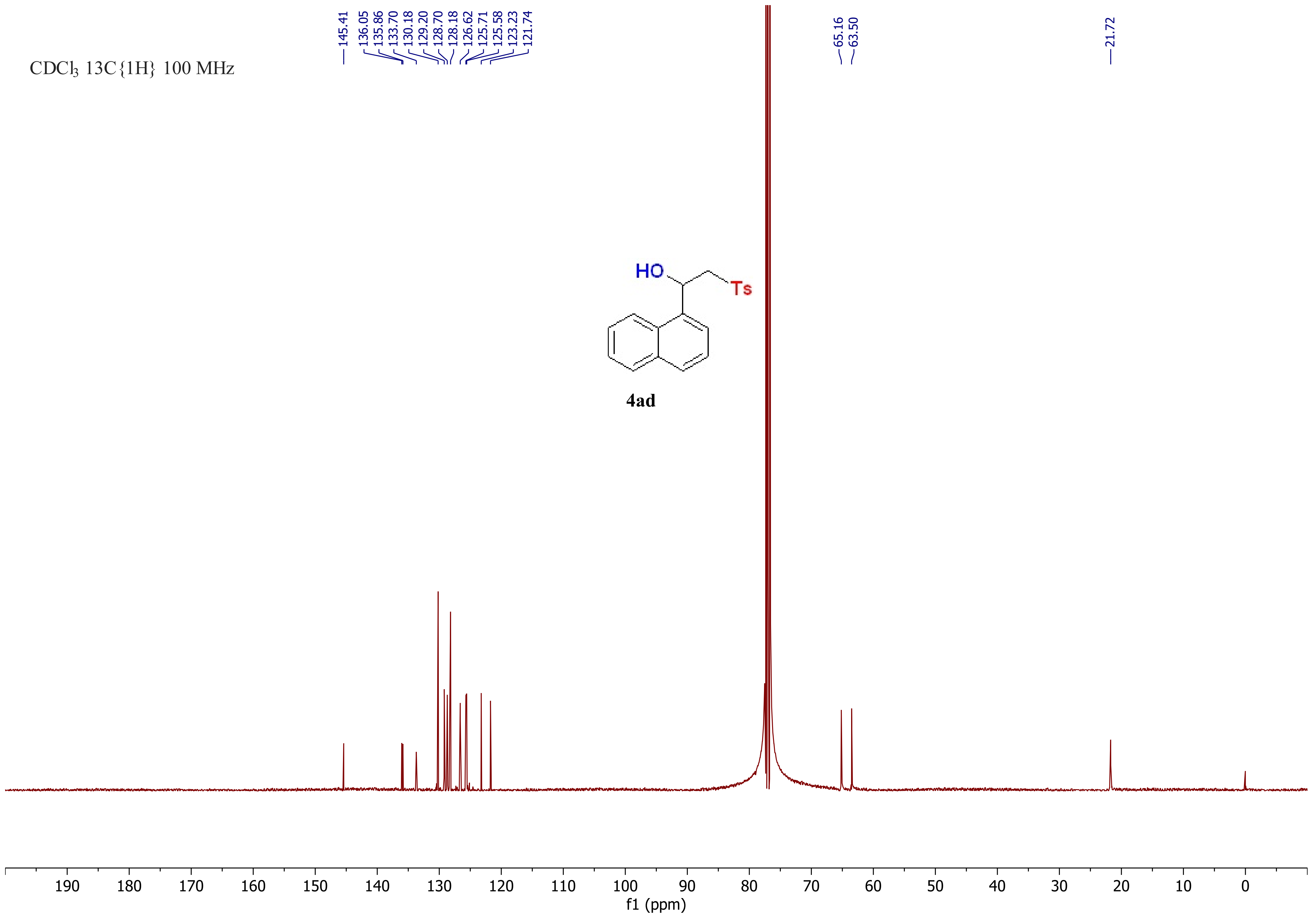
CDCl3 13C{1H} 100 MHz
145.41
136.05
135.86
133.70
130.18
129.20
128.70
128.18
126.62
125.71
125.58
123.23
121.74
65.16
63.50
21.72
HO
Ts
4ad
190 180 170 160 150 140 130 120 110 100 90 80 70 60 50 40 30 20 10 0
f1 (ppm)

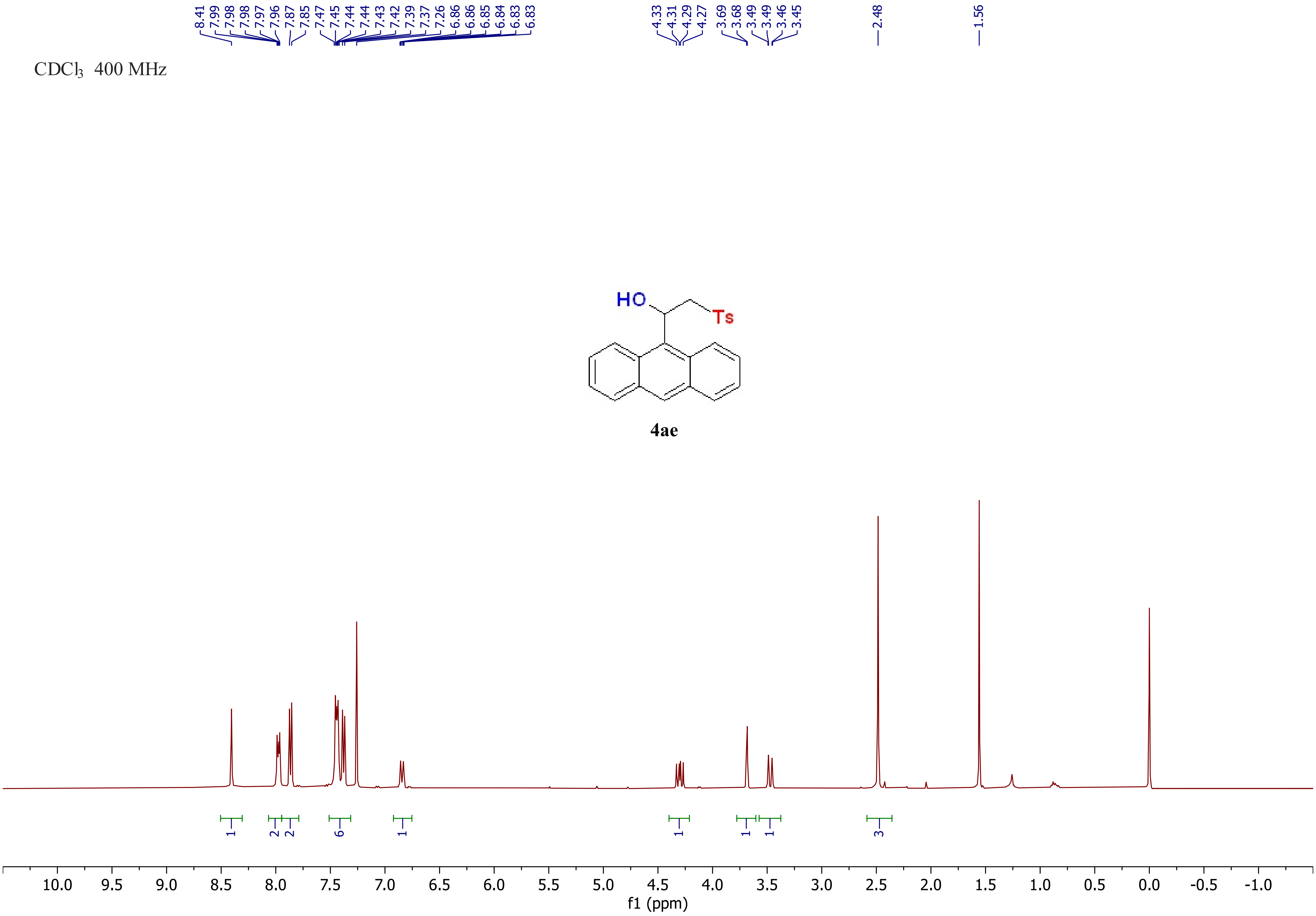
8.41
7.99
7.98
7.98
7.97
7.96
7.87
7.85
7.47
7.45
7.44
7.44
7.43
7.42
7.39
7.37
7.26
6.86
6.86
6.85
6.84
6.83
6.83
4.33
4.31
4.29
4.27
3.69
3.68
3.49
3.49
3.46
3.45
2.48
1.56
CDCl3 400 MHz
HO
Ts
4ae
1
2
2
6
1
1
1
1
3
10.0 9.5 9.0 8.5 8.0 7.5 7.0 6.5 6.0 5.5 5.0 4.5 4.0 3.5 3.0 2.5 2.0 1.5 1.0 0.5 0.0 -0.5 -1.0
f1 (ppm)

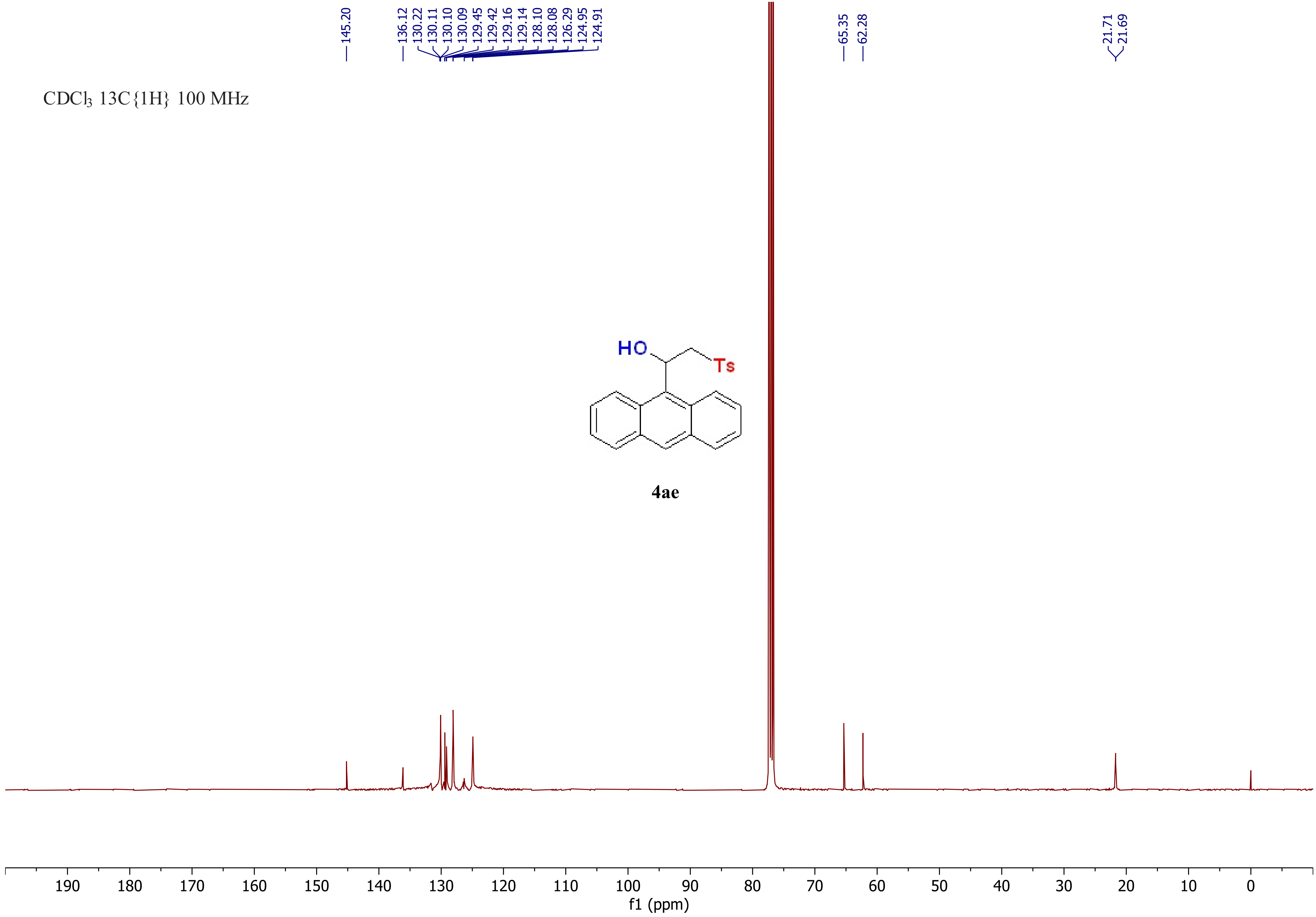
CDCl3 13C{1H} 100 MHz
145.20
136.12
130.22
130.11
130.10
130.09
129.45
129.42
129.16
129.14
128.10
128.08
126.29
124.95
124.91
65.35
62.28
21.71
21.69
HO
Ts
4ae
190
180
170
160
150
140
130
120
110
100
90
80
70
60
50
40
30
20
10
0
f1 (ppm)

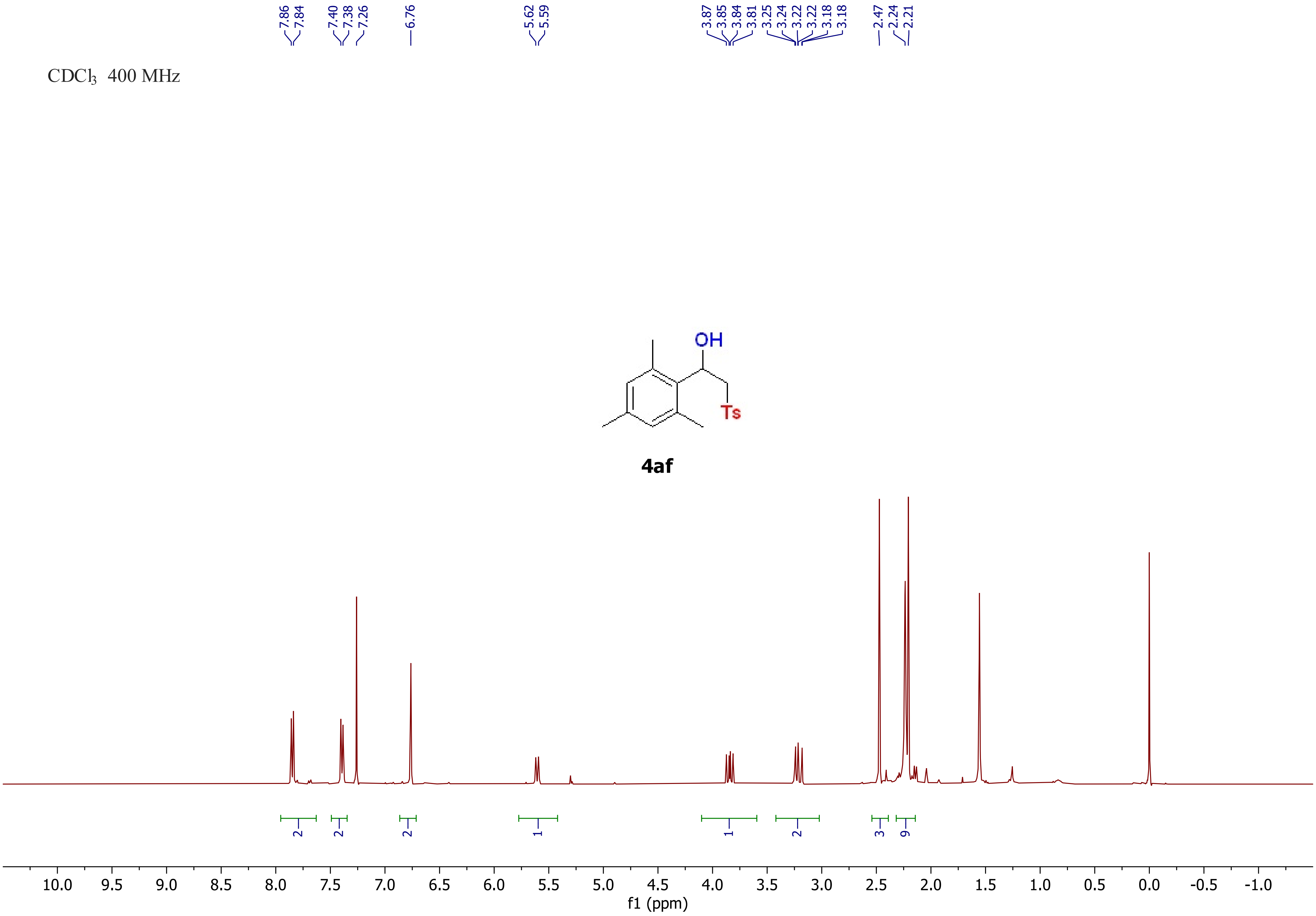

7.86
7.84
7.40
7.38
7.26
6.76
5.62
5.59
3.87
3.85
3.84
3.81
3.25
3.24
3.22
3.22
3.18
3.18
2.47
2.24
2.21
CDCl3 400 MHz
OH
Ts
4af
2
2
2
1
1
2
3
9
10.0 9.5 9.0 8.5 8.0 7.5 7.0 6.5 6.0 5.5 5.0 4.5 4.0 3.5 3.0 2.5 2.0 1.5 1.0 0.5 0.0 -0.5 -1.0
f1 (ppm)

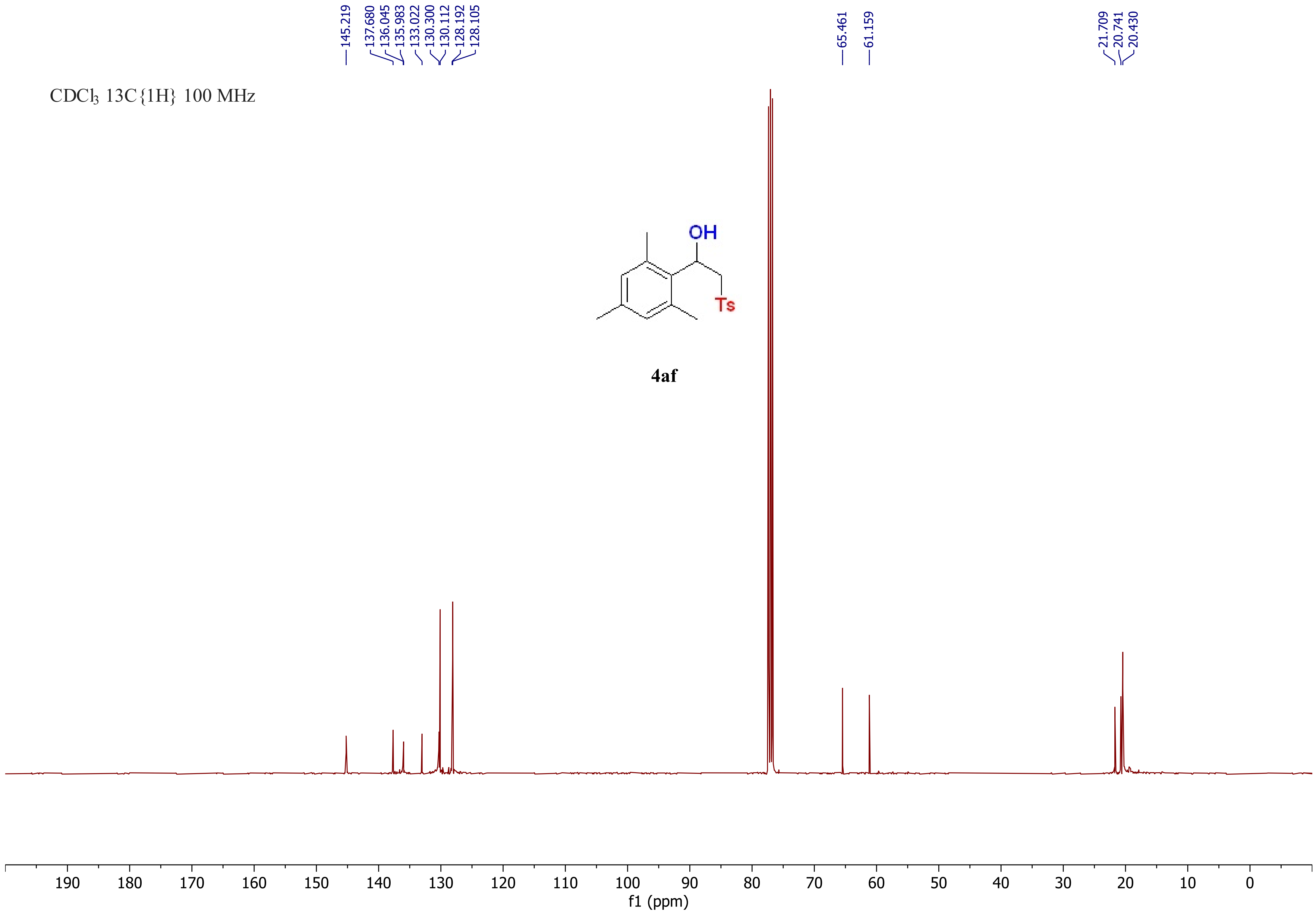
145.219
137.680
136.045
135.983
133.022
130.300
130.112
128.192
128.105
65.461
61.159
21.709
20.741
20.430
CDCl3 13C{1H} 100 MHz
OH
Ts
4af
190 180 170 160 150 140 130 120 110 100 90 80 70 60 50 40 30 20 10 0
f1 (ppm)

CDCl$_3$ 400 MHz

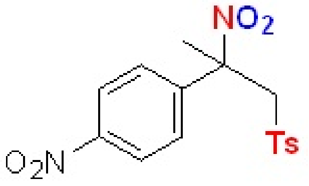

3ag

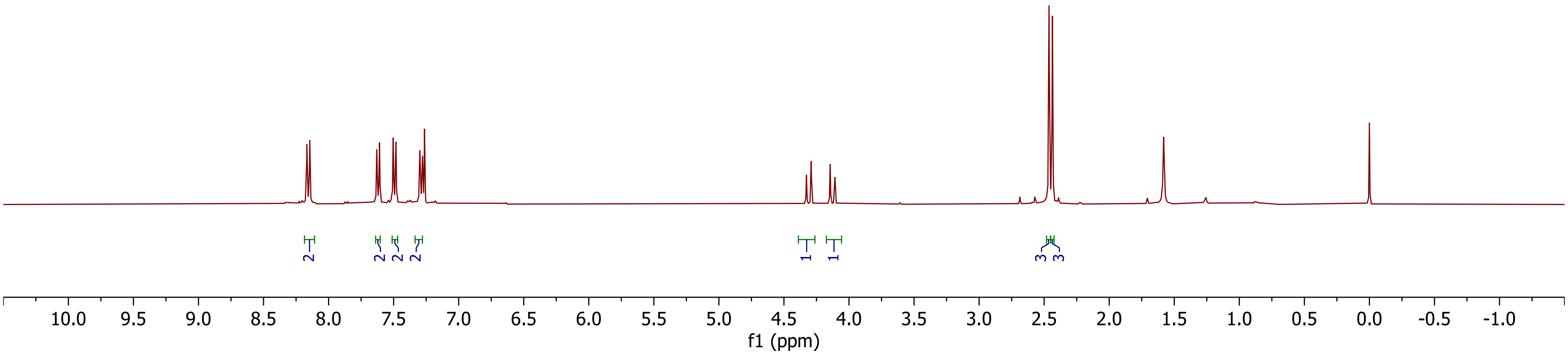

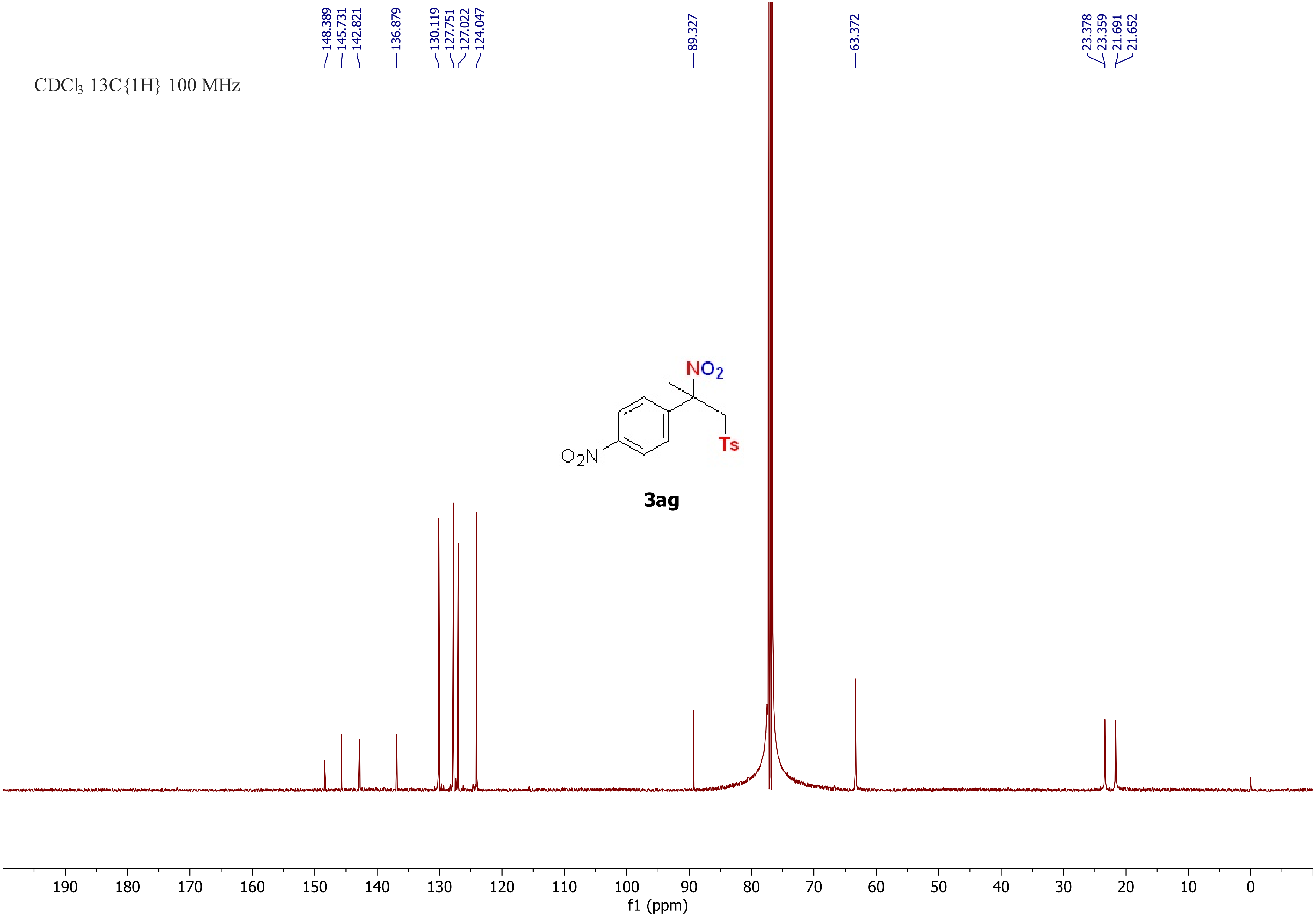
148.389
145.731
142.821
136.879
130.119
127.751
127.022
124.047
89.327
63.372
23.378
23.359
21.691
21.652
CDCl3 13C{1H} 100 MHz
NO2
O2N
Ts
3ag
190 180 170 160 150 140 130 120 110 100 90 80 70 60 50 40 30 20 10 0
f1 (ppm)

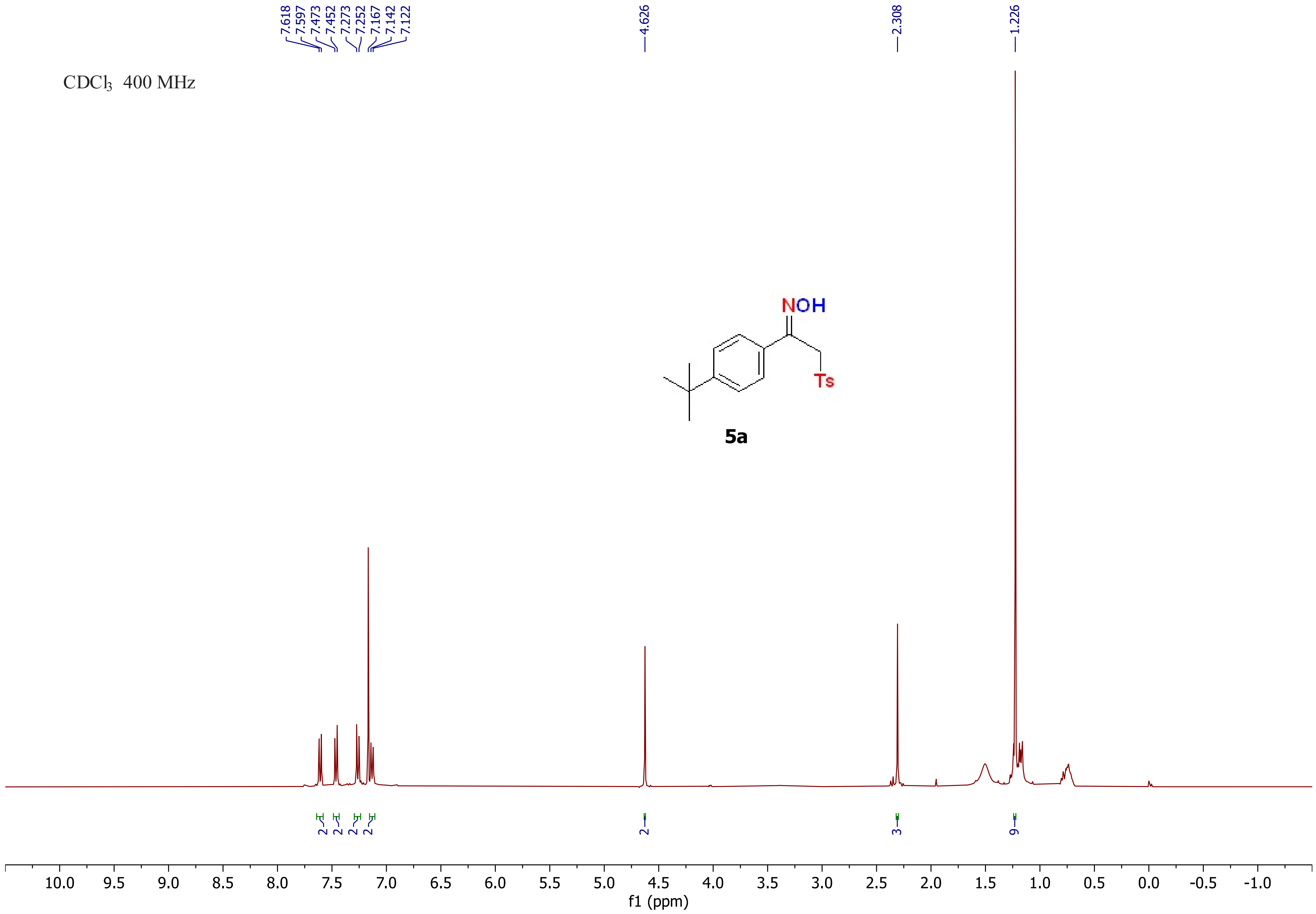

7.618
7.597
7.473
7.452
7.273
7.252
7.167
7.142
7.122
4.626
2.308
1.226
CDCl3 400 MHz
NOH
Ts
5a
2
2
2
2
2
3
9
10.0 9.5 9.0 8.5 8.0 7.5 7.0 6.5 6.0 5.5 5.0 4.5 4.0 3.5 3.0 2.5 2.0 1.5 1.0 0.5 0.0 -0.5 -1.0
f1 (ppm)

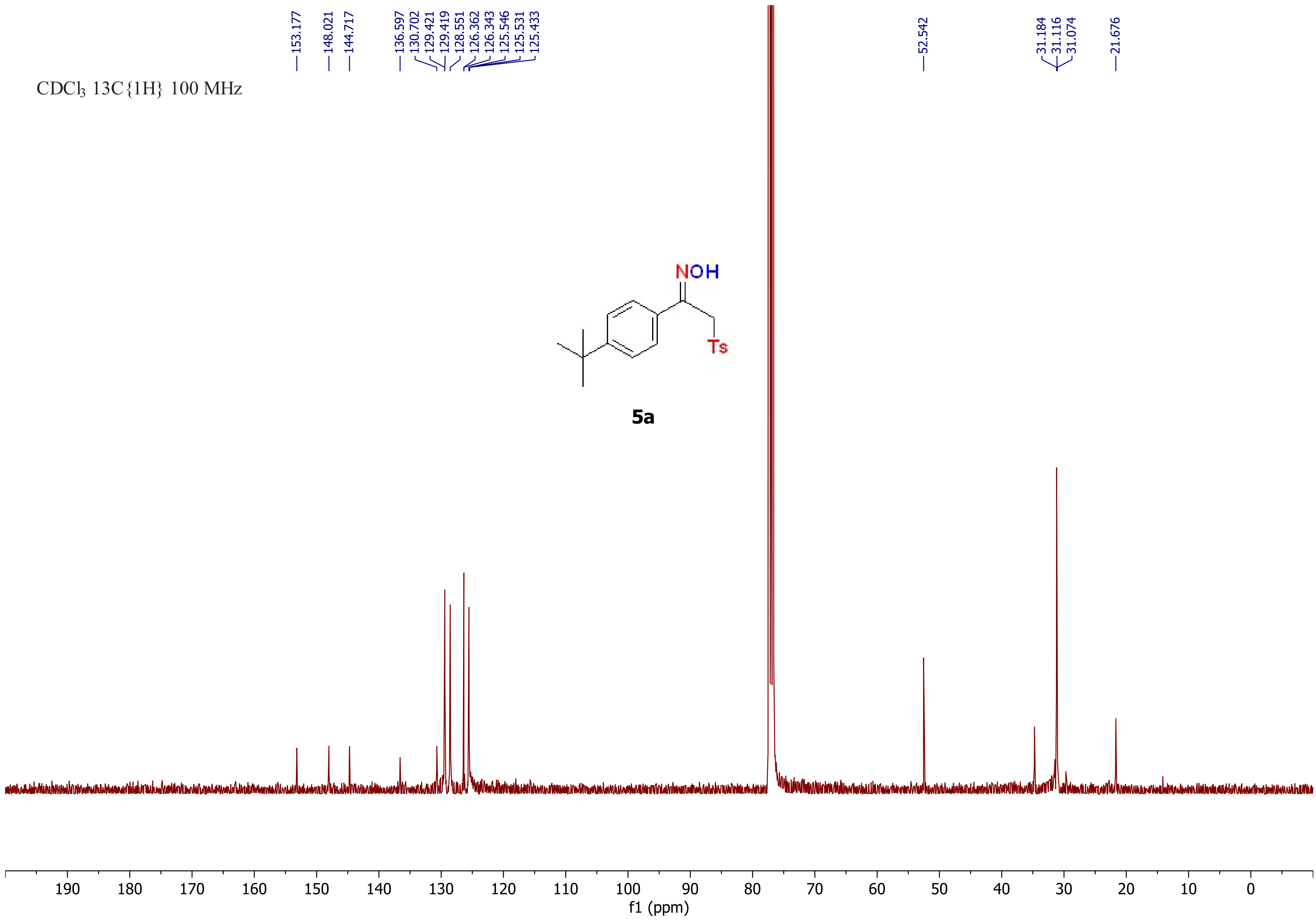
CDCl3 13C{1H} 100 MHz
153.177
148.021
144.717
136.597
130.702
129.421
129.419
128.551
126.362
126.343
125.546
125.531
125.433
52.542
31.184
31.116
31.074
21.676
NOH
Ts
5a
190 180 170 160 150 140 130 120 110 100 90 80 70 60 50 40 30 20 10 0
f1 (ppm)
